# Rotenoids and Isoflavones from *Xeroderris stuhlmannii* (Taub.) Mendonça & E.P. Souza and Their Biological Activities

**DOI:** 10.3390/molecules28062846

**Published:** 2023-03-21

**Authors:** Livie Blondèle Kenou Mekuete, Willifred Dongmo Tékapi Tsopgni, Augustine Kuinze Nkojap, Jacquy Joyce Wanche Kojom, Timo D. Stark, Yannick Fouokeng, Alain Bertrand Dongmo, Léon Tapondjou Azeufack, Anatole Guy Blaise Azebaze

**Affiliations:** 1 Research Unit of Environmental and Applied Chemistry, Faculty of Science, University of Dschang, Dschang P.O. Box 67, Cameroon; kenoulivie14@gmail.com (L.B.K.M.); tapondjou2001@yahoo.fr (L.T.A.); 2 Department of Chemistry, Faculty of Sciences, University of Douala, Douala 24157, Cameroon; willifred2kpi@yahoo.fr (W.D.T.T.); yfouokeng@gmail.com (Y.F.); azebaze@gmail.com (A.G.B.A.); 3 Lehrstuhl für Lebensmittelchemie und Molekulare Sensorik, Technische Universität München, 85354 Freising, Germany; 4 Department of Animal Biology and Physiology, Faculty of Sciences, University of Douala, Douala 24517, Cameroon; augustine_kuinze@yahoo.fr (A.K.N.); kojomjoyce@yahoo.fr (J.J.W.K.); alainberd@yahoo.fr (A.B.D.)

**Keywords:** *Xeroderris stuhlmannii*, *Fabaceae*, rotenoids, isoflavones, antibacterial, antifungal activities

## Abstract

The phytochemical study of the ethanolic extract of the leaf of *Xeroderris stuhlmannii* led to the isolation of five hitherto unreported compounds including two isoflavones (**1**–**2**), and three rotenoids (**3**–**5**), along with eight known isoflavonoid derivatives (**6**–**13**) and one pterocarpan derivative (**14**). The structures of the new compounds and those of the known ones were established by the spectroscopic (1D and 2D NMR) and spectrometric (HRESIMS) techniques as well as a comparison of their spectroscopic data with those reported in the literature. The leaf extract, fractions, and isolated compounds were tested for their antibacterial effects against nine bacterial strains. Compounds **3**, **8**, **11,** and **12** showed a significant antibacterial effect, with a minimum inhibitory concentration (MIC) value of 62.5 µg/mL each, against *Salmonella typhi*, *Staphylococcus aureus*, *Klessiella pneumonae*, and *Escherichia coli*, respectively. In addition, the leaf extract, fractions, and isolated compounds were tested for their antifungal effects against four fungal strains. The hexane fraction showed a significant antifungal effect with an MIC value of 125 µg/mL against *Candida parasilosis*, whereas compounds **3**, **8**, and **12** showed significant antifungal activity with an MIC value of 62.5 µg/mL, each against *Candida parasilosis*, *Candida albicans*, and *Candida krusei*, respectively.

## 1. Introduction

*Xeroderris* genus belongs to the *Fabaceae* family, commonly known as *Leguminoseae*, and is an extremely rich source of biologically active compounds mainly flavonoid derivatives, which is the major class of secondary metabolites found in the family [1,2]. These bioactive phytochemicals possess antibacterial, antioxidant, antifungal, and antimalarial activities [3,4,5]. Despite the existing various solutions for health care, drug resistance is common. Thus, the research of new compounds to tackle biological resistance is urgent [6]. Based on the various diversity of secondary metabolites from the *Fabaceae* family [3] and the ethnopharmacological report on *Xeroderris stuhlmannii* (Taub) Mendonça and Souza, we examined the leaves of *X. stuhlmannii*, one of the two species of *Xeroderris* genus [7]. *X. stuhlmannii* grows in the West Region of Cameroon and is also called “wing pod” or “wing bean” in English and “mumundu” in the South–West region of Cameroon [8,9]. *X. stuhlmannii* is a tree, growing up to 18–27 m tall and 120 cm in diameter. Its leaves are alternate at the tips of the branches and are 6–12 mm long, while fruits come in the form of a linear-oblong, globous, and contain one or two bean-shaped seeds [10]. The leaves of *X. stuhlmannii* are used in traditional folk medicine to treat colds and stomach pains, while the boiled roots are used to fight against malaria [4,9]. LC-MS analysis of the bark extract of *X. stuhlmannii* proposed various classes of secondary metabolites such as rotenoids, flavonoids, polyphenols, tri-terpenoids, steroids, and other phytochemicals. Moreover, bark extract indicated antibacterial activity on *Salmonella appendicitis*, Coliform, *Staphylococcus aureus*, *Pseudomonas aeruginosa,* and *Escherishia coli* [5]. We report, herein, the isolation and structure determination of isoflavones and rotenoids derivatives from the ethanolic leaf extract of *X. stuhlmannii*. In addition, antibacterial, antifungal, and antioxidant activities of leaf extract, fractions, and some isolated compounds were also evaluated.

## 2. Results and Discussion

### 2.1. Structural Elucidation of the Isolated Compounds

**Figure 1:**
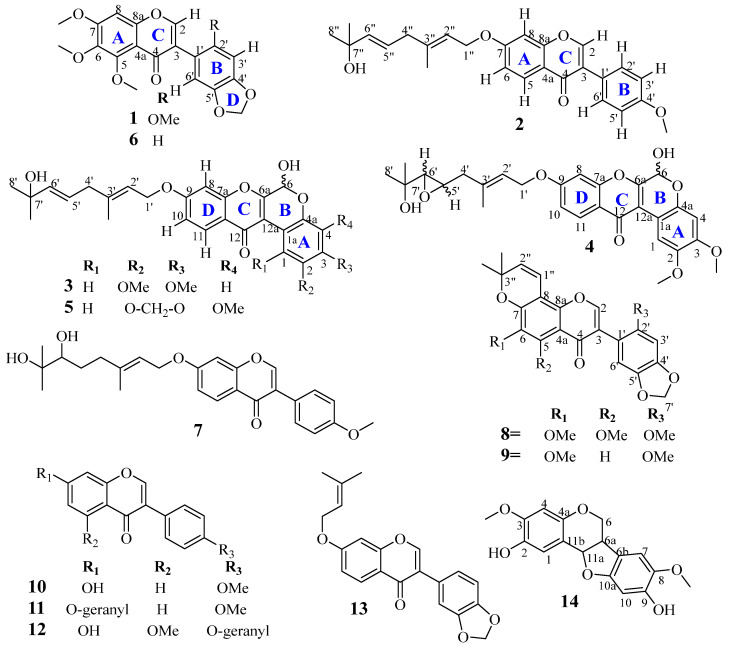
Structures of isolated compounds (**1**–**14**).

The ethanolic extract of the leaf of *X. stuhlmannii* was fractionated and purified by successive chromatographic techniques to afford two new isoflavones (**1**–**2**), three new rotenoids (**3**–**5**) (Figure 1), together with nine known compounds (**6**–**14**). All the compounds were characterized and identified by the spectroscopic (1D and 2D NMR) and spectrometric (HRESIMS) techniques, as well as a comparison of their spectroscopic and physical data with that published in the literature. The known isolated compounds were identified as odoratin (**6**) [1], griffonianone D (**7**) [11], conrauinone A (**8**) [12], ichthynone (**9**) [13,14], formononetin (**10**) [15], 7-*O*-geranylformononetin (**11**) [16], conrauinone C (**12**) [17], maximaisoflavone B (**13**) [18], and abrusprecatin A (**14**) [19].

**Figure 2:**
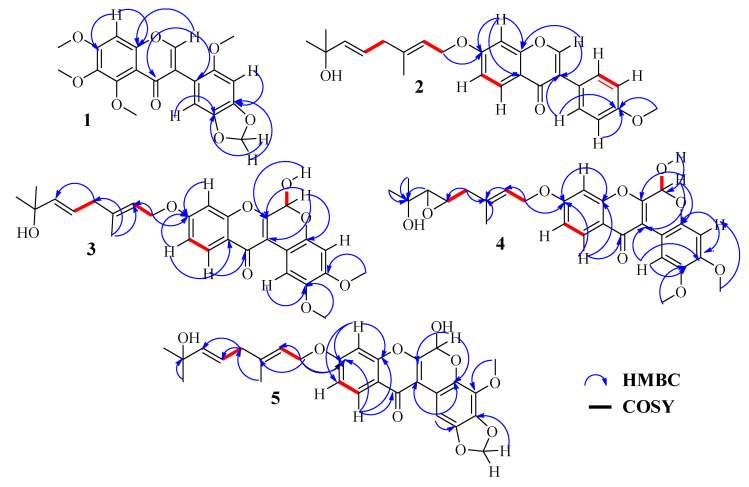
Key HMBC and ^1^H-^1^H COSY correlations for compounds **1**–**5**.

Compound **1** was obtained as a white powder, and gave a positive test with Shinoda reagent. Its molecular formula C_20_H_18_O_8_ was established from the (+)-HRESIMS which showed the pseudomolecular ion peak [M+H]^+^ at *m*/*z* 387.1082 (calcd. for C_20_H_19_O_8_ 387.1080), indicating 12 double bond equivalents. Its ^1^H NMR spectrum (Table 1) exhibited proton signals of flavonoids, and the signal depicted at *ẟ*_H_ 7.80 (1H, s, H-2), characteristic of the proton H-2 of the isoflavonoid skeleton [20]. This spectrum further displayed a singlet for an aromatic proton at *ẟ*_H_ 6.72 (1H, s, H-8), together with a singlet of a methylene dioxyl group at *ẟ*_H_ 5.97 (2H, s, O-CH_2_-O) and three sharps singlets at *ẟ*_H_ 3.98 (3H, s), 3.97 (3H, s), 3.75 (3H, s), a set of signals characteristic of an odoratin derivative [1]. In addition, the ^1^H NMR spectrum displayed one more singlet for the methoxyl group at *ẟ*_H_ 3.93 (3H, s) and two more aromatic proton singlets at *ẟ*_H_ 6.63 (1H, s) and 6.85 (1H, s), assignable to two *para*-coupled protons of the B-ring. The broadband decoupled ^13^C NMR spectrum combined with the DEPT 135 spectrum displayed 20 carbon signals including the characteristic signals of an isoflavone skeleton at *ẟ*_C_ 175.0 (C-4) and 152.5 (C-2). The other carbon signals were grouped as six aromatic carbons, seven oxygenated aromatic carbons, four methoxyl carbons, and one methylene dioxyl carbon. In the ^13^C NMR spectrum, the occurrence of all quaternary oxygenated sp^2^ carbons in the range of 140–150 ppm suggested the presence of oxygen on adjacent carbons (namely, C-4′and C-5′, C-6, and C-7) [21]. The assignation of the singlet resonance at *ẟ*_H_ 6.72 to the aromatic proton H-8 was supported by the HMBC correlations between H-2 (*ẟ*_H_ 7.80), H-8 (*ẟ*_H_ 6.72), and carbon C-8a at *ẟ*_C_ 154.6. Whereas that at *ẟ*_H_ 6.85 was assigned to H-6′ on the basis of the strong HBMC correlation between H-6′, H-2 and carbon C-3 at *ẟ*_C_ 122.5. Therefore, the *para* coupling between H-6′ at *ẟ*_H_ 6.85 (1H, s) and H-3′ at *ẟ*_H_ 6.63 (1H, s) inferred the positions of the methylene dioxyl to be C-4′ and C-5′ and that of the fourth methoxyl group C-2′. The HMBC spectrum (Figure S5) further supported these positions by the neighboring correlations (Figure 2) among H-3′ and H-6′ with carbons C-3 (*ẟ*_C_ 122.5), C-5′ (*ẟ*c 141.1), C-4′ (*ẟ*c 148.4), and then the correlation between 2′-OMe with C-2′ (*ẟ*_C_ 140.5). Consequently, the structure of **1** was elucidated as 5,6,7-trimethoxy-3-(6-methoxybenzo[*d*][1,3]dioxol-5-yl)-4*H*-chromen-4-one, which we trivially named Stuhlmannione A.

Compound **2** was obtained as a white powder and gave a positive test with Shinoda reagent. Its molecular formula C_26_H_28_O_5_ was deduced from the (+)-HRESI-MS, which highlighted a protonated molecular ion peak at *m*/*z* 421.2023 [M+H]^+^ (calcd. for C_26_H_29_O_5_ 421.2015). The analysis of the low field region of 1D NMR and HSQC experiment pointed out a signal H-2/C-2 at 7.94 (1H, s)/*ẟ*c 152.0 suggestive of an isoflavone type skeleton [20]. Its ^1^H NMR spectrum also showed three aromatic protons consistent in an ABX system at *ẟ*_H_ 7.01 (dd, *J* = 8.9, 2.3 Hz, H-6), 8.22 (d, *J* = 8.9 Hz, H-5) and 6.88 (d, *J* = 2.3 Hz, H-8). The ABX system was easily located on the A-ring, due to the deshielded value of H-8 induced by the anisotropic effect of the carbonyl C-4. Moreover, the AA’BB’ spin coupled system appearing at *ẟ*_H_ 7.52 (2H, dd, *J* = 8.8 Hz, H-2′/6′) and *ẟ*_H_ 6.99 (2H, dd, *J* = 8.8 Hz, H-3′/5′) indicated a *para*-substituted B-ring. Furthermore, the ^1^H NMR spectrum displayed singlet protons at *ẟ*_H_ 3.86 (3H, s) for the O-Me group, while a side-chain moiety was shown by ^13^C (Table 1) and DEPT data analysis to contain 10 carbons and one hydroxy-group, suggesting the presence of either a geraniol or nerol moiety [22]. Fuendjiep et al. showed that ^13^C data, particularly the chemical shift of the methyl at the CH_3_-9″ and that of the methylene C-4″, aid to distinguish a geranyl side chain [12]. The chemical shift at *ẟ*_C_ 16.8 and 42.2 ppm observed for methyl and methylene, respectively, combined with the biogenetic consideration confirmed the presence of a geraniol side chain in compound **2** [12]. The positions of these two units were determined using the HMBC spectrum. In fact, the correlation between the methoxy protons at *ẟ*_H_ 3.86 and the aromatic protons H-2′/6′ with carbon C-4′at *ẟ*c 159.6 allowed us to locate the methoxy group at C-4′, and otherwise, the cross-peaks observed between H-8 (*ẟ*_H_ 8.22), the oxymethylenic protons H-1″ (*ẟ*_H_ 4.66) and carbon C-7 (*ẟ*c 163.2) highlighted that the geranyloxy moiety was located at C-7. On the basis of the above spectroscopic evidence, the structure of **2** was deduced to be 7-(((2*E*,5*E*)-7-hydroxy-3,7-dimethylocta-2,5-dien-1-yl)oxy)-3-(4-methoxyphenyl)-4*H*-chromen-4-one and trivially named Stuhlmannione B.

Compound **3** was isolated as yellow powder. Its molecular formula, C_28_H_30_O_8_, was determined from (+)-HRESIMS ion peak at *m*/*z* 477.1931 [M+H-H_2_O]^+^ (calcd. for C_28_H_29_O_7_ 477.1913). The ^1^H NMR spectra displayed a singlet for the deshielded aromatic proton at *ẟ*_H_ 8.51 (1H, s, H-1) and a doublet for the hemiketal proton at *ẟ*_H_ 6.20 (1H, d, *J* = 7.0 Hz), which together with the two methoxy singlet protons at *ẟ*_H_ 3.78 (3H, s) and 3.80 (3H, s) suggested that the structure **3** was similar to that of 6-hydroxy-2,3,9-trimethoxy-[1]-benzopyrano [3,4-b][1]benzopyran-12(6*H*)-one (rotenoid) [23,24]. The ^13^C NMR spectrum of **3** (Table 2) supported the presence of the rotenoid skeleton as it exhibited signals at *ẟ*_C_155.8 (C-6a), 110.1 (C-12a), and 88.7 (C-6). In addition, ^1^H NMR and ^1^H-^1^H COSY spectra of **3** exhibited an ABX system formed by signals at *ẟ_H_* 7.13 (1H, dd, *J =* 8.9, 2.4 Hz, H-10), *ẟ_H_* 7.24 (1H, d, *J* = 2.4 Hz, H-8) and *ẟ_H_* 8.07 (1H, d, *J* = 8.9 Hz, H-11). The ABX system was clearly located on the D-ring, due to the deshielded value of H-11 induced by the anisotropic effect of the carbonyl C-12. Moreover, the ^1^H NMR spectrum displayed one more singlet for aromatic proton at *ẟ_H_* 6.73 (1H, s), which according to the multiplicity was unequivocally located in a *para* position of H-1. Furthermore, a geraniol side chain attached was evident in compound **3** from the ^1^H and ^13^C NMR signals at *δ* 1.17 (6H, s), 1.73 (1H, dd, *J =* 1.3 Hz), 4.73 (2H, m, *J =* 6.5 Hz), 2.74 (2H, d, *J* = 6.7Hz), 5.50 (t), 5.53 (m), and 5.61 (dt), and a hydroxyl signal at *δ* 4.49 (Table 2) and also from the HRESIMS in which a fragment ion at *m*/*z* 153 was observed. The geraniol was evidenced at C-9 by the HMBC correlations depicted between H-11 (*ẟ_H_* 8.07), oxymethylene H-1′ (*ẟ_H_* 4,73), and carbon C-9 (*ẟ*c 163.6), whereas the positions of the two methoxys were deduced from the *para* coupled protons H-1 and H-4, further supported by the HMBC correlations between -OCH_3_-2 (*ẟ_H_* 3.78), H-1 (*ẟ_H_* 8.51), and carbon C-2 (*ẟ_C_* 143.9), and then between -OCH_3_-3 (*ẟ_H_* 3.80), H-4 (*ẟ_H_* 6.73), and C-3 (*ẟ_C_* 149.8). Compound **3** exhibited [α]_D_ = 0, thus, we propose it is racemic at the single chiral center, C-6. On the basis of the above spectroscopic studies, compound **3** was established as 6-hydroxy-9-(((2*E*,5*E*)-7-hydroxy-3,7-dimethylocta-2,5-dien-1-yl) oxy)-2,3-dimethoxy-[1]-benzopyrano [3,4-b[1] benzopyran-12(6*H*)-one, trivially named Stuhlmarotenoid A.

Compound **4** was obtained as a yellow powder. The (+)-HRESIMS *m*/*z* 493.1873 (calcd. for C_28_H_29_O_8_, 493.1863) depicted the molecular formula C_28_H_30_O_9_, whereas the ^1^H NMR spectrum showed a similar pattern to that of **3** through *para* coupled aromatic protons H-1 at *ẟ*_H_ 8.51 (1H) and H-4 at *ẟ*_H_ 6.74 (1H), an ABX system caused by protons H-8 at *ẟ*_H_ 7.24 (1H, d, *J =* 2.4 Hz), H-10 at *ẟ*_H_ 7.13 (1H, dd, *J =* 8.9, 2.4 Hz) and H-11 at *ẟ*_H_ 8.07 (1H, d, *J =* 8.9 Hz), a signal for a hemiketal at *ẟ*_H_ 6.20, and the two methoxy groups -OCH_3_-2 *ẟ*_H_ 3.79 and -OCH_3_-3 *ẟ*_H_ 3.80. The ^13^C NMR data supported the similarity as it displayed carbon signals at *ẟ*_C_155.9 (C-6a), 110.1 (C-12a), and 88.7 (C-6). The side chain geraniol group in **3** differed from that of **4** by the lack of an alkene bond replaced by an epoxide linked to carbons H-5′/C-5′ (*ẟ*_H_ 2.96/*ẟ*_C_ 53.8) and H-6′/C-6′ (*ẟ*_H_ 2.67/*ẟ*_C_ 64.8). The position of the epoxide was supported by the HMBC correlation between H-5′ (*ẟ*_H_ 2.96) and C-3′ (*ẟ*c 141.4), whereas the side chain was clearly located at C-9, by the correlation between H-11 (*ẟ_H_* 8.07), oxymethylene H-1′ (*ẟ_H_* 4,75) and carbon C-9 (*ẟ*c 163.6). The relative configuration of the epoxide in the side chain was deduced to be *α* on the basis of the ^1^H and ^13^C chemical shift values of H-5′ and H-6′ compared to those of reported data [25], further supported by the coupling constants between H-5′ and H-6′ (*J* = 2.2 Hz) and the biogenic consideration. Based on the above spectral evidence, the structure of compound **4** was established as 6-hydroxy-9-(((2*E*)4-(3-(2-hydroxypropan-2-yl)oxiran-2-yl)-3-methylbut-2-en-1-yl) oxy)-2,3-dimethoxychromeno[3,4-b]chromen-12(6*H*)-one and trivially named Stuhlmarotenoid B.

Compound **5** was obtained as a brown oil, and its molecular formula was established as C_28_H_28_O_9_ from its (+)-HRESIMS, which showed a pseudo molecular ion peak [M+Na]^+^ at 531.1630 (calcd. for C_28_H_28_O_9_Na 531.1631). The ^1^H NMR spectrum displayed a signal for a deshieled aromatic proton at *ẟ*_H_ 8.33 (1H, s, H-1) and a signal for an hemiketal proton at *ẟ*_H_ 6.27, which together with signals of geraniol suggested that the structure of **5** was similar to that of **3**. ^13^C NMR supported the similarity as it displayed carbon signals at *ẟ*_C_ 154.5 (C-6a), 110.7 (C-12a) and 89.2 (C-6). The differences between the 1D NMR data of **5** and **3** were the lack of the aromatic proton signal for H-4 and one methoxy group, in contrast the appearance of a proton signal for the methylene dioxyl -OCH_2_-(2/3) at *ẟ*_H_ 6.07/*ẟ*_C_ 102.4. The position of the methylene dioxyl were deduced to be C-2 and C-3 by the HMBC correlation between H-1 (*ẟ*_H_ 8.33), -OCH_2_-(2/3) (*ẟ*_H_ 6.07), and the carbon C-2 (*ẟ*_C_ 140.5) and C-3 (*ẟ*_C_ 136.4), inferring the methoxy group -OCH_3_-4 (*ẟ*_H_ 3.97/*ẟ*_C_ 56.6) to be C-4. The position of the methoxy group -OCH_3_-4 was further supported by the HMBC correlation between methoxy group -OCH_3_-4, H-1 and C-4 (*ẟ*_C_ 139.4). Compound **5** exhibited [α]_D_ = 0, thus, we propose it as racemic at the single chiral center, C-6. The structure of compound **5** was therefore unambiguously established as 6-hydroxy-9-(((2*E*,5*E*)-7-hydroxy-3,7-dimethylocta-2,5-dien-1-yl)oxy)-4-methoxychromeno[2,3-c]-[1,3]-dioxolo[4,5-g]-chromen-12(6*H*)-one and trivially named Stuhlmarotenoid C (**5**).

### 2.2. Biological Activities

#### 2.2.1. Antibacterial Evaluation

The antibacterial effects of the EtOH leaf extract, hexane, and AcOEt fractions as well as all the isolated compounds were evaluated against ATCC strains using the microtiter broth dilution method to determine the MIC and MBC (minimal inhibitory and bactericidal concentration) [26]. The results (Table 3) showed that the ethanol leaf extract exhibited moderate activity against *Pseudomonas aeruginosa* and *Salmonella enteritidis* with MIC values of 250 µg/mL each and bactericidal effects with MBC/MIC ratios of 2 each. Compound **12** indicated moderate activity against *Escherichia coli* with an MIC value of 62.5 µg/mL and weak activity against *Staphylococcus aureus* and *Salmonella typhimurium* with MIC values of 125 µg/mL each. Compound **12** evoked bactericidal effect ratios (MBC/MIC) of 2, 4, and 2, respectively against *S. aureus*, *E. coli*, and *S. typhimurium*. Compound **3** indicated weak activity against *Shigella flexineri* and *Shigella dysenteria* with MIC values of 125 µg/mL, moderate activity against *Salmonella typhi* with MIC value of 62.5 µg/mL, and bactericidal effect ratios (MBC/MIC) of 2, 2, and 4, respectively. Compound **8** highlighted moderated activity against *S. aureus* with an MIC value of 62.5 µg/mL, weak activity against *S. typhi* with an MIC value of 125 µg/mL, and bactericidal effects with MBC/MIC ratios of 4 and 2, respectively. Compound **11** showed moderated activity against *S. aureus* and *Klessiella pneumonae* with MIC values of 62.5 µg/mL, weak activity against *E. coli* with an MIC value of 125 µg/mL and bactericidal effects with MBC/MIC ratios of 4, 2, and 4, respectively. The other compounds were found to be either weakly active or inactive on the tested strains.

#### 2.2.2. Antifungal Evaluation

The antifungal activity of the EtOH leaf extract, hexane, and AcOEt fractions as well as all the isolated compounds were evaluated against the four fungal strains *Candida albicans, Candida krusei*, *Candida parasilosis*, and *Cryptococcus neoformans* following a standard protocol. Table 4 shows the antifungal activity of leaf extract, fractions, and some isolated compounds. The leaf extract was found to be inactive against all the tested strains, whereas the hexane fraction evoked moderate activity against *C. parasilosis* with the MIC value of 250 µg/mL and a fungicidal effect with a ratio MFC/MIC of 2. Compound **12** highlighted moderate activity against *C. krusei* with an MIC value of 62.5 µg/mL, weak activity against *C. parasilosis* and *C. neoformans* with MIC values of 125 µg/mL, and fungicidal effects with MFC/MIC ratios of 4, 2, and 2, respectively. Compound **3** showed moderate activity against *C. parasilosis* with an MIC value of 62.5 µg/mL, weak activity against *C. albicans* and *C. neoformans* with MIC values of 125 µg/mL, and fungicidal effects with MFC/MIC ratios of 2, 4, and 2, respectively. Compound **6** revealed weak activity against *C. parasilosis* and *C. neoformans* with MIC values of 125 µg/mL and fungicidal effects with MFC/MIC ratio of 2 each. Compound **8** was evaluated with moderate activity against *C. albicans* with an MIC value of 62.5 µg/mL, weak activity against *C. krusei* with an MIC value of 125 µg/mL, and fungicidal effects with MFC/MIC ratios of 4 and 2, respectively. The other compounds were found to be either weakly active or inactive on the tested strains.

#### 2.2.3. Antioxidant Evaluation

It has been demonstrated that more than one method is necessary to elucidate the antioxidant capacity of samples because these assays differ in the principles and experimental conditions. In this study, the antioxidant activity of the ethanolic leaf extract, hexane, and AcOEt fractions as well as the isolated compounds were tested using the radical scavenging activities and reducing power as listed in Table 5. The ethanolic leaf extract showed significant scavenging activity against 2,2-azino-bis (3-ethylbenzothiazoline-6-sulfonic acid) (ABTS) with RS_a50_ of 13.44 ± 0.82 and 2,2-dipheny-1-picrylhydrazyl (DPPH) with RS_a50_ of 14.97 ± 0.86 µg/mL, while a poor reducing power was observed within the ferric reducing antioxidant power (FRAP) assay. These results suggest that the ethanol leaf extract contains compounds that could act as a free radical scavenger that is, capable of donating hydrogen or electron to a free radical in order to stabilize the odd electron which is responsible for radical’s reactivity [27]. The fractions and isolated compounds were found to be either weakly active or inactive. The absence of activity in the isolated compounds may be due to the lack of proton donors in almost all their structures.

Although the known compounds displayed either weak or no activity in this work, griffonianone D (**7**) was reported to possess anti-inflammatory effects in different experimental models of inflammation [11], while ichthynone (**9**) displayed a weak cytotoxic effect on cancer cells. [28]. Formonotin (**10**) was reported to exhibit anticancer activity [29], whereas maximaisoflavone B (**13**) and abrusprecatin (**14**) exhibit weak antiplasmodial activities [19,30]. To the best of our knowledge, no biological activity is reported for odoratin (**6**), conrauinone (**8**), 7-*O*-geranylformononetin (**11**), and conrauinone C (**12**).

## 3. Materials and Methods

### 3.1. General Experimental Procedures

HR-ESI were generated on either an Agilent 6220 TOF LCMS mass spectrometer (Agilent Technologies, Santa Clara, CA, USA) with perfluorokerosene as reference substance or Synapt G2-S (Waters, Manchester, UK, Details in the Supplementary Materials) for ESI-HR-MS. The NMR spectra were recorded at 500 MHz for ^1^H and 125 MHz for ^13^C, on Bruker DRX 500 or Avance NEO spectrometer with a cryo probe CTCI (^1^H/^13^C/^15^N) (Bruker, Rheinstetten, Germany) in CDCl_3_, C_3_D_6_O, and DMSO-*d_6_*. All the chemical shifts are given in *ẟ* (ppm) with reference tetramethylsilane (TMS) (Sigma-Aldrich, Munich, Germany) to the residual solvent signal, while coupling constants (*J*) were measured in Hz. Data processing and evaluation were performed using the Topspin Software Version 4.0.7 (Bruker, Rheinstetten, Germany). For the determination of the specific optical rotation, the polarimeter Jasco P-2000 (Jasco, Groß-Umstadt, Germany) digital polarimeter with a 5-cm cuvette was employed. The aperture was set to 1.8 mm. Column chromatography was performed using Sephadex LH-20 gel (Amersham Pharmacia Biotech, Uppsala, Sweden) and silica gel 60 (0.040–0.063 mm, Merck, Darmstadt, Germany). Preparative HPLC was performed on a Jasco System (Jasco, Groß-Umstadt, Germany, see details the Supplementary Materials). Thin layer chromatography (TLC) was carried out on silica gel 60 F_254_ (Merck, Darmstadt, Germany) plates developed with hexane-EtOAc, EtOAc, and EtOAc-MeOH. While spots were detected by spraying with 10% H_2_SO_4_ reagent followed by heating. The molecular composition of the isolated compounds was identified by accurate mass determinations. All reagents used were of analytical grade.

### 3.2. Plant Material

The leaves of *X. stuhlmannii* were collected in Tonga, west region of Cameroon, in November 2020. The plant was identified by Tacham Walter of National Herbarium of the Cameroon, where a voucher specimen (No. 6011/SRF/CAM) has been deposited.

### 3.3. Extraction and Isolation

The air-dried leaves of *X. Stuhlmannii* (1.5 kg) were powdered prior to being extracted with EtOH (10 L × 3) at room temperature. The filter solution was concentrated in vacuo to afford leaf crude extract (130.0 g). The crude extract was suspended in water and partitioned with *n*-hexane (Hex), EtOAc, and MeOH (1 L, three times, each) to yield *n*-hexane (HF, 50.8 g), EtOAc (EF, 30.4g), and MeOH (MF, 15.6 g) fractions, respectively. The EtOAc fraction which contained less chlorophyll was further investigated. An aliquot of 28.0 g of the EtOAc fraction was subjected to silica gel column chromatography (CC) (100 × 5 cm) and eluted with gradients of *n*-hexane-EtOAc (from 1:9 to 100:00 (*v*/*v*)) and EtOAc-MeOH (from 5:95 to 15:85 (*v*/*v*)), 1 L each. The resulting subfractions were grouped into four fractions FA1-FA4, on the basis of their TLC profiles. Subfraction FA1 (100.5 mg, Hex–EtOAc (4:1, *v*/*v*)), was further chromatographed on silica gel (100 × 2.5 cm) with an isocratic solvent system of Hex–EtOAc (85:15, *v*/*v*) to give compounds **6** (10.2 mg), **8** (15.1 mg), and **11** (30.3 mg). Subfraction FA2 (90.5 mg, Hex–EtOAc (7:3, *v*/*v*)), was further chromatographed by Sephadex LH-20 cc (100 × 2.5 cm) with 100% MeOH (500 mL) to yield compounds **2** (8.15 mg) and **12** (12.25 mg), also subfractions FA2a (20.15 mg) and FA2b (10.25 mg). Subfraction FA2a was purified by HPLC using a preparative RP-C18 column (CH_3_CN− H_2_O, 55:45), to yield compound **1** (15.2 mg), whereas FA2b was purified by preparative silica gel TLC with a solvent system of Hex–EtOAc (3:2, *v*/*v*) to give compounds **13** (6.1 mg). Subfraction FA3 (200.5 mg, Hex–EtOAc (3:2, *v*/*v*)), was further chromatographed by Sephadex LH-20 column chromatography (100 × 2.5 cm) with 100% MeOH (400 mL) to give subfractions FA3a (25.4 mg), FA3b (20.3 mg), and FA3c (10.5 mg). Subfraction FA3a was purified by HPLC using a preparative RP-C18 column (CH_3_CN− H_2_O, 55:45) to yield compounds **7** (8.2 mg) and **10** (8.3 mg). Using the same conditions mentioned above, FA3b was purified by HPLC using a preparative RP-C18 column (CH_3_CN− H_2_O, 55:45) to yield compounds **5** (10.6 mg) and **9** (5.2 mg), whereas FA3c was purified by preparative silica gel TLC with a solvent system of Hex–EtOAc (1:1, *v*/*v*) to give compound **14** (7.5 mg). FA4 (80.5 mg, Hex–EtOAc (3:7, *v*/*v*)), was further chromatographed by Sephadex LH-20 column chromatography (100 × 2.5 cm) with 100% MeOH (400 mL), then preparative silica gel TLC with a solvent system of Hex–EtOAc (3:7, *v*/*v*) to give compounds **3** (12.5 mg) and **4** (10.1 mg).

*Stuhlmannione A* (**1**). Yellow powder; HRESI-MS *m*/*z* 387.1081 [M+H]^+^ (calcd. for C_20_H_19_O_8_ *m*/*z* 387.1080); ^1^H-NMR (500 MHz, CDCl_3_) and ^13^C-NMR (125 MHz, CDCl_3_): see Table 1.

*Stuhlmannione B* (**2**). Yellow powder; HRESI-MS *m*/*z* 421.2023 [M+H]^+^ (calcd. for C_26_H_29_O_5_ *m*/*z* 421.2015); ^1^H-NMR (500 MHz, CDCl_3_) and ^13^C-NMR (125 MHz, CDCl_3_): see Table 1.

*Stuhlmarotenoid A* (**3**). Yellow powder; [α]_D_ = 0°; HRESI-MS *m*/*z* 477.1931 [M-H_2_O-H]^+^ (calcd. For C_28_H_29_O_7_ *m*/*z*477.1913); ^1^H-NMR (500 MHz, DMSO-*d_6_*) and ^13^C-NMR (125 MHz, DMSO-*d_6_*): see Table 2.

*Stuhlmarotenoid B* (**4**). Yellow powder; HRESI-MS *m*/*z* 493.1873 [M-H_2_O-H]^+^ (calcd. for C_28_H_29_O_8_ *m*/*z* 493.1862); ^1^H-NMR (500 MHz, DMSO-*d_6_*) and ^13^C-NMR (125 MHz, DMSO-*d_6_*): see Table 2.

*Stuhlmarotenoid C* (**5**). Brown oil; [α]_D_ = 0°; HRESI-MS *m*/*z* 531.1630 [M+Na]^+^ (calcd. for C_28_H_28_O_9_Na *m*/*z* 531.1631); ^1^H-NMR (500 MHz, CDCl_3_) and ^13^C-NMR (125 MHz, CDCl_3_): see Table 2.

### 3.4. Biological Activities

#### 3.4.1. Antibacterial Activity

The antibacterial activity was implemented against nine strains (*Pseudomonas aeruginosa* (ATCC01); *Staphylococcus aureus* (ATCC25922); *Escherishia coli* (ATCC10536); *Klessiella pneumonae* (ATCC13883); *Shigella flexineri*; *Shigella dysenteria*; *Salmonella typhimurium*; *Salmonella typhi* (ATCC6539), and *Salmonella enteritidis*). The broth microdilution method was used for susceptibility testing of bacteria species in 96-well microtiter sterile plates as previously described [26,31,32]. Briefly, the crude extracts were dissolved in 5% DMSO solution and diluted with Mueller Hinton broth to obtain a stock concentration of 2000 µg/mL for the extracts, 1000 µg/mL for fractions, and 500 µg/mL for the isolated compounds. This gave a concentration range of 1000–0.96 µg/mL, 500–0.96 µg/mL, and 250–0.96 µg/mL respectively. One hundred microliters of each bacterial suspension (containing about 1.5 × 10^6^ CFU/mL) was added, respectively, to the wells containing the test samples and mixed thoroughly to give final concentrations ranging from 500 to 0.48 µg/mL for extract, 250 to 0.48 µg/mL for fraction, and 125 to 0.48 µg/mL for isolated compounds. Ciprofloxacin^®^ (Bayer, Leverkusen, Germany) at concentration of 125–0.48 µg/mL was used as the standard reference. The assay microtiter plates were incubated at 37 °C for 24 h. Inhibitory concentrations of the extracts were detected after addition of 50 µL to 0.2 mg/mL p-iodonitrotetrazolium chloride (INT) (Sigma–Aldrich, Johannesburg, South Africa) and incubated at 37 °C for 30 min. These preparations were further incubated at 37 °C for 48 hrs, and bacterial growth was revealed by the addition of INT as mentioned above. The smallest concentration at which no color change was observed was considered as the MBC. The tests were performed in duplicates. The ratio MBC/MIC was calculated to determine the bactericidal (MBC/MIC ≤ 4) and bacteriostatic (MBC/MIC > 4) effects.

#### 3.4.2. Antifungal Activity

The inocula of yeasts were prepared from 48 h old cultures by picking numerous colonies and suspending them in sterile saline (NaCl) solution (0.9%). Absorbance was read at 530 nm and adjusted with the saline solution to match that of a 0.5 McFarland standard solution, corresponding to about 10^6^ yeast cells/mL (CLSI, CLSI, formerly national committee for clinical and laboratory standards, NCCLS, 2008). MIC of each extract was determined by using broth microdilution techniques according to the guidelines of CLSIfor yeasts (M27-A2). Stock solutions of the test extracts were prepared in 5% aqueous DMSO solution and diluted with sabouraud dextrose broth (SDB) to give a concentration of 1 mg/mL. This was serially diluted two-fold to obtain a concentration range of 500–0.24 μg/mL for extracts and 125–0.24 µg/mL for compounds. The final concentration of DMSO in the well was less than 1% (preliminary analysis with 1% DMSO did not inhibit the growth of the test organisms). The plates were covered with a sterile lid and incubated on the shaker at 37 °C for 48 h (for yeasts) or at 28 °C for 7 days (for dermatophytes) [33,34,35]. MICs were assessed visually after the corresponding incubation period and were taken as the lowest product concentration at which there was no or virtually no growth. The assay was repeated three times. Nystatin (for yeasts) and griseofulvin (for dermatophytes) were used as positive controls.

#### 3.4.3. Antioxidant Activity

- DPPH radical scavenging assay

The free radical scavenging activities of the sample were evaluated using the DPPH analysis as described by Noghogne et al. [36]. The radical scavenging activities of crude extract were evaluated through spectrophotometer using the 1,1-diphenyl-2-picrylhydrazyl (DPPH) free radical. When DPPH reacts with an antioxidant sample, which can donate hydrogen, it is reduced. The changes in color were measured at wave length 517 nm under UV/visible light spectrophotometer (Infinite M200, TECAN, Männedorf, Switzerland). The extract (1000 µg/mL) was two-fold serially diluted with methanol. Fifty microliters of the diluted extract (1000 µg/mL) in methanol were mixed with 150 µL of 0.02% of 2,2-diphenyl-1-picrylhydrazyl (DPPH) methanol solution, giving a final extract concentration range from 250 to 1.9531 µg/mL (250, 125, 62.5, 31.25, 15.625, 7.8125, 3.9062, and 1.9531 µg/mL). After 30 min of incubation in the dark at room temperature, the optical density was measured. Ascorbic acid (Vitamin C) was used as positive control. Each assay was done in triplicate, and the results recorded as the mean ± standard deviation (SD). The radical scavenging activity (RSA, %) was calculated as follows:

(1) $$\mathrm{RSA}\;\left(\%\right)=\frac{\mathrm{Absorbance}\;\mathrm{of}\;\mathrm{DPPH}\;–\;\mathrm{Absorbance}\;\mathrm{of}\;\mathrm{sample}}{\mathrm{Absorbance}\;\mathrm{of}\;\mathrm{DPPH}}\times 100$$

- ABTS radical scavenging assay

The radical scavenging activities of the samples were evaluated spectrophotometrically using the 2,2′-azino-bis (3-ethylbenzothiazoline-6-sulphonic) acid (ABTS) free radical [37]. When ABTS reacts with an antioxidant compound, which can donate hydrogen, it is reduced. The changes in color were measured at 734 nm under UV/visible light spectrophotometer (Infinite M200 (TECAN, Männedorf, Switzerland). Pure methanol was used to calibrate the counter. The extract (1000 µg/mL) was twofold serially diluted with methanol. Twenty-five microliters of the diluted extract were mixed with seventy-five µL of 2,2′-azino-bis (3-ethylbenzothiazoline-6-sulphonic) acid (ABTS) methanol solution, to give a final extract concentration range of 250–1.9531 µg/mL (250, 125, 62.5, 31.25, 15.625, 7.8125, 3.9062, and 1.9531 µg/mL). After 30 min of incubation in the dark at room temperature, the optical densities were measured at 734 nm. Ascorbic acid (Vitamin C) was used as a control. Each assay was done in triplicate, and the results, recorded as the mean ± standard deviation (SD) of the three findings, were presented in tabular form. The radical scavenging activity (RSA, in %) was calculated as follows:

(2) $$\mathrm{RSA}\;\left(\%\right)=\frac{\mathrm{Absorbance}\;\mathrm{of}\;\mathrm{ABTS}\;–\;\mathrm{Absorbance}\;\mathrm{of}\;\mathrm{sample}}{\mathrm{Absorbance}\;\mathrm{of}\;\mathrm{ABTS}}\times 100$$

- FRAP assay

The ferric reduction potential (conversion potential of Fe^3+^ to Fe^2+^) of the samples was determined according to the method described by Padmaja et al. [38]. Briefly, the samples were first dissolved as for the DPPH assay. 25 µL from each dilution was introduced into a new microplate, and 25 µL of 1.2 mg/mL Fe^3+^ solution was added. The plates were pre-incubated for 15 min at ambient temperature. After this time, 50 µL of 0.2% ortho-phenanthroline was added to obtain final extract concentrations of 250, 125, 62.5, 31.25, 15.625, 7.8125, 3.90625, and 1.95325 µg/mL. The reaction mixtures were further incubated for 15 min at ambient temperature after which the absorbance was measured at 505 nm under UV/visible light spectrophotometer (Infinite M200 TECAN, Männedorf, Switzerland) against the blank (made of 25 µL methanol + 25 µL Fe^3+^ + 50 µL ortho-phenanthroline). Ascorbic acid (Vitamin C) was used as a positive control. The assay was performed in triplicate. From the obtained OD (optical density), reducing percentages were calculated for each concentration and used to determine the RC_50_ from dose–response curves.

## 4. Conclusions

In summary, we have conducted the successful isolation of 15 compounds, including two new isoflavone derivatives (**1**–**2**) and three new rotenoid derivatives (**3**–**5**), together with nine known compounds (**6**–**14**) from *X. stuhlmannii.* The biological evaluations revealed that the leaf extract showed a general trend to exhibit weaker antibacterial and antifungal activities than the hexane fraction. From the evident results, compounds **3**, **12,** and **8** highlighted the greatest antibacterial and antifungal activities. However, no antioxidant activity was observed with the isolated compounds. From these results, further study may be done to confirm the use of the leaves in traditional folk medicine to treat diarrhoea, coccidiosis, endoparasites, fungal infections, and lethargic birds.

## Figures and Tables

**Table 1:** ^1^H-(500 MHz) and ^13^C (125 MHz) NMR spectroscopic data of **1** and **2** recorded in CDCl_3_ (*ẟ* in ppm).

	Compound 1			Compound 2		
Position	*ẟ*_C_	*ẟ*_H_	HMBC	*ẟ*_C_	*ẟ*_H_	HMBC
**2**	152.5	7.80	3, 4, 8a, 1′	152.0	7.94	3, 4, 8a, 1′
**3**	122.5	-		124.3	-	
**4**	175.0	-		175.9	-	
**4a**	113.7	-		118.4	-	
**5**	153.0	-		127.8	8.22	4, 7, 8, 8a
**6**	152.9	-		115.0	7.01	8, 4a
**7**	157.6	-		163.2	-	
**8**	96.1	6.72	4, 6, 7	100.9	6.88	7, 4a, 8a
**8a**	154.6	-		157.9	-	
**1′**	112.8	-		124.9	-	
**2′**	140.5	-		130.2	7.52	3, 3′, 4′
**3′**	95.3	6.63	2′, 4′, 5′	114.0	6.99	1′, 2′, 4′, 5′
**4′**	148.4	-		159.6	-	
**4′-OMe**	-	-		55.4	3.86	4′
**5′**	141.1	-		114.0	6.99	1′, 2′, 4′, 5′
**6′**	111.4	6.85	2′, 4′, 5′	130.2	7.52	3, 3′, 4′
**O-CH_2_-O**	101.3	5.97	4′, 5′	-	-	
**5-OMe**	56.3	3.98	5	-	-	
**6-OMe**	56.8	3.75	6	-	-	
**7-OMe**	62.1	3.97	6, 7	-	-	
**2′-OMe**	61.6	3.93	2′	-	-	
**1″**	-	-		65.5	4.66	7, 2″, 3″
**2″**	-	-		119.3	5.55	3″-Me, 3″, 4″
**3″**	-	-		141.1	-	
**3″-Me**	-	-		16.8	1.78	2″, 3″, 4″
**4″**	-	-		42.2	2.82	2″, 3″-Me, 6″
**5″**	-	-		140.5	5.70	4″, 6″, 7″
**6″**	-	-		123.9	5.66	4″
**7″**	-	-		70.7	-	
**8″**	-	-		29.9	1.35	6″, 7″

**Table 2:** ^1^H-(500 MHz) and ^13^C (125 MHz) NMR spectroscopic data of **3** and **4** recorded in DMSO-d_6_ and **5** recorded in CDCl_3_ (ẟ in ppm).

	Compound 3			Compound 4			Compound 5		
Position	*ẟ*_C_	*ẟ*_H_	HMBC	*ẟ*_C_	*ẟ*_H_	HMBC	*ẟ*_C_	*ẟ*_H_	HMBC
**1a**	108.8	-		108.8	-		127.1	-	
**1**	110.3	8.51	2, 3, 12a	110.3	8.51	2, 3, 12a	105.4	8.33	1a, 2, 4a, 12a
**2**	143.9	-		143.9	-		140.5	-	
**2-OMe**	56.5	3.78	2	56.1	3.79	2	-	-	
**3**	149.8	-		149.7	-		136.4	-	
**3-OMe**	56.1	3.80	3	56.5	3.80	3	-	-	
**4**	102.2	6.73	1a, 2, 3	102.2	6.74	1a, 2, 3	139.4	-	
**(2/3)-OCH_2_**	-	-		-	-		102.4	6.07	3
**4a**	143.8	-		143.8	-		127.2	-	
**4-OMe**	-	-		-	-		56.6	3.97	4
**6**	88.7	6.20	4a, 6a, 12a	88.7	6.20	4a, 6a, 12a	89.2	6.27	4a, 6a
**6-OH**	-	7.97	6a	-	7.98	6, 6a	-	n. d	
**6a**	155.8	-		155.9	-		154.5	-	
**7a**	156.8	-		156.8	-		156.5	-	
**8**	101.7	7.24	7a, 9, 10, 12	101.7	7.24	7a, 9	100.8	6.89	7a, 9, 10
**9**	163.6	-		163.6	-		163.5	-	
**10**	116.0	7.13	8, 9, 11a	116.0	7.13	9, 11a	115.7	7.02	8, 11a
**11**	127.3	8.07	7a, 9, 12	127.4	8.07	7a, 9, 12	127.5	8.17	7a, 9, 12
**11a**	117.8	-		117.8	-		118.0	-	
**12**	174.8	-		174.8	-		175.4	-	
**12a**	110.1	-		110.1	-		110.7	-	
**1′**	66.0	4.73	9, 2′, 3′	65.7	4.75	9, 2′	65.7	4.69	9, 2′, 3′
**2′**	119.5	5.50	1′, 3′-Me, 3′	119.4	5.51	1′, 3′	119.3	5.54	3′-Me, 4′
**3′**	141.4	-		141.4	-		141.2	-	
**3′-Me**	17.0	1.73	2′, 3′, 4′	17.0	1.78	3′	16.8	1.80	2′, 4′, 5′
**4′**	41.9	2.74	3′-Me, 3′, 5′	41.6	2.74/2.25	3′	42.1	2.83	2′, 3′-Me, 5′, 6′
**5′**	122.6	5.53	4′, 7′	53.8	2.96	4′	140.5	5.71	4′, 7′, 8′
**6′**	142.0	5.61	4′, 5′, 7′, 8′	64.8	2.67	7′	123.9	5.66	4′, 7′
**7′**	69.3	-		67.6	-		70.8	-	
**8′**	30.5	1.17	6′, 7′, 8′	26.8	1.07	7′, 8′	29.8	1.34	5′,7′, 8′
**8′-Me**	30.5	1.17	6′, 7′, 8′	30.5	1.17	8′-Me, 7′	29.8	1.36	5′,7′, 8′
**8′-OH**	-	4.49	6′, 7′, 8′	-	n. d		-	4.18	7′

**Table 3:** Minimal inhibitory and bactericidal concentration (MIC and MBC) of ethanolic leaf extract, hexane fraction, and 11 isolated compounds of *X. stuhlmannii*.

Microbial Organisms										
Test Substance	Parameters	PA	SA	EC	KP	SF	SD	Stm	St	Se
**Leaf extract**	MIC	250	>500	>500	>500	>500	>500	>500	>500	250
	MBC	500	ND	ND	ND	ND	ND	ND	ND	500
	MBC/MIC	2	ND	ND	ND	ND	ND	ND	ND	2
**Hexane fraction**	MIC	>500	>500	>500	>500	>500	125	>500	>500	>500
	MBC	ND	ND	ND	ND	ND	250	ND	ND	ND
	MBC/MIC	ND	ND	ND	ND	ND	2	ND	ND	ND
**12**	MIC	>125	125	62.5	>125	>125	>125	125	>125	>125
	MBC	ND	250	250	ND	ND	ND	250	ND	ND
	MBC/MIC	ND	2	4	ND	ND	ND	2	ND	ND
**3**	MIC	>125	>125	>125	>125	125	125	>125	62.5	>125
	MBC	ND	ND	ND	ND	250	250	ND	250	ND
	MBC/MIC	ND	ND	ND	ND	2	2	ND	4	ND
**10**	MIC	>125	>125	>125	>125	>125	>125	125	>125	>125
	MBC	ND	ND	ND	ND	ND	ND	250	ND	ND
	MBC/MIC	ND	ND	ND	ND	ND	ND	2	ND	ND
**6**	MIC	>125	>125	>125	125	>125	>125	>125	>125	>125
	MBC	ND	ND	ND	250	ND	ND	ND	ND	ND
	MBC/MIC	ND	ND	ND	2	ND	ND	ND	ND	ND
**8**	MIC	>125	62.5	>125	>125	>125	>125	>125	125	>125
	MBC	ND	250	ND	ND	ND	ND	ND	250	ND
	MBC/MIC	ND	4	ND	ND	ND	ND	ND	2	ND
**2**	MIC	>125	>125	>125	>125	>125	125	125	125	>125
	MBC	ND	ND	ND	ND	ND	250	250	250	ND
	MBC/MIC	ND	ND	ND	ND	ND	2	2	2	ND
**13**	MIC	>125	>125	>125	125	125	>125	>125	>125	>125
	MBC	ND	ND	ND	250	250	ND	ND	ND	ND
	MBC/MIC	ND	ND	ND	2	2	ND	ND	ND	ND
**11**	MIC	>125	62.5	125	62.5	>125	>125	>125	>125	>125
	MBC	ND	250	250	250	ND	ND	ND	ND	ND
	MBC/MIC	ND	4	2	4	ND	ND	ND	ND	ND
**Amoxicillin**	MIC		0.5							
	MBC		1							
	MBC/MIC		2							
**Cipro**	MIC	1		0.25	0.5	0.25	0.5	0.5	0.5	0.5
	MBC	4		1	2	2	1	2	1	2
	MBC/MIC	4		4	4	0.125	2	4	2	4

Values are expressed (µg/mL) as means ± SEM.; PA: *Pseudomonas aeruginosa* (ATCC01); SA: *Staphylococcus aureus* (ATCC25922); EC_s_: *Escherichia coli* (ATCC10536); KP: *Klessiella pneumonae* (ATCC13883); SF: *Shigella flexineri*; SD: *Shigella dysenteria*; Stm: *Salmonella typhimurium*; ST: *Salmonella typhi* (ATCC6539); SE: *Salmonella enteritidis*. ND: not determined. MIC = Minimum inhibitory concentration; MBC = Minimum bactericidal concentration; MBC/MIC; The ratio MBC/MIC determine the bactericidal (MBC/MIC ≤ 4) or bacteriostatic (MBC/MIC > 4) effects of extracts. Cipro: Ciprofloxacin, Amox: Amoxicillin.

**Table 4:** Minimal inhibitory and fungicidal concentration (MIC and MFC) of ethanolic leaf extract, hexane fraction, and 11 isolated compounds of *X. stuhlmannii*.

Yeasts Strains					
Tested Substances	Parameters	CA	CK	CP	CN
**Leaf extract**	MIC	>500	>500	>500	>500
	MFC	ND	ND	ND	ND
	MFC/MIC	ND	ND	ND	ND
**12**	MIC	>125	62.5	125	125
	MFC	ND	250	250	250
	MFC/MIC	ND	4	2	2
**3**	MIC	125	>125	62.5	125
	MFC	250	ND	250	250
	MFC/MIC	2	ND	4	2
**6**	MIC	>125	>125	125	125
	MFC	ND	ND	250	250
	MFC/MIC	ND	ND	2	2
**8**	MIC	62.5	125	>125	>125
	MFC	250	250	ND	ND
	MFC/MIC	4	2	ND	ND
**2**	MIC	>125	125	>125	125
	MFC	ND	250	ND	250
	MFC/MIC	ND	2	ND	2
**Hexane fraction**	MIC	>500	>500	125	>500
	MFC	ND	ND	250	ND
	MFC/MIC	ND	ND	2	ND
**13**	MIC	>125	125	>125	>125
	MFC	ND	250	ND	ND
	MFC/MIC	ND	2	ND	ND
**11**	MIC	>125	125	125	>125
	MFC	ND	250	250	ND
	MFC/MIC	ND	2	2	ND
**Nysta**	MIC	0.5	0.25	1	0.25
	MFC	2	1	2	1
	MFC/MIC	4	0.25	2	0.25

Values are expressed (in µg/mL) as means ± SEM. Minimum inhibitory concentrations (MIC); Minimum fungicidal concentrations (MFC). CA: *Candida albicans,* CK: *Candida krusei*, CP: *Candida parasilosis*, CN = *Cryptococcus neoformans*; Nysta: Nystatin.

**Table 5:** Radical scavenging activity (DPPH and ABTS) and ferric reducing power (FRAP) of ethanolic leaf extract and isolates.

Tests Materials	DPPH (RSa_50_ (µg/mL))	ABTS (RSa_50_ (µg/mL))	FRAP (RC_50_ (µg/mL))
**Leaf extract**	16.36 ± 0.77 ^b^	13.44 ± 0.82 ^b^	39.85 ± 0.96 ^d^
**12**	17.93 ± 0.82 ^b^	35.20 ± 0.88 ^d^	24.09 ± 0.41 ^b^
**6**	14.97 ± 0.86 ^b^	22.31 ± 1.20 ^b^	25.31 ± 0.52 ^d^
**5**	24.29 ± 2.03 ^c^	17.41 ± 1.00 ^b^	15.07 ± 0.41 ^b^
**7**	19.10 ± 1.50 ^c^	14.41 ± 0.24 ^b^	19.50 ± 4.50 ^b^
**Vitamin C**	8.92 ± 0.06 ^a^	2.71 ± 0.08 ^a^	13.94 ± 0.07 ^a^

Values are expressed as mean ± SEM. Along each column, values with the same letter superscripts are not significantly different, Bonferroni test (*p* > 0.05). RS_a50_ of DPPH and ABTS in µg/mL; RC_50_ of FRAP in µg/mL.

## Data Availability

The supporting information can be found in the Supplementary Materials.

## Supplementary Materials

The following supporting information can be downloaded at: https://www.mdpi.com/article/10.3390/molecules28062846/s1, Figure S1–S63: HRESIMS, 1D, and 2D NMR spectra of **1**–**5**, HRESIMS, and 1D NMR spectra of **6**–**14,** UPLC-ESI-TOF-MS, and preparative HPLC parameters.

## Sample Availability

Samples of the compounds are not available from the authors.
