# Supplementary material for: Rotenoids and Isoflavones from Xeroderris stuhlmannii (Taub.) Mendonça & E.P. Souza and Their Biological Activities
Source: Molecules. 2023 Mar 21;28(6):2846. doi: 10.3390/molecules28062846 (PMC10059994; doi:10.3390/molecules28062846)
Supplement: Supplementary file 1 [file molecules-28-02846-s001.zip › molecules-2271320-supplementary.pdf]

Supplementary Materials

Rotenoids and Isoflavones from *Xeroderris stuhlmannii* (Taub.) Mendonça &E.P. Souza and Their Biological Activities

Livie Blondèle Kenou Mekuete [1,2], Willifred Dongmo Tékapi Tsopgni [2,3], Augustine Kuinze Nkojap [4], Jacquy Joyce Wanche Kojom [4], Timo D. Stark [3,*], Yannick Fouokeng [2], Alain Bertrand Dongmo [4], Léon Tapondjou Azeufack [1] and Anatole Guy Blaise Azebaze [2]

[1] Research Unit of Environmental and Applied Chemistry, Faculty of Science, University of Dschang, Dschang P.O. Box 67, Cameroon; kenoulivie14@gmail.com (L.B.K.M.); tapondjou2001@yahoo.fr (L.T.A.)

[2] Department of Chemistry, Faculty of Sciences, University of Douala, 24157 Douala, Cameroon; willifred2kpi@yahoo.fr (W.D.T.T.), yfouokeng@gmail.com (Y.F.), azebaze@gmail.com (A.G.B.A.)

[3] Lehrstuhl für Lebensmittelchemie und Molekulare Sensorik, Technische Universität München, 85354 Freising, Germany

[4] Department of Animal Biology and Physiology, Faculty of Sciences, University of Douala, Douala 24517 Cameroon; augustine_kuinze@yahoo.fr (A.K.N.); kojomjoyce@yahoo.fr (J.J.W.K.); alainberd@yahoo.fr (A.B.D.)

* Correspondence: timo.stark@tum.de; Tel.: +00-(49)-8161-712911

Contents	Pages
**Figure S1.** ^{1}H NMR spectrum of **1** in $CDCl_3$.	4
**Figure S2**. ^{13}C NMR spectrum of **1** in $CDCl_3$.	5
**Figure S3.** DEPT 135 spectrum of **1** in $CDCl_3$.	5
**Figure S4.** HSQC spectrum of **1** in $CDCl_3$.	6
**Figure S5.** HMBC spectrum of **1** in $CDCl_3$.	6
**Figure S6.** HRESIMS (1) spectrum of **1**.	7
**Figure S7.** ^{1}H NMR (1) spectrum of **2** in $CDCl_3$.	7
**Figure 8.** ^{1}H NMR (2) spectrum of **2** in $CDCl_3$.	8
**Figure 9.** ^{13}C NMR spectrum of **2** in $CDCl_3$.	8
**Figure 10.** DEPT 135 spectrum of **2**.	9
**Figure 11.** COSY spectrum of **2** in $CDCl_3$.	9
**Figure 12.** HSQC spectrum of **2** in $CDCl_3$.	10
**Figure 13.** HMBC (**1**) spectrum of **2** in $CDCl_3$.	10
**Figure 14.** HMBC (2) spectrum of 2 in $CDCl_3$.	11
**Figure 15**. HRESIMS spectrum of **2**.	11
**Figure 16.** ^{1}H NMR (1) spectrum of **3** in DMSO.	12
**Figure 17.** ^{1}H NMR (2) spectrum of **3** in DMSO.	12
**Figure 18.** ^{13}C NMR spectrum of **3** in DMSO.	13
**Figure 19.** COSY spectrum of **3** in DMSO.	13
**Figure 20**. HSQC spectrum of **3** in DMSO.	14
**Figure 21.** HMBC spectrum of **3** in DMSO.	14
**Figure 22.** HRESIMS spectrum of **3**.	15
**Figure 23.** ^{1}H NMR spectrum (1) of **4** in DMSO	15
**Figure 24.** ^{1}H NMR spectrum (2) of **4** in DMSO	16
**Figure 25.** ^{13}C NMR spectrum of **4** in DMSO	17
**Figure 26.** COSY spectrum of **4** in DMSO	18
**Figure 27.** HSQC spectrum of **4** in DMSO	18
**Figure 28.** HMBC spectrum of **4** in DMSO	19
**Figure 29.** HRESIMS spectrum of **4**.	19
**Figure 30.** ^{1}H NMR spectrum (1) of **5** in $CDCl_3$.	20
**Figure 31.** ^{1}H NMR spectrum (2) of **5** in $CDCl_3$.	20
**Figure 32.** ^{13}C NMR spectrum of **5** in $CDCl_3$.	21
**Figure 33.** DEPT 135 spectrum of **5** in $CDCl_3$.	22
**Figure 34.** COSY spectrum of **5** in $CDCl_3$.	22
**Figure 35.** HSQC spectrum of **5** in $CDCl_3$.	23
**Figure 36.** HMBC spectrum of **5** in $CDCl_3$.	23
**Figure 37.** HRESIMS spectrum of **5** in $CDCL_3$.	24
**Figure 38.** ^{1}H NMR spectrum (500MHz, $CDCl_3$) of compound **6.**	25
**Figure 39.** ^{13}C NMR spectrum (125 MHz, $CDCL_3$) of the compound **6.**	25
**Figure 40.** HRESIMS spectrum of **6.**	26
**Figure 41.** ^{1}H NMR spectrum (500MHz, CDCl3) of compound **7.**	26
**Figure 42.** ^{13}C NMR spectrum (125MHz, $CDCl_3$) of compound **7.**	27
**Figure 43.** HRESIMS spectrum of **7.**	27
**Figure 44.** ^{1}H NMR (500 MHz, $CDCl_3$) spectrum of **8.**	28
**Figure 45**. ^{13}C NMR (125MHz, $CDCl_3$) spectrum of **8.**	28
**Figure 46**. HRESIMS spectrum of **8.**	29
**Figure 47**. ^{1}H NMR (500MHz, $CDCl_3$) spectrum of **9.**	29
**Figure 48.** ^{13}C NMR (125MHz, $CDCl_3$) spectrum of **9**.	30
**Figure 49.** HRESIMS spectrum of **9**.	30

Figure 50. ^{1}H NMR (500MHz, MeOD) spectrum of **10.** 31
Figure 51. ^{13}C NMR (125MHz, MeOD) spectrum of **10.** 31
Figure 52. HRESIMS spectrum of 10. 32
Figure 53. ^{1}H NMR (500 MHz, $CDCl_3$) spectrum of **11.** 32
Figure 54. 135 DEPT (125MHz, $CDCl_3$) spectrum of **11.** 33
Figure 55. ^{13}C NMR (125MHz, $CDCl_3$) spectrum of **11**. 33
Figure 56. ^{1}H NMR (500 MHz, MeOD) spectrum of **12.** 34
Figure 57. ^{13}C NMR (125MHz, MeOD) spectrum of **12.** 34
Figure 58. HRESIMS spectrum of **12.** 35
Figure 59. ^{1}H NMR (500 MHz, $CDCl_3$) spectrum of **13.** 35
Figure 60. ^{1}H-^{1}H COSY (125MHz, $CDCl_3$) spectrum of **13.** 36
Figure 61. ^{1}H NMR (500 MHz, $CDCl_3$) spectrum of **14.** 36
Figure 62. 135 DEPT (125MHz, $CDCl_3$) spectrum of **14**. 37
Figure 63. HRESIMS spectrum of **14**. 37
UPLC-ESI-TOF-MS analysis........ 25
HPLC Parameters for the compound isolation........ 26

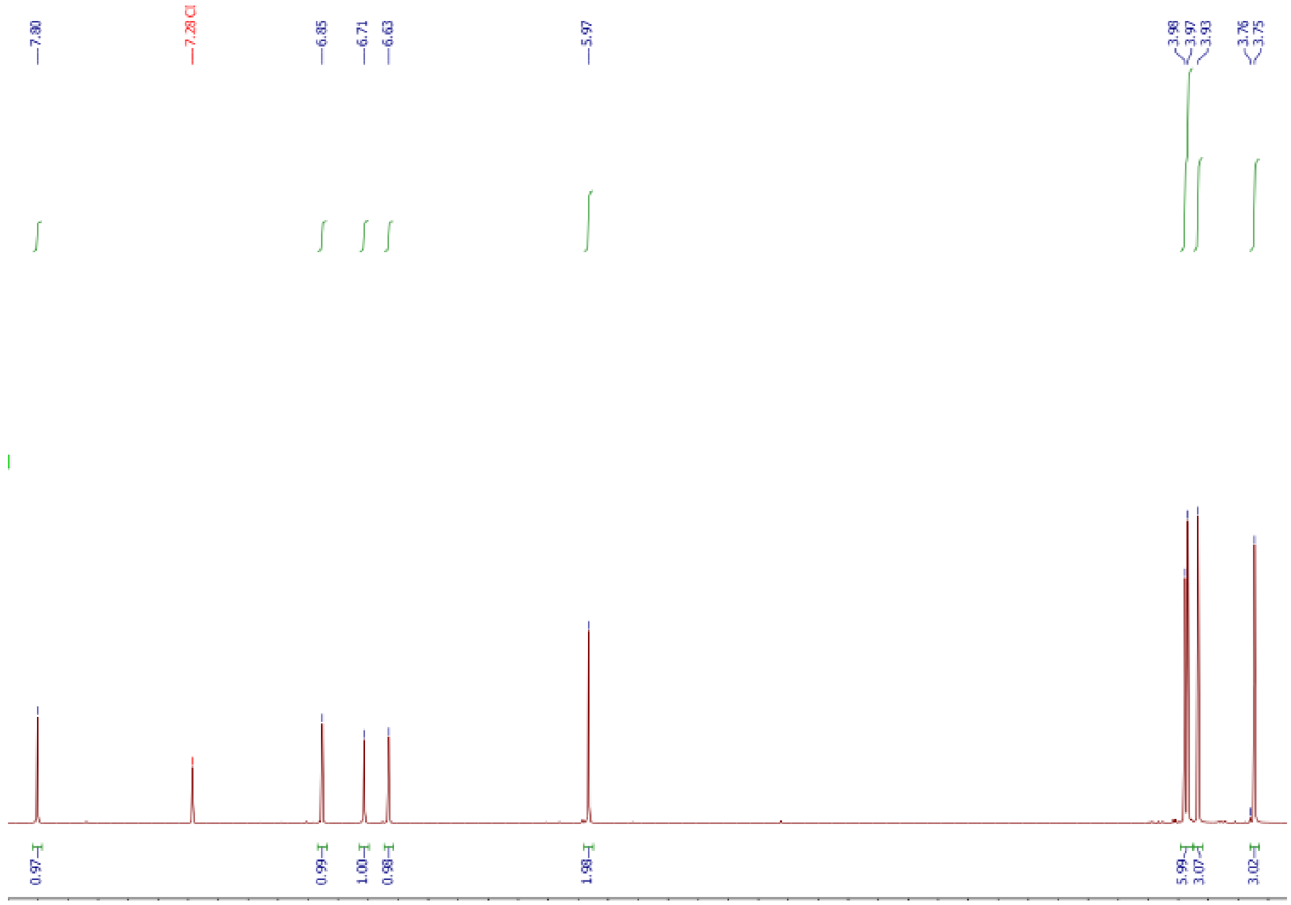

Figure S1. ^{1}H NMR spectrum of **1** in $CDCl_3$.

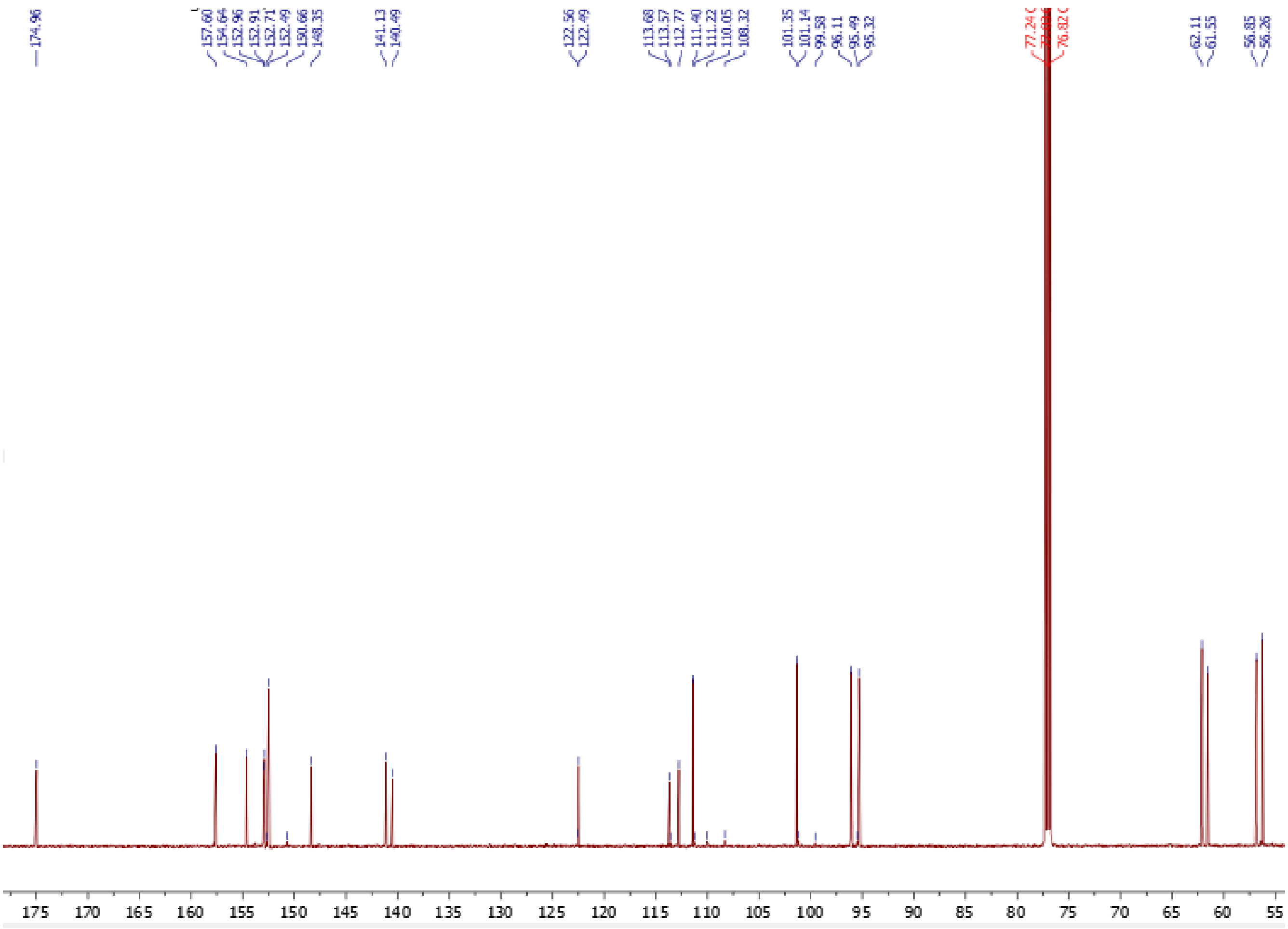

Figure S2. ^{13}C NMR spectrum of **1** in $CDCl_3$.

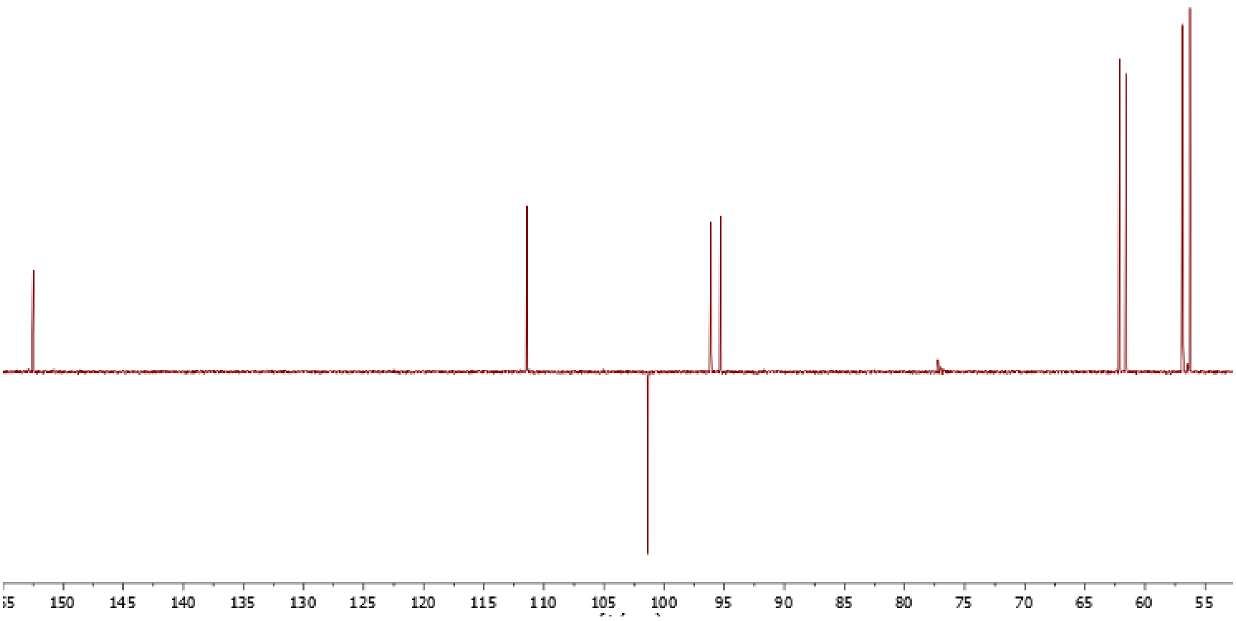

Figure S3. DEPT 135 spectrum of **1** in $CDCl_3$.

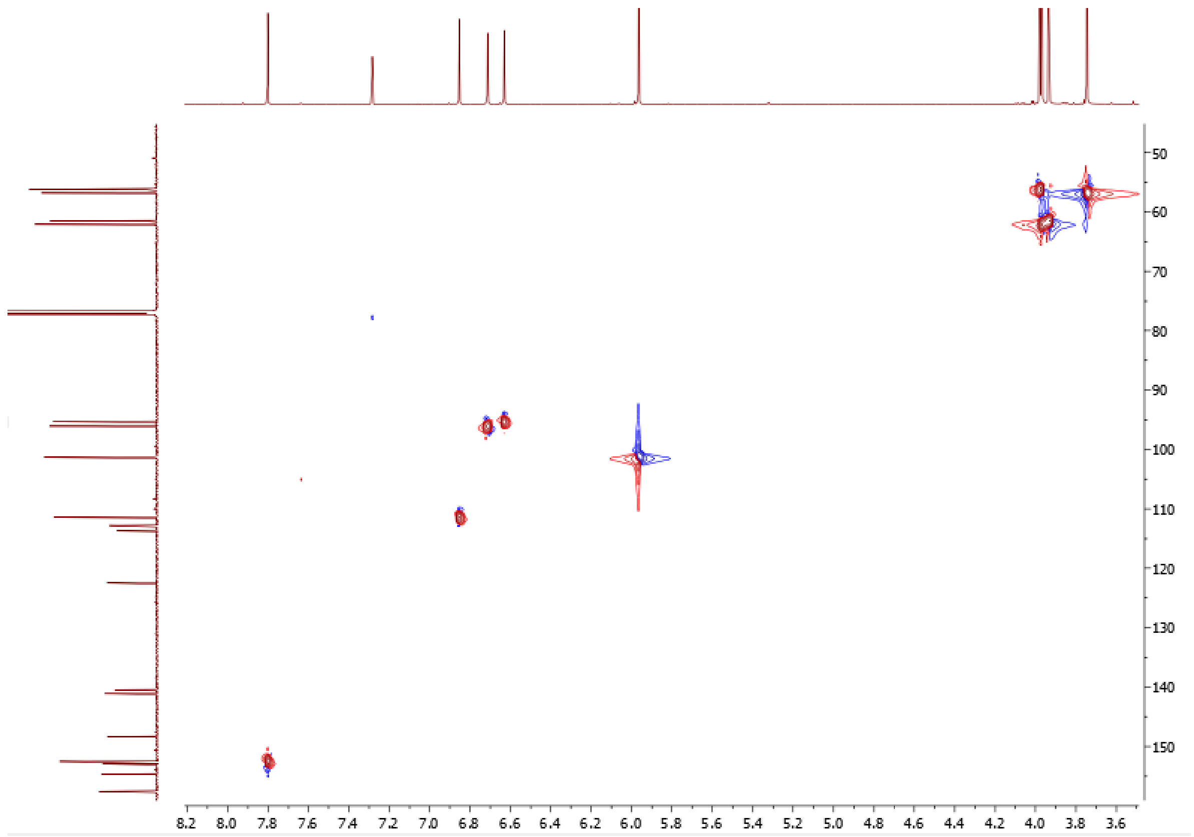

Figure S4. HSQC spectrum of **1** in $CDCl_3$.

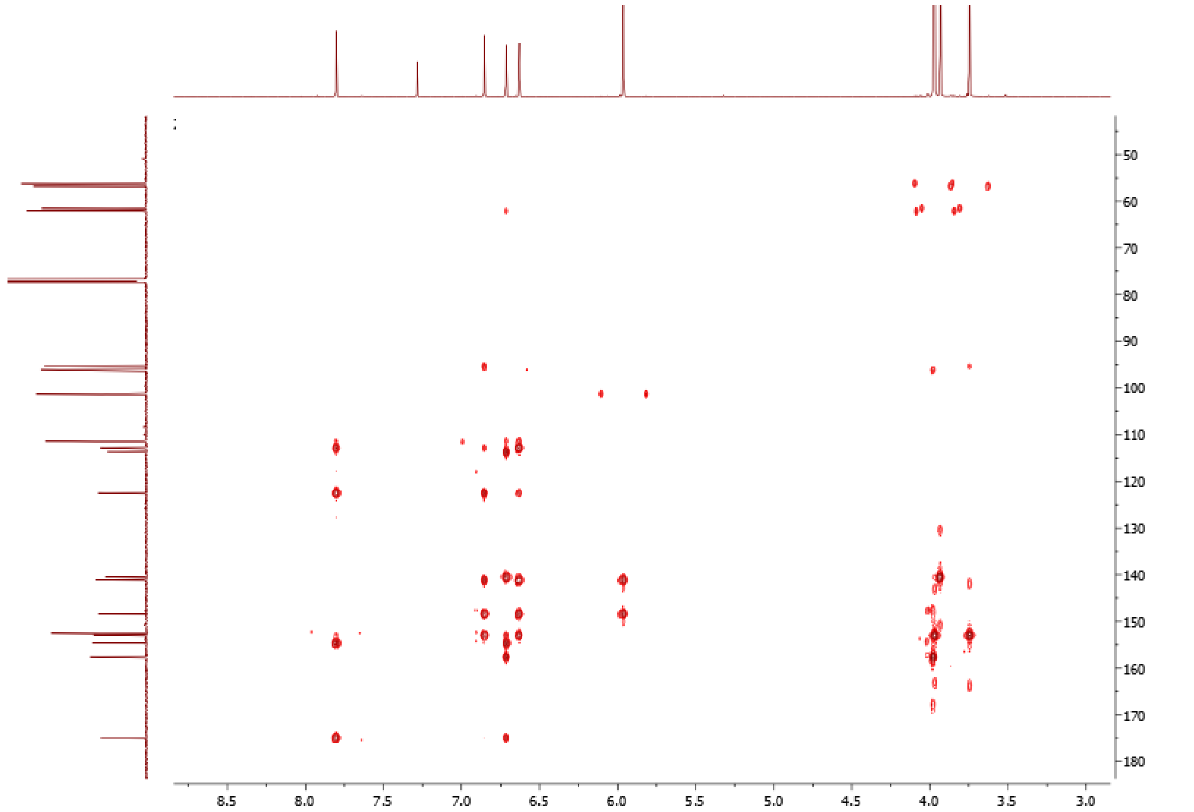

Figure S5. HMBC spectrum of **1** in $CDCl_3$.

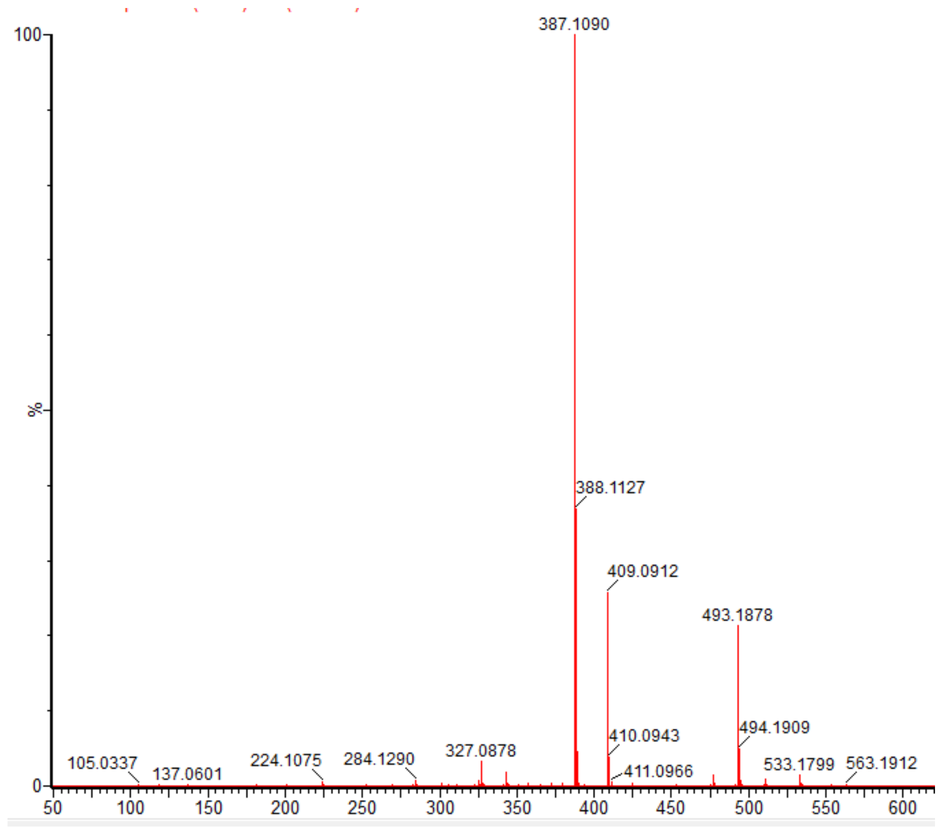

Figure S6. HRESIMS (1) spectrum of **1**.

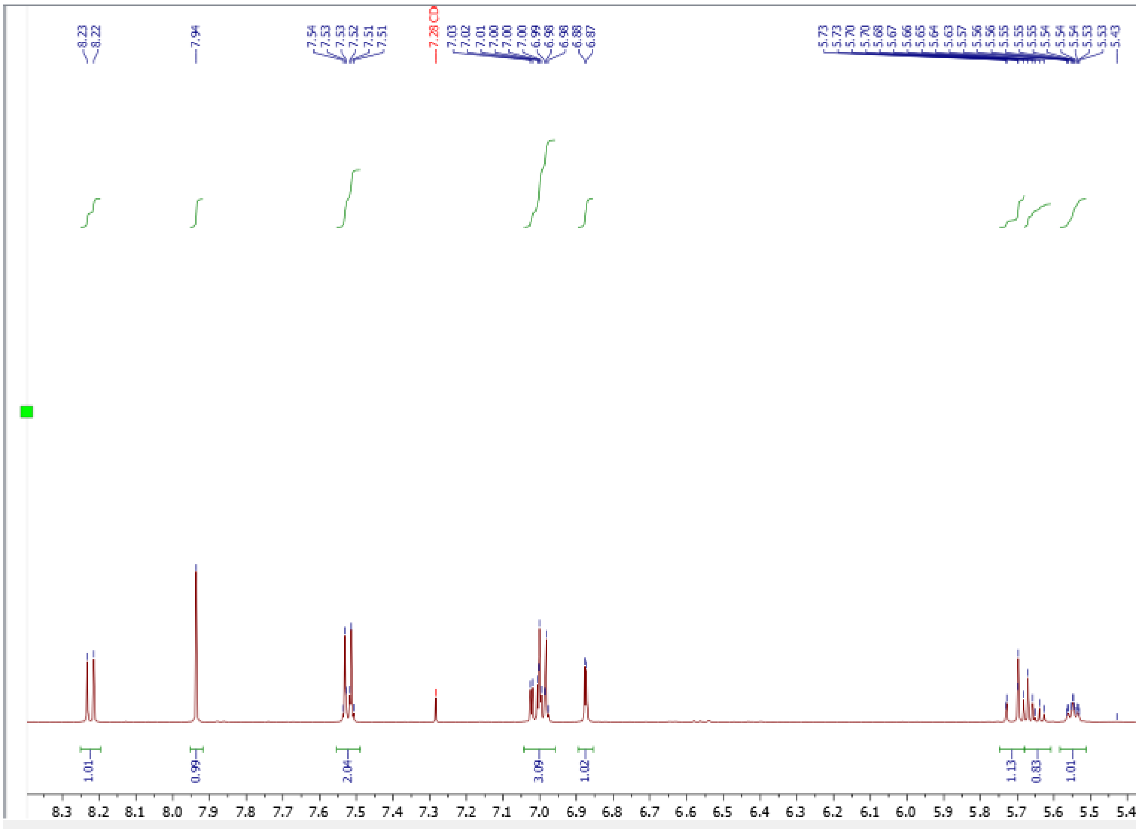

Figure S7. ^{1}H NMR (1) spectrum of **2** in $CDCl_3$.

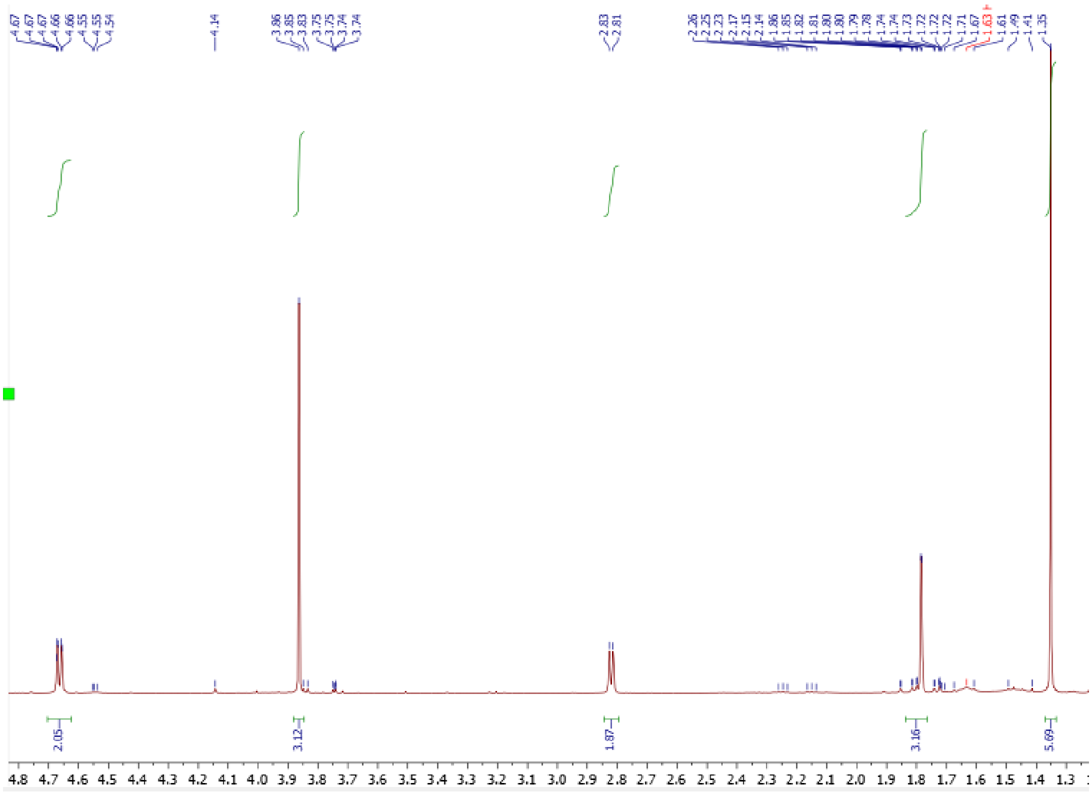

Figure S8. ^{1}H NMR (2) spectrum of **2** in $CDCl_3$.

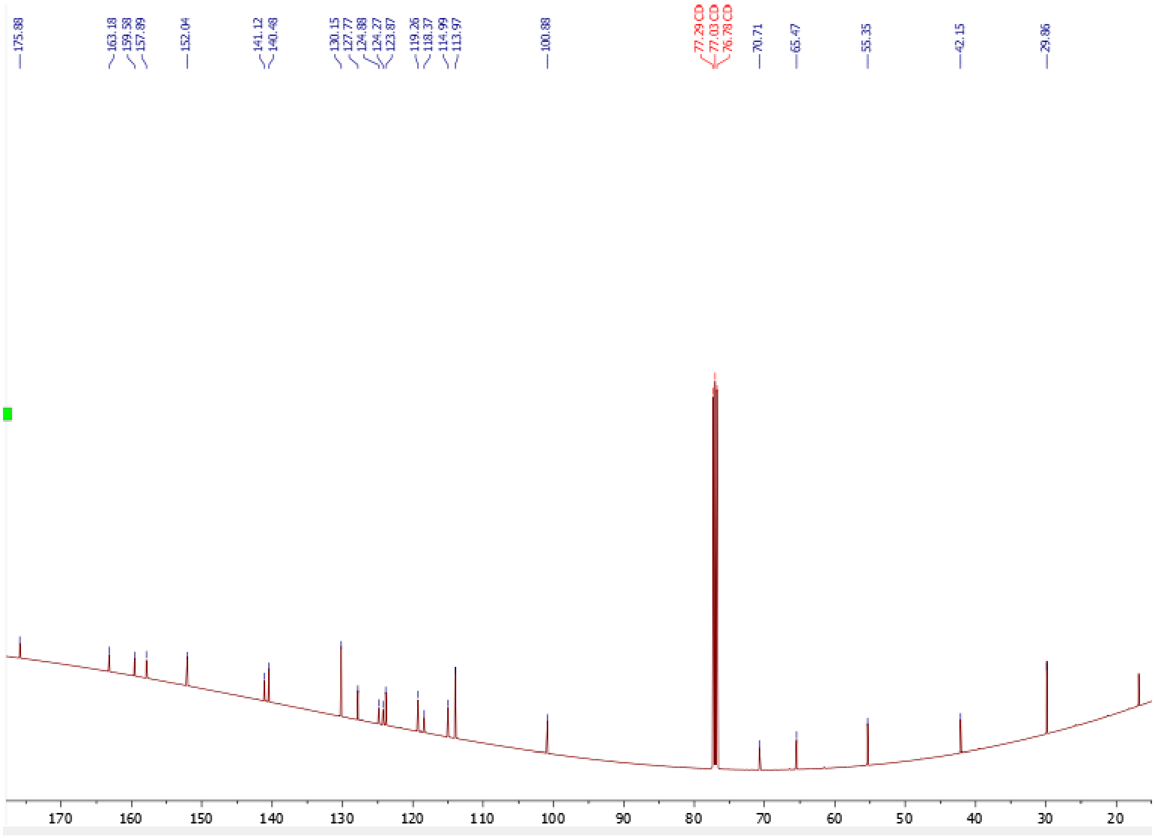

Figure S9. ^{13}C NMR spectrum of **2** in $CDCl_3$.

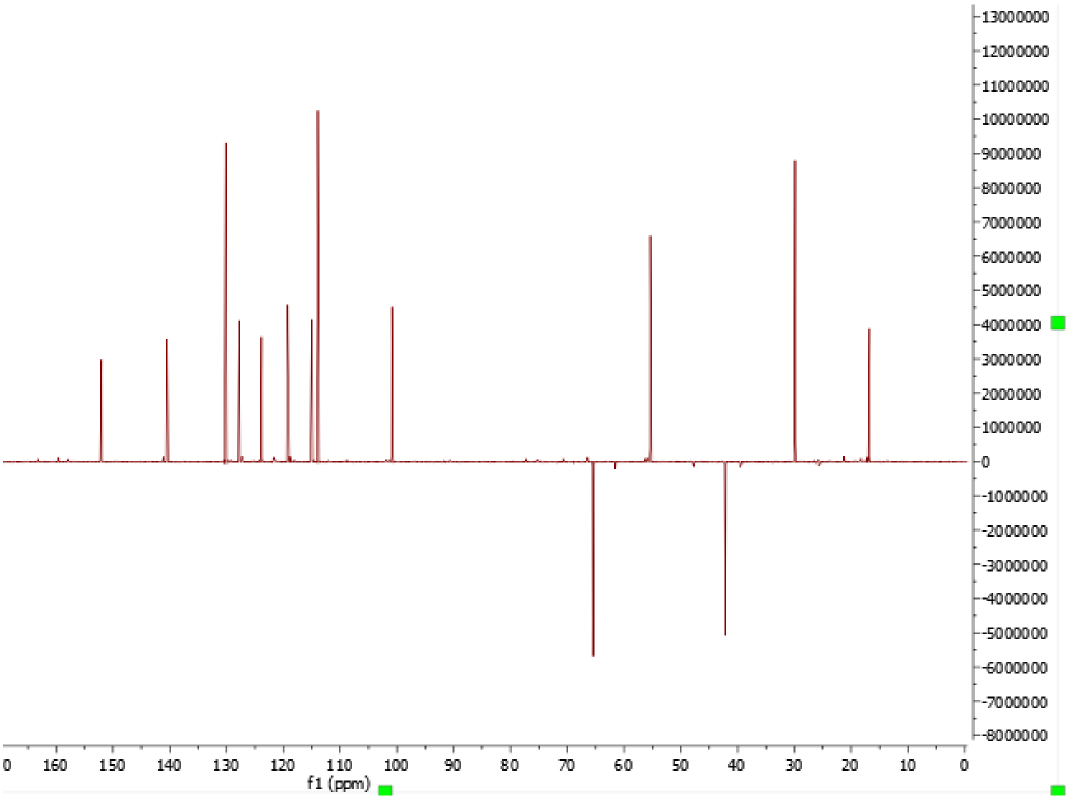

Figure S10. DEPT 135 spectrum of **2**.

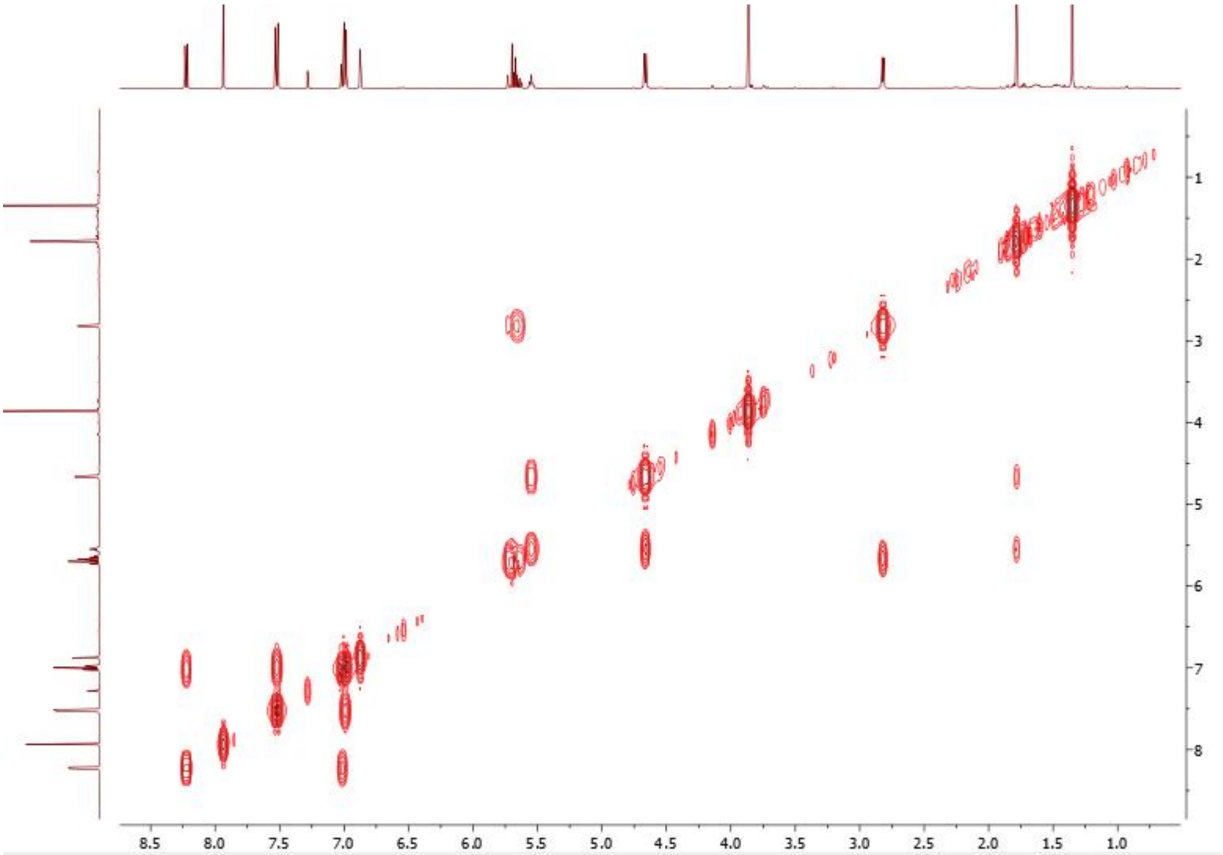

Figure S11. COSY spectrum of **2** in $CDCl_3$.

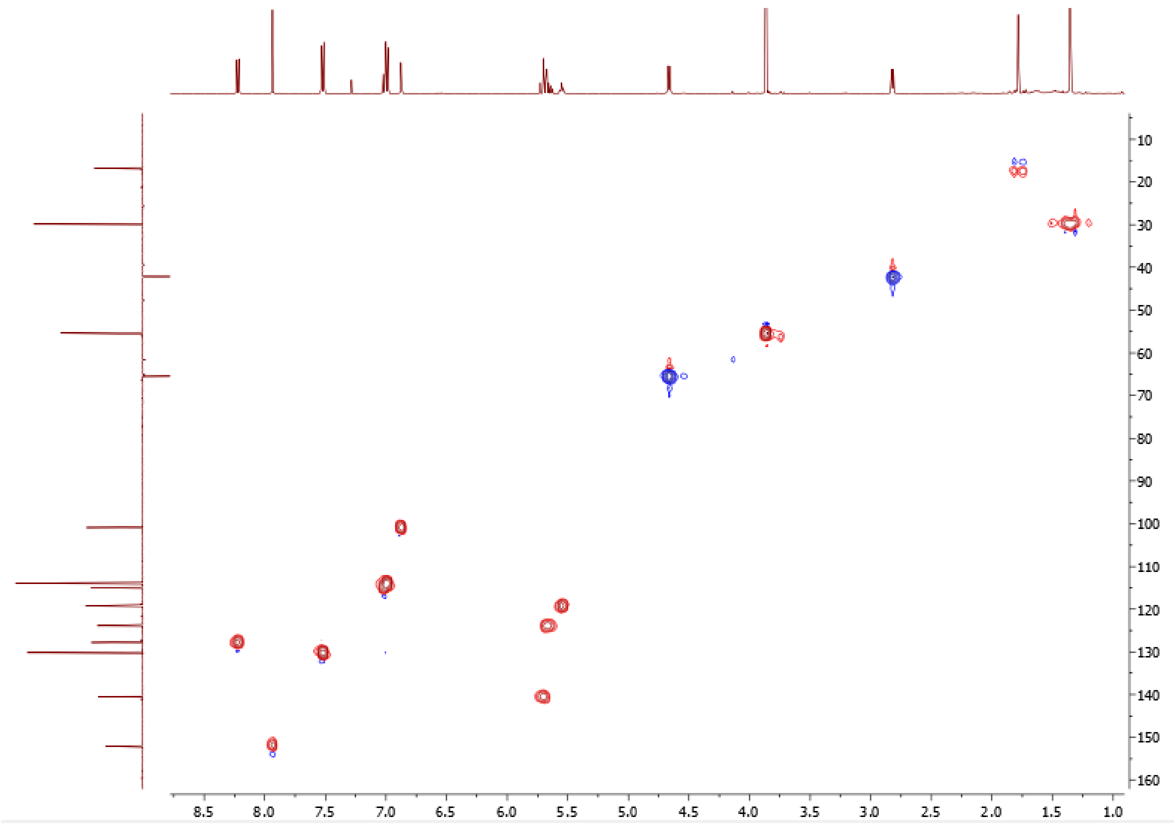

Figure S12. HSQC spectrum of **2** in $CDCl_3$.

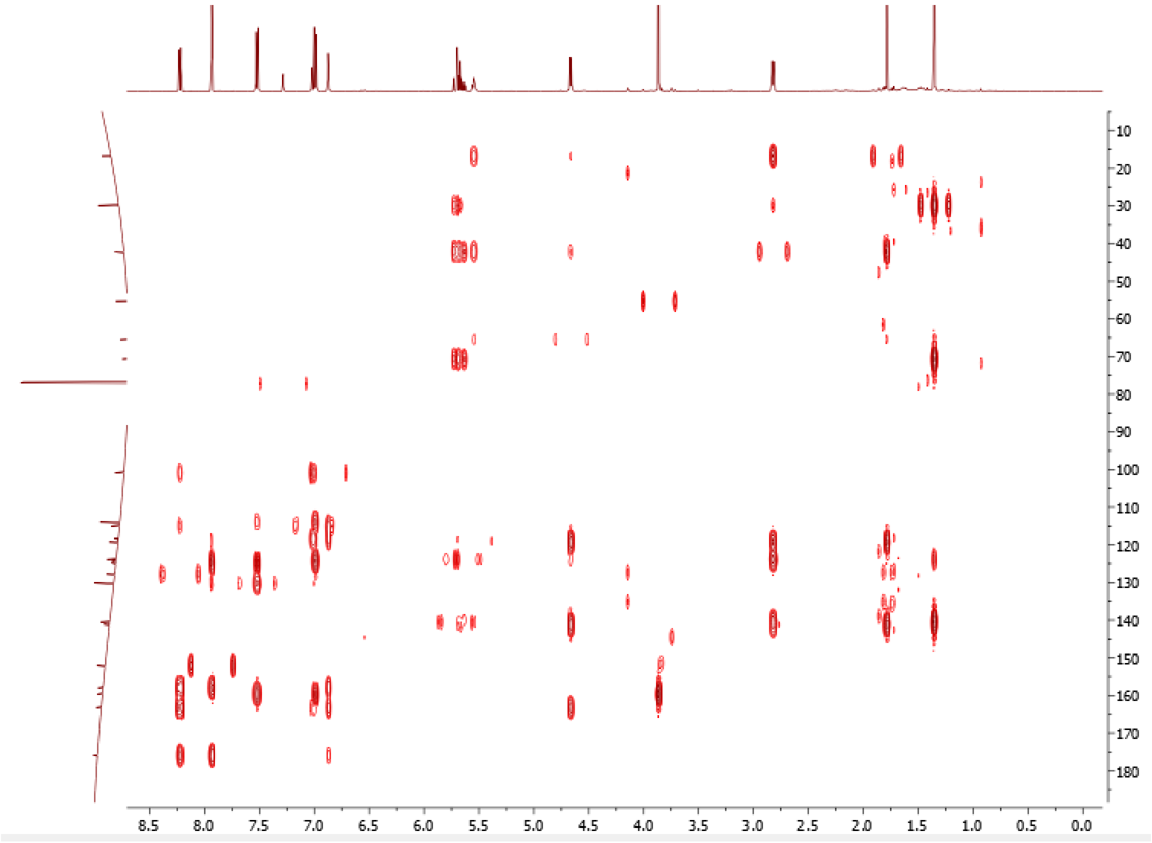

Figure S13. HMBC (**1**) spectrum of **2** in $CDCl_3$.

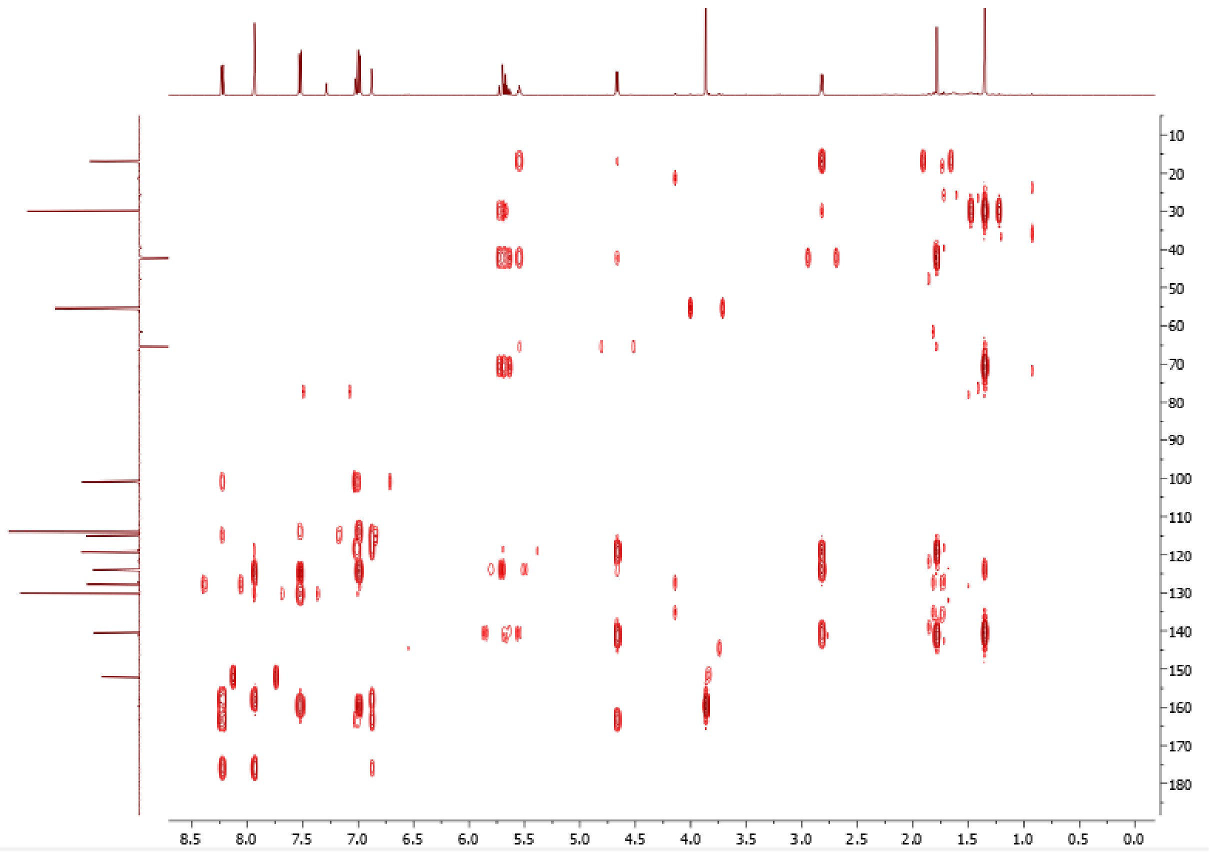

Figure S14. HMBC (2) spectrum of 2 in $CDCl_3$.

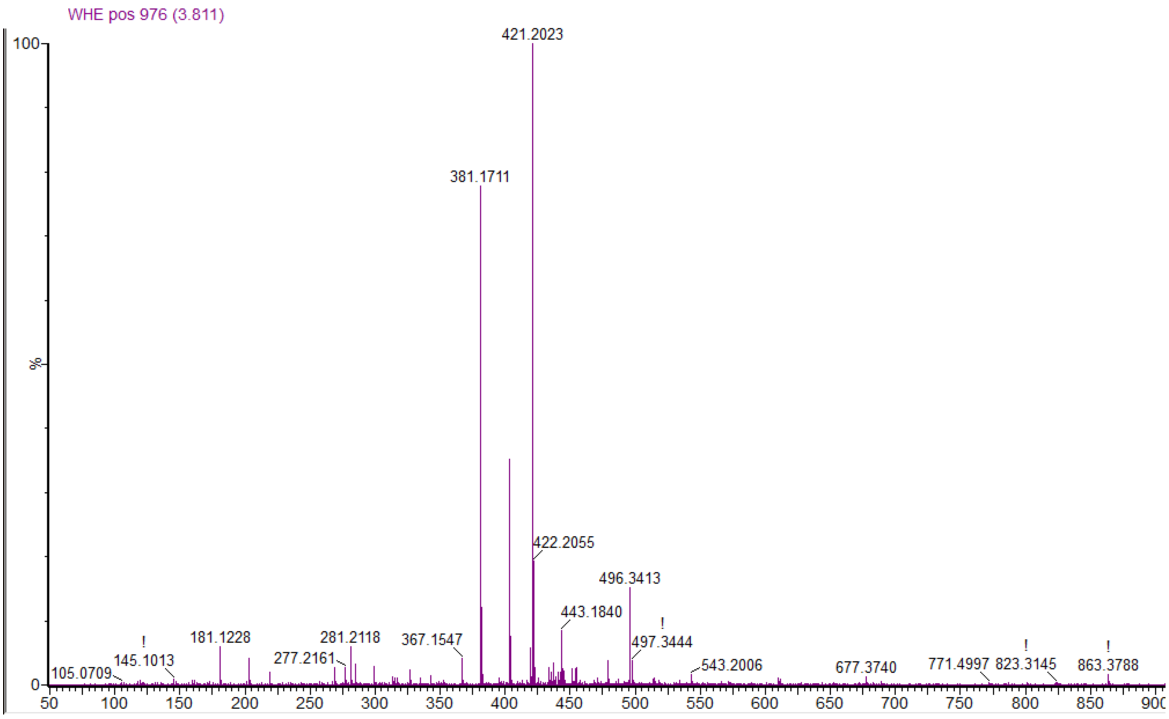

Figure S15. HRESIMS spectrum of **2**.

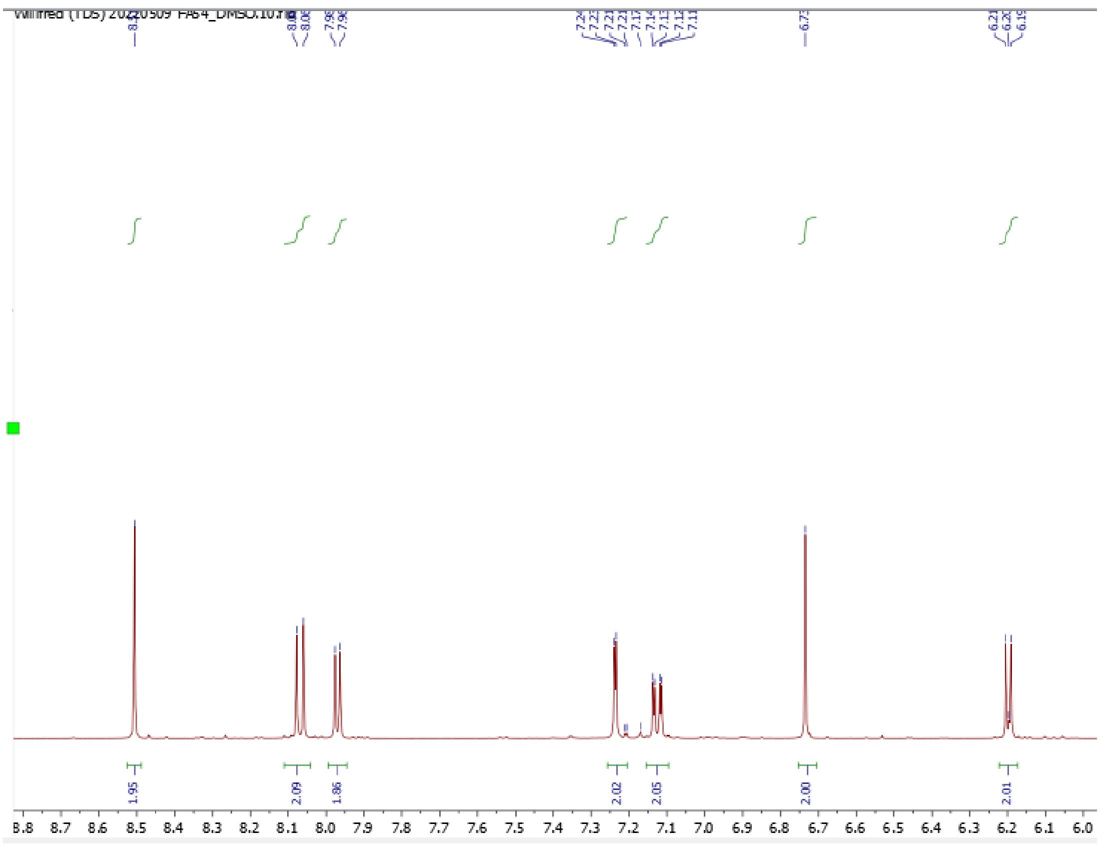

Figure S16. ^{1}H NMR (1) spectrum of **3** in DMSO.

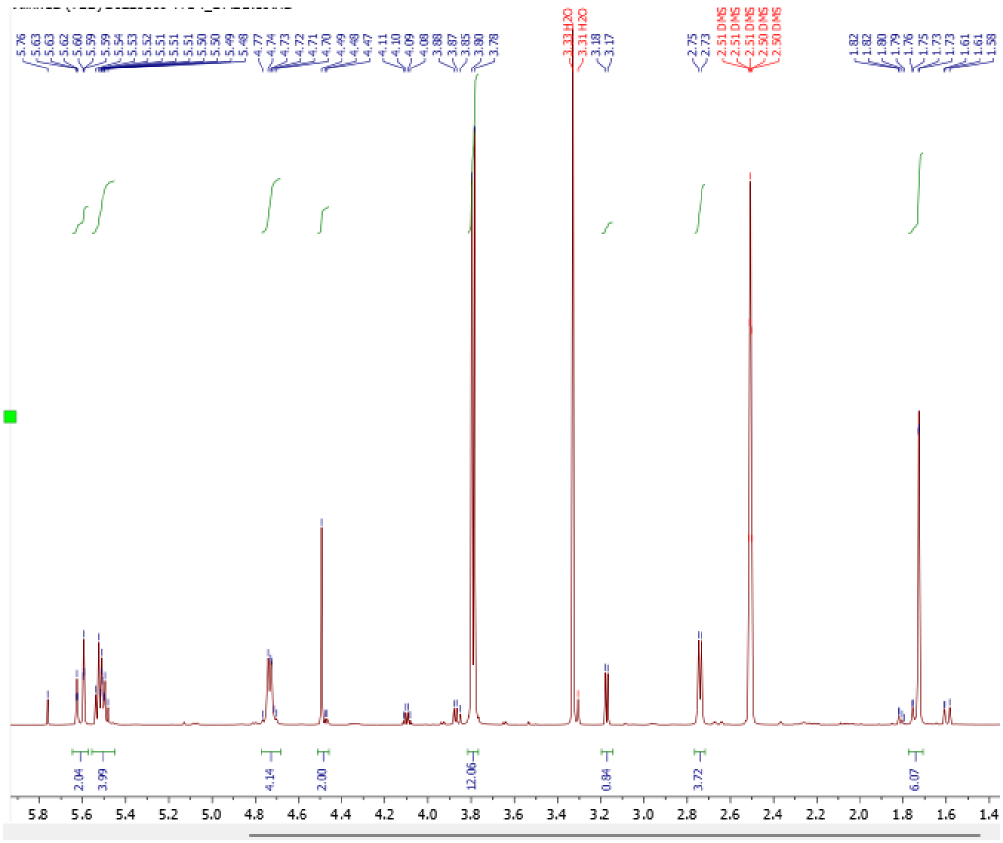

Figure S17. ^{1}H NMR (2) spectrum of **3** in DMSO.

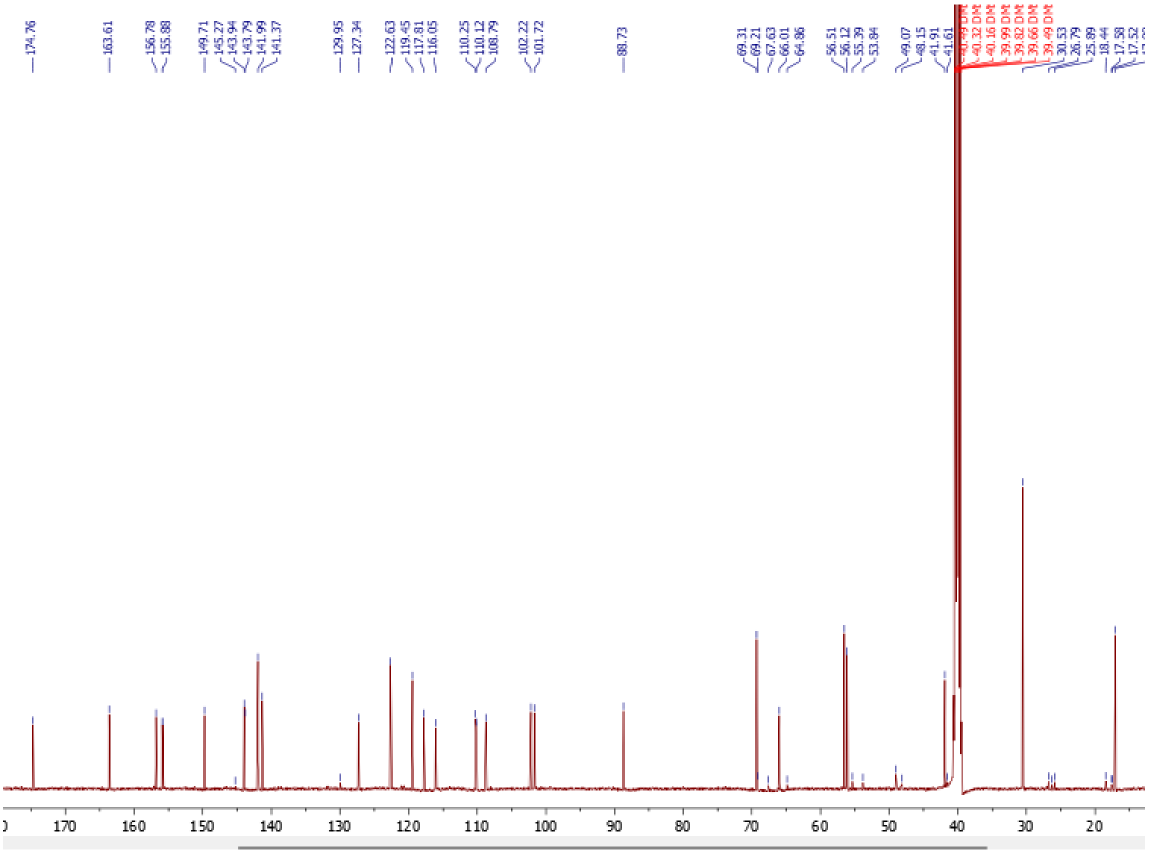

Figure S18. ^{13}C NMR spectrum of **3** in DMSO.

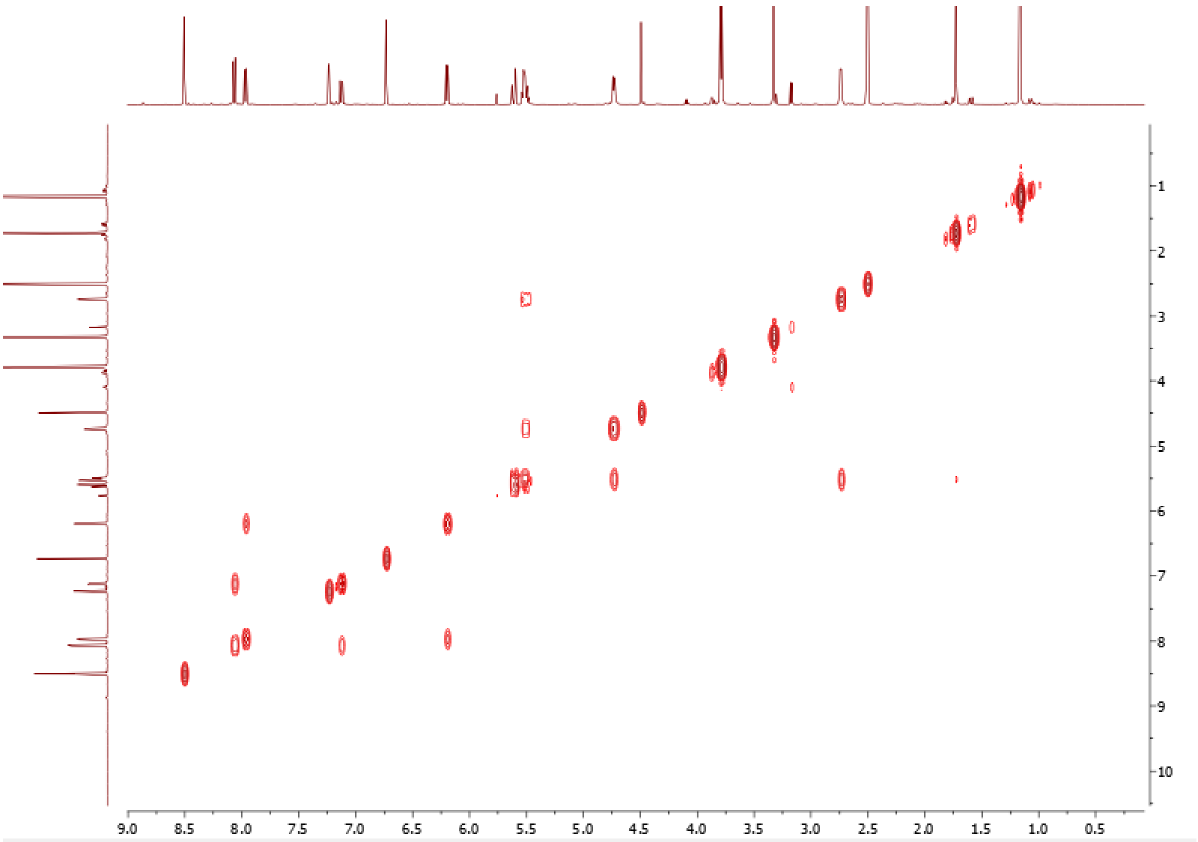

Figure S19. COSY spectrum of **3** in DMSO.

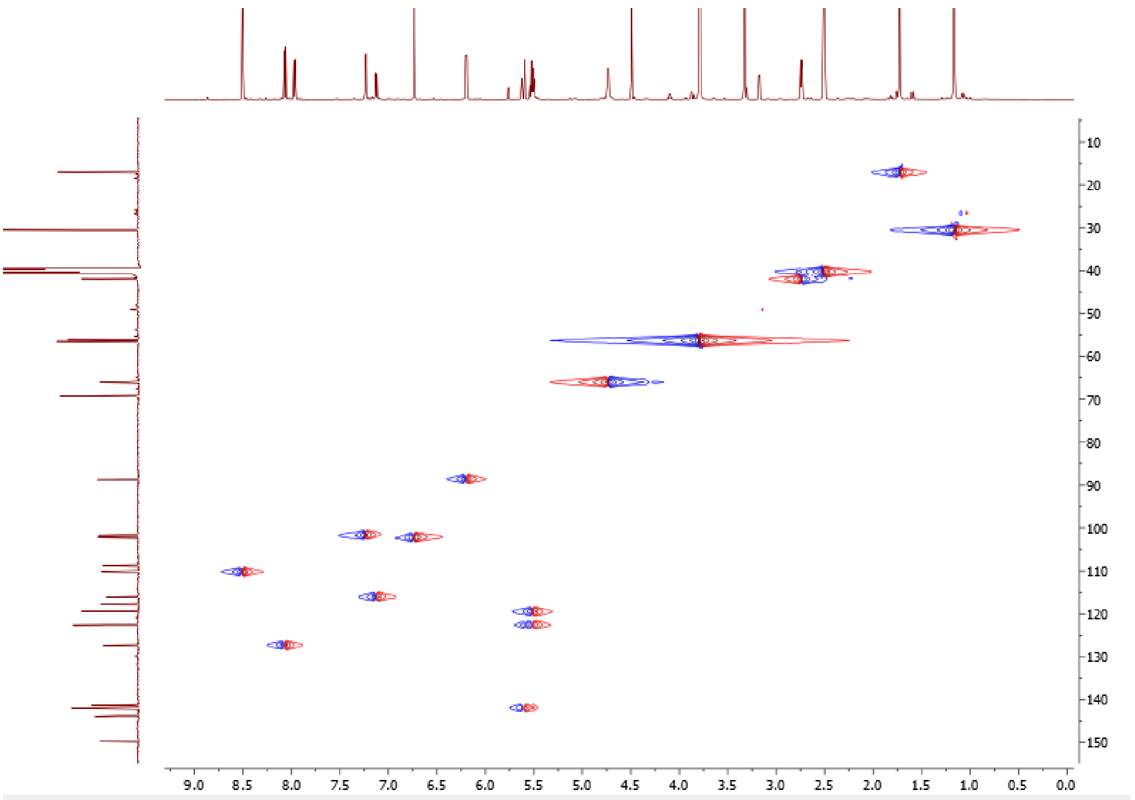

Figure S20. HSQC spectrum of **3** in DMSO.

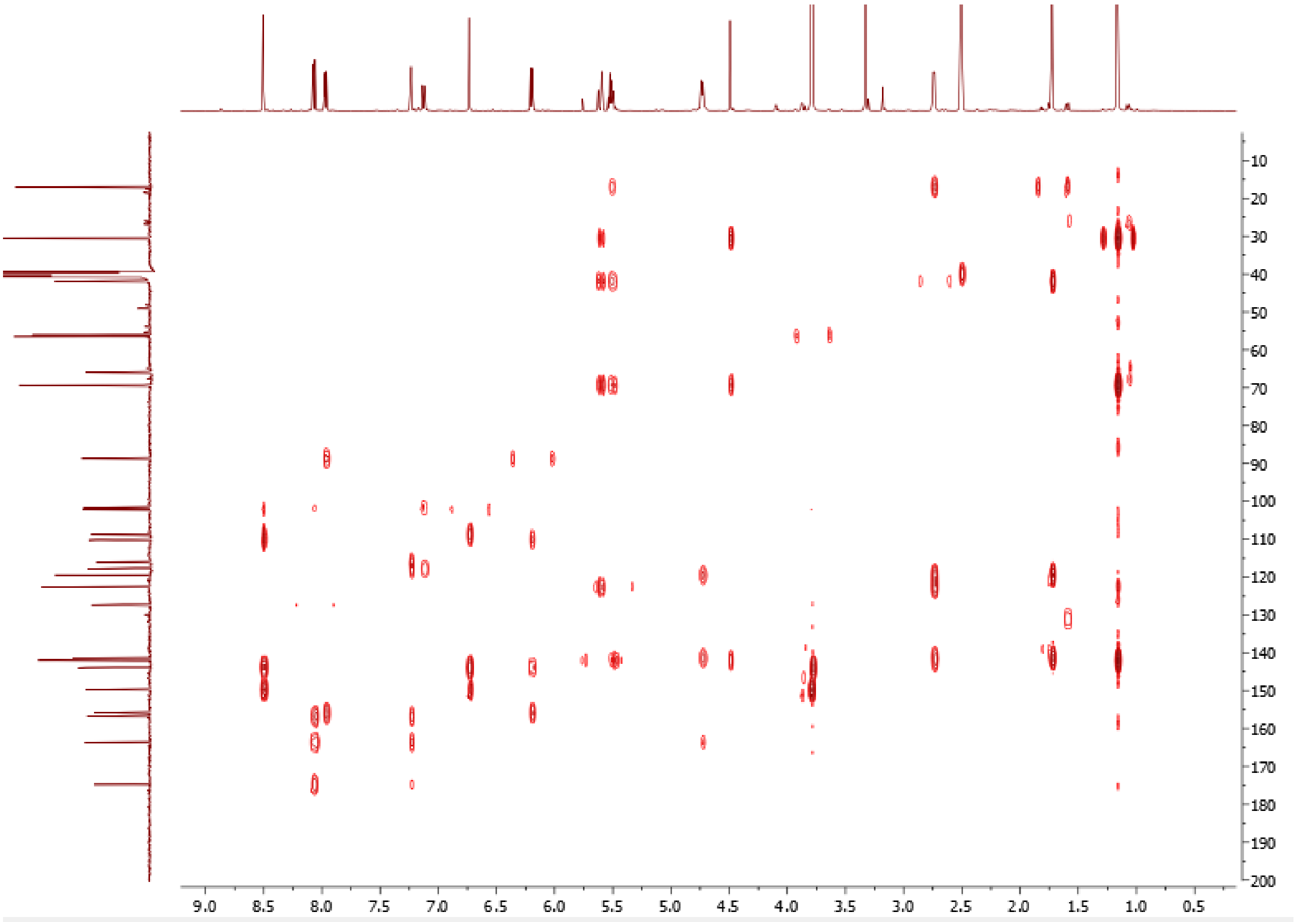

Figure S21. HMBC spectrum of **3** in DMSO.

Multiple Mass Analysis: 2 mass(es) processed
Tolerance = 5.0 mDa / DBE: min = -1.5, max = 50.0
Element prediction: Off
Number of isotope peaks used for i-FIT = 3
Monoisotopic Mass, Even Electron Ions
185 formula(e) evaluated with 3 results within limits (up to 50 best isotopic matches for each mass)
Elements Used:

Mass	RA	Calc. Mass	mDa	PPM	DBE	Formula	i-FIT	i-FIT Norm	Fit Conf %	C	H	O
343.0826	85.59	343.0818	0.8	2.3	11.5	C18 H15 O7	2388.7	n/a	n/a	18	15	7
477.1946	100.00	477.1913	3.3	6.9	14.5	C28 H29 O7	1814.7	0.111	89.45	28	29	7
		477.1972	-2.6	-5.4	5.5	C21 H33 O12	1816.8	2.249	10.55	21	33	12

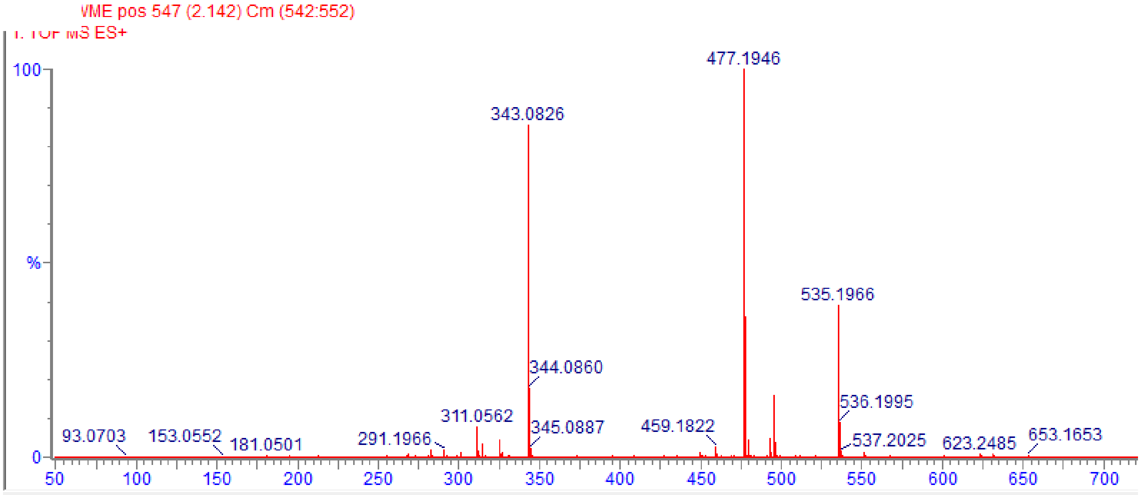

Figure S22. HRESIMS spectrum of **3**.

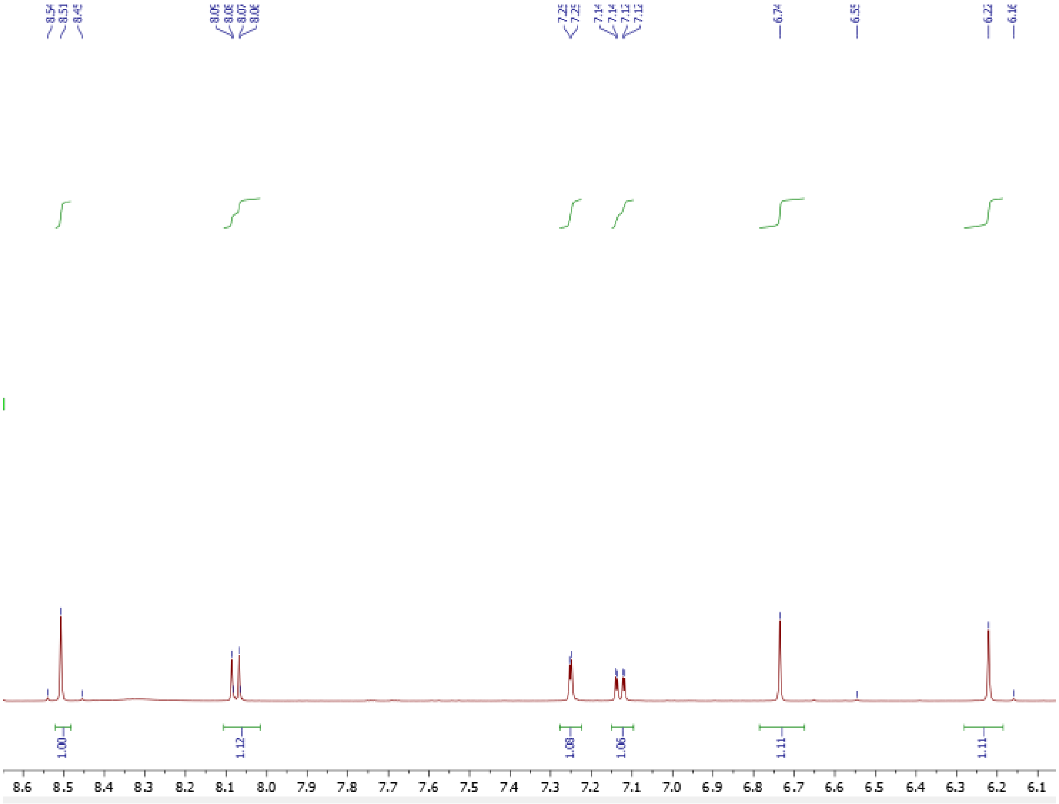

Figure S23. ^{1}H NMR spectrum (1) of **4** in DMSO

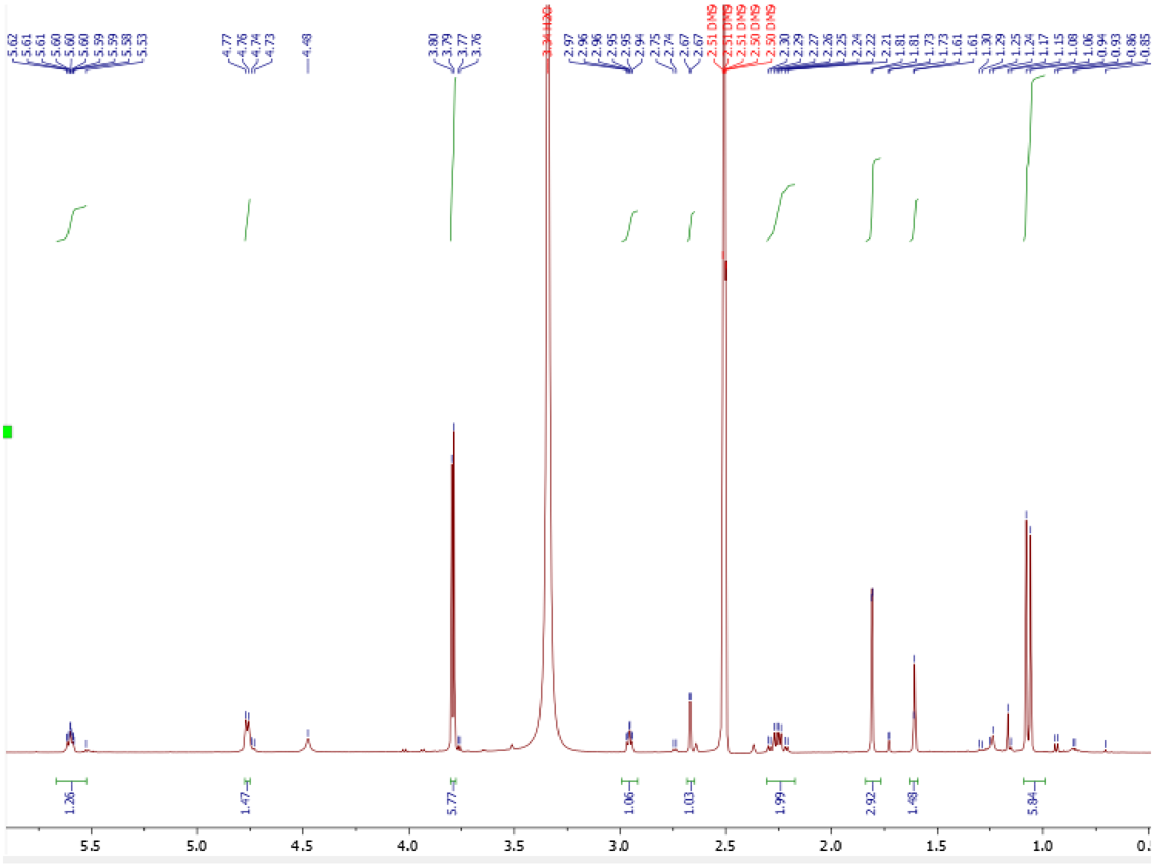

Figure S24. ^{1}H NMR spectrum (2) of **4** in DMSO

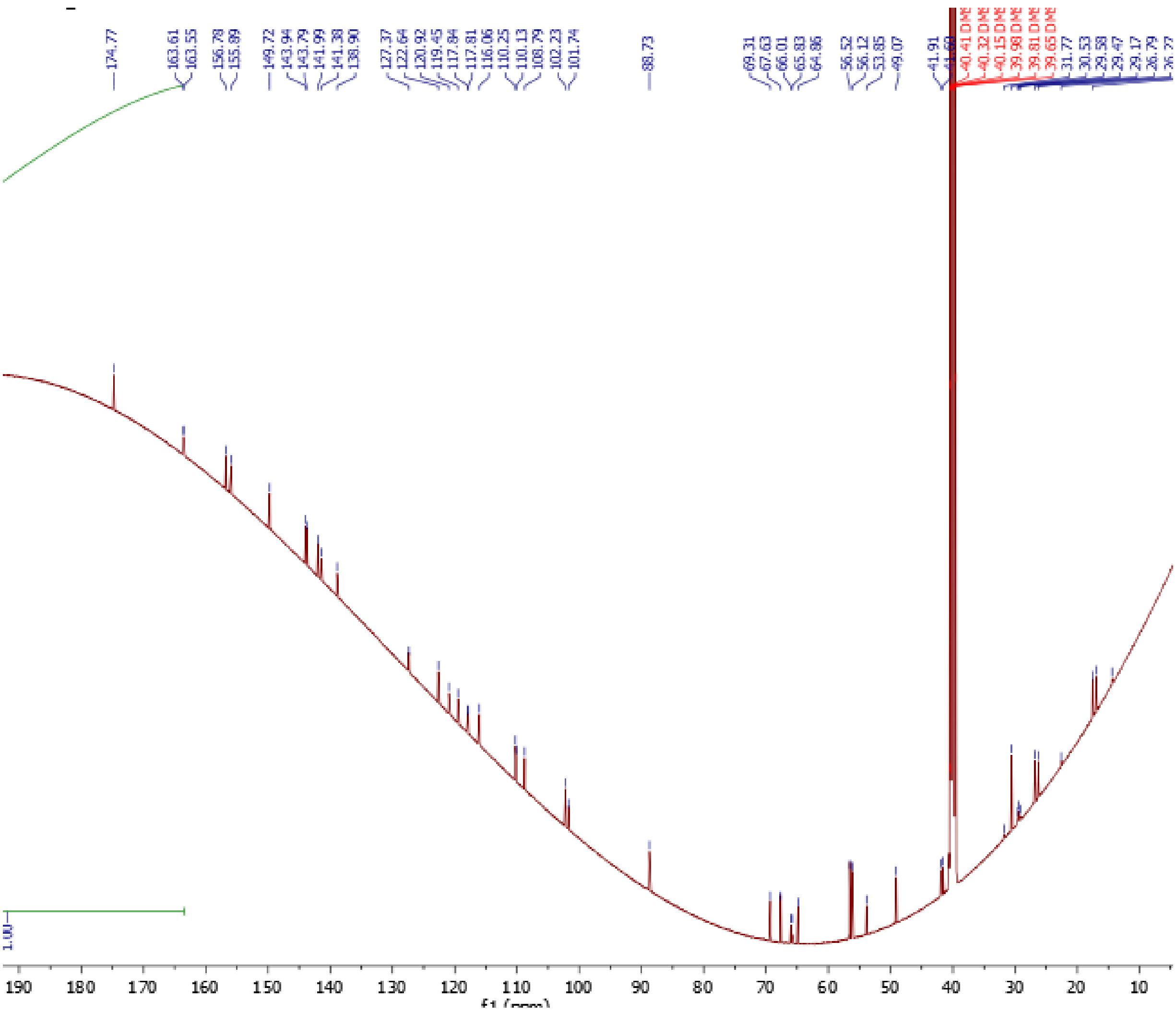

Figure S25. ^{13}C NMR spectrum of **4** in DMSO

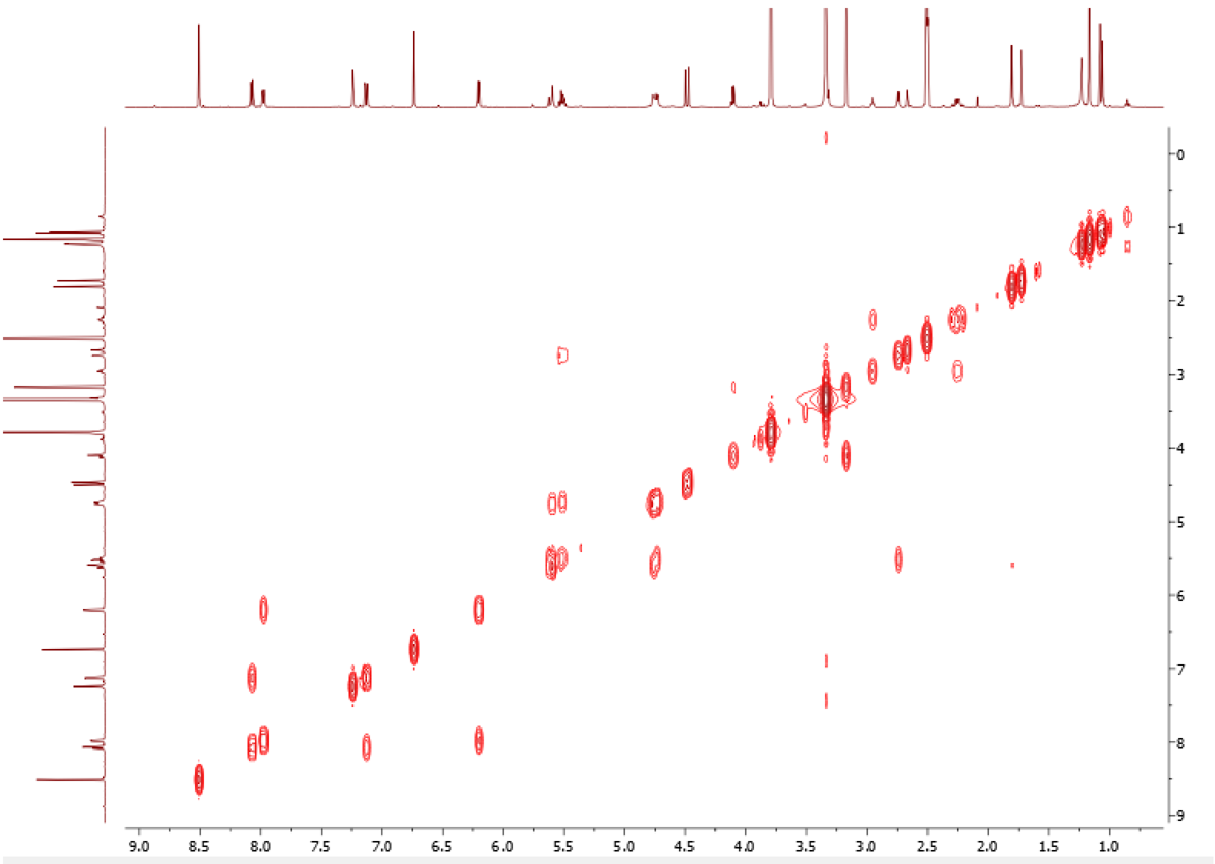

Figure S26. COSY spectrum of **4** in DMSO

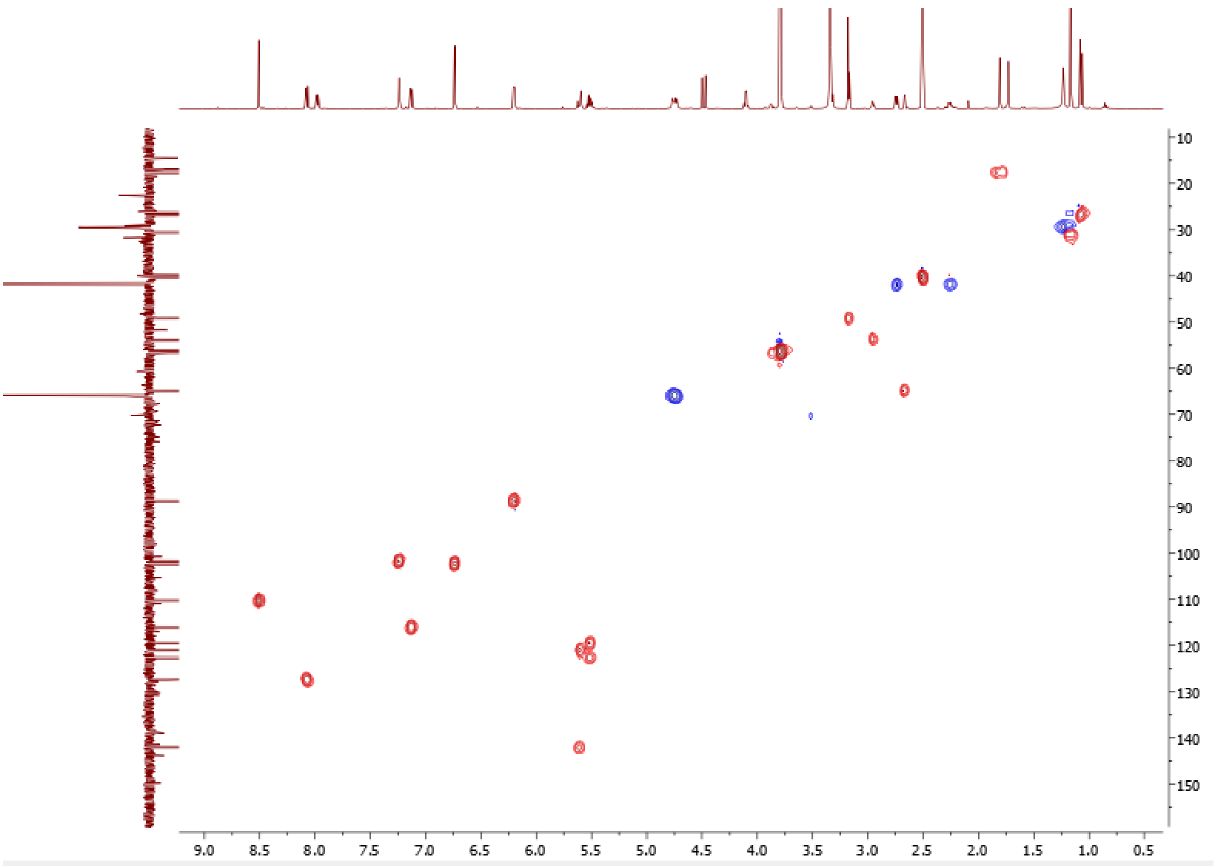

Figure S27. HSQC spectrum of **4** in DMSO

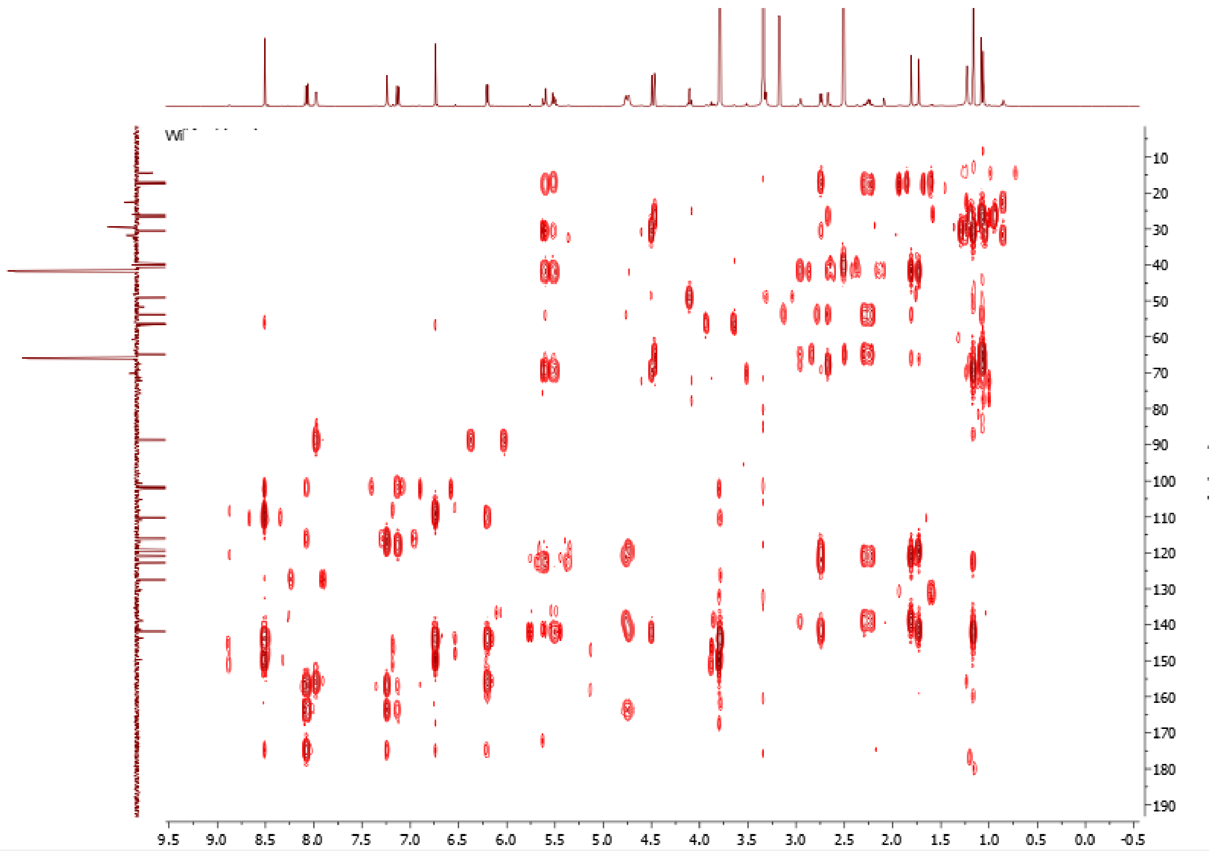

Figure S28. HMBC spectrum of **4** in DMSO

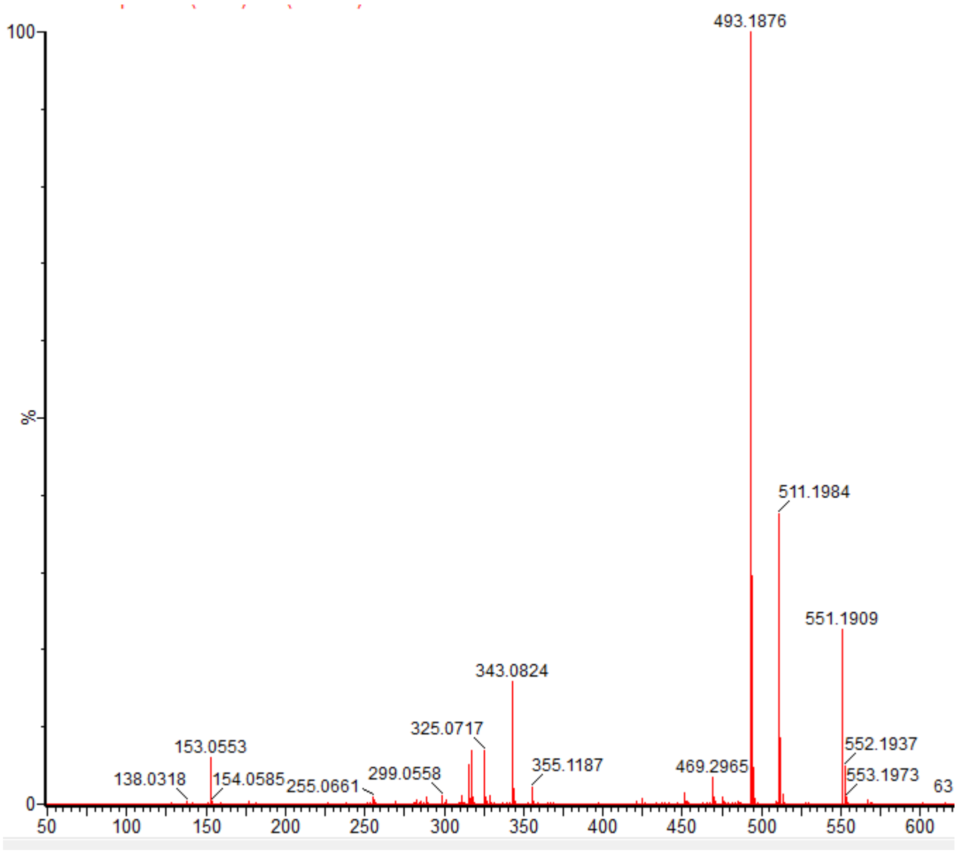

Figure S29. HRESIMS spectrum of **4**.

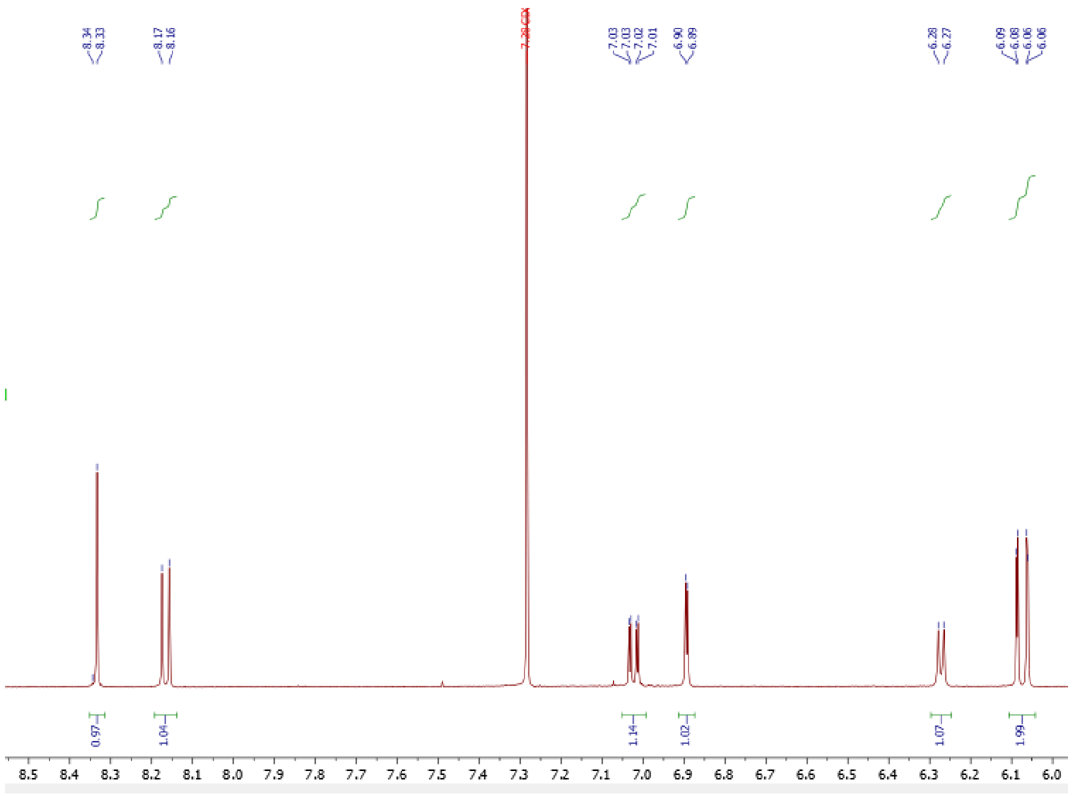

Figure S30. ^{1}H NMR spectrum (1) of **5** in $CDCl_3$.

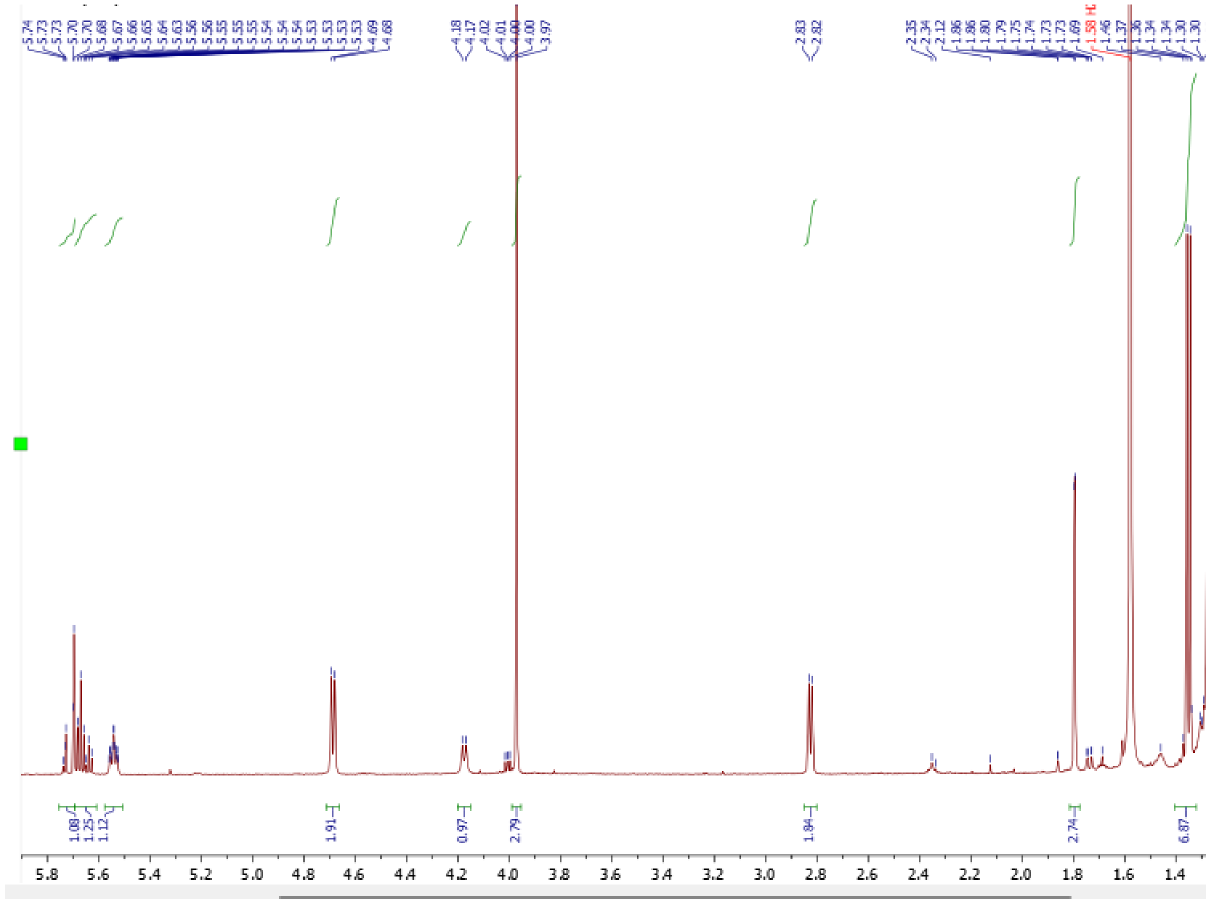

Figure S31. ^{1}H NMR spectrum (2) of **5** in $CDCl_3$.

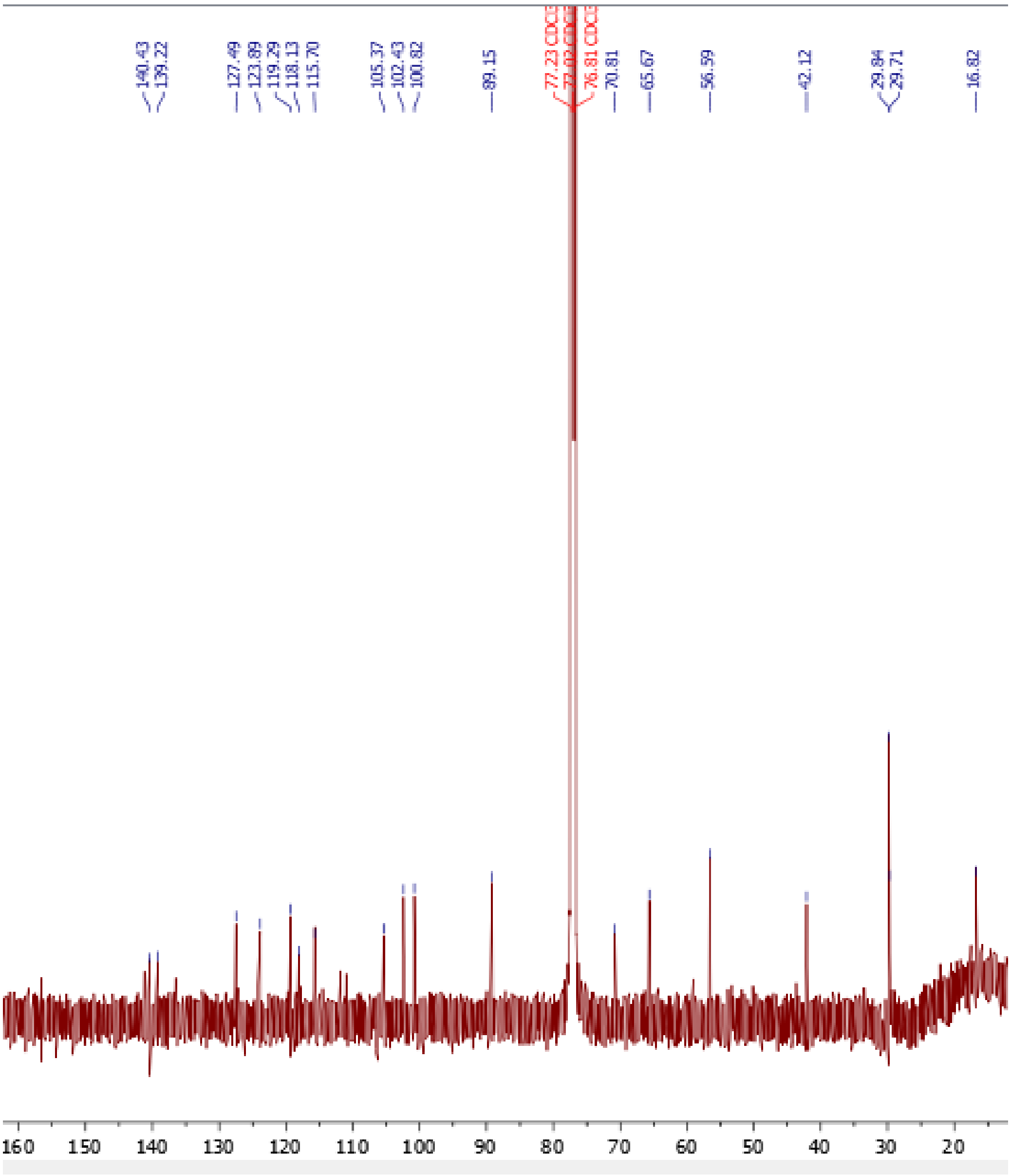

Figure S32. ^{13}C NMR spectrum of **5** in $CDCl_3$.

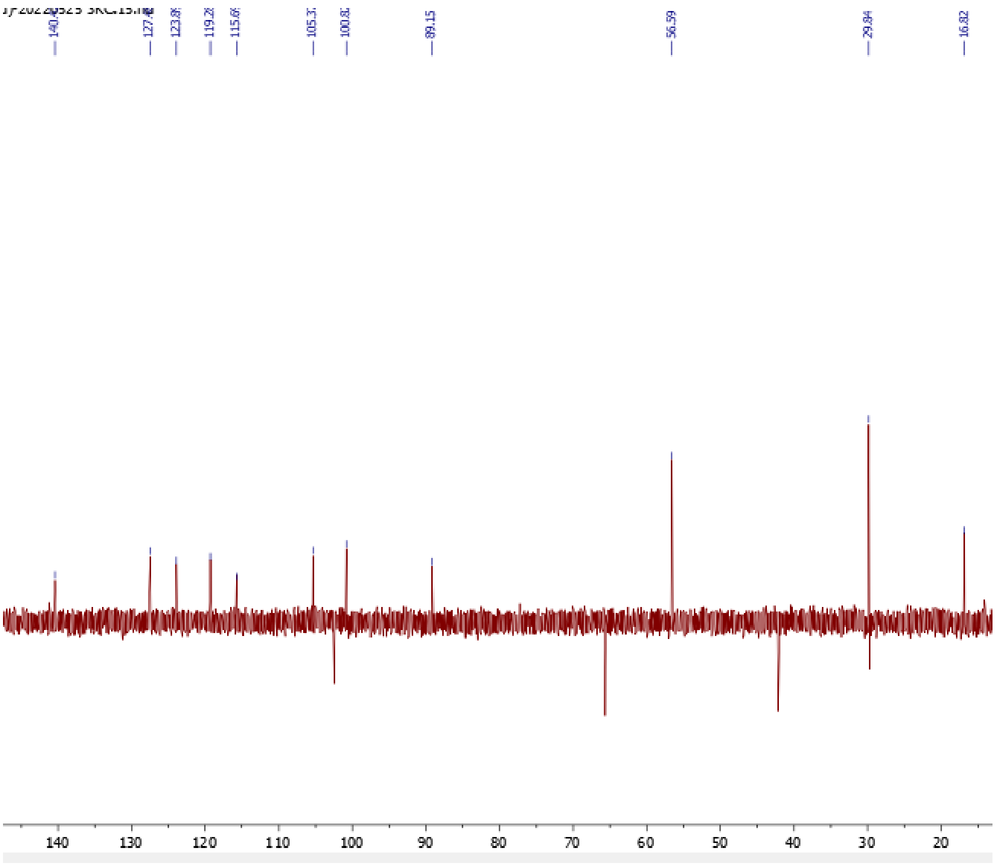

Figure S33. DEPT 135 spectrum of **5** in $CDCl_3$.

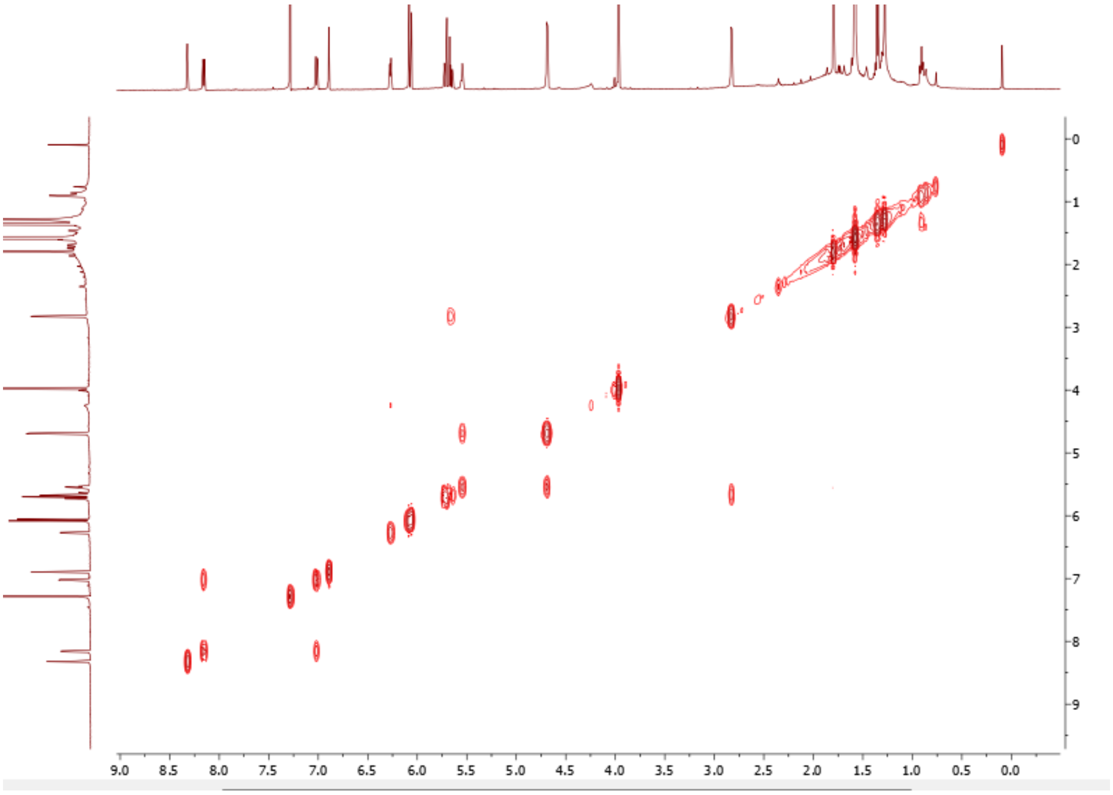

Figure S34. COSY spectrum of **5** in $CDCl_3$.

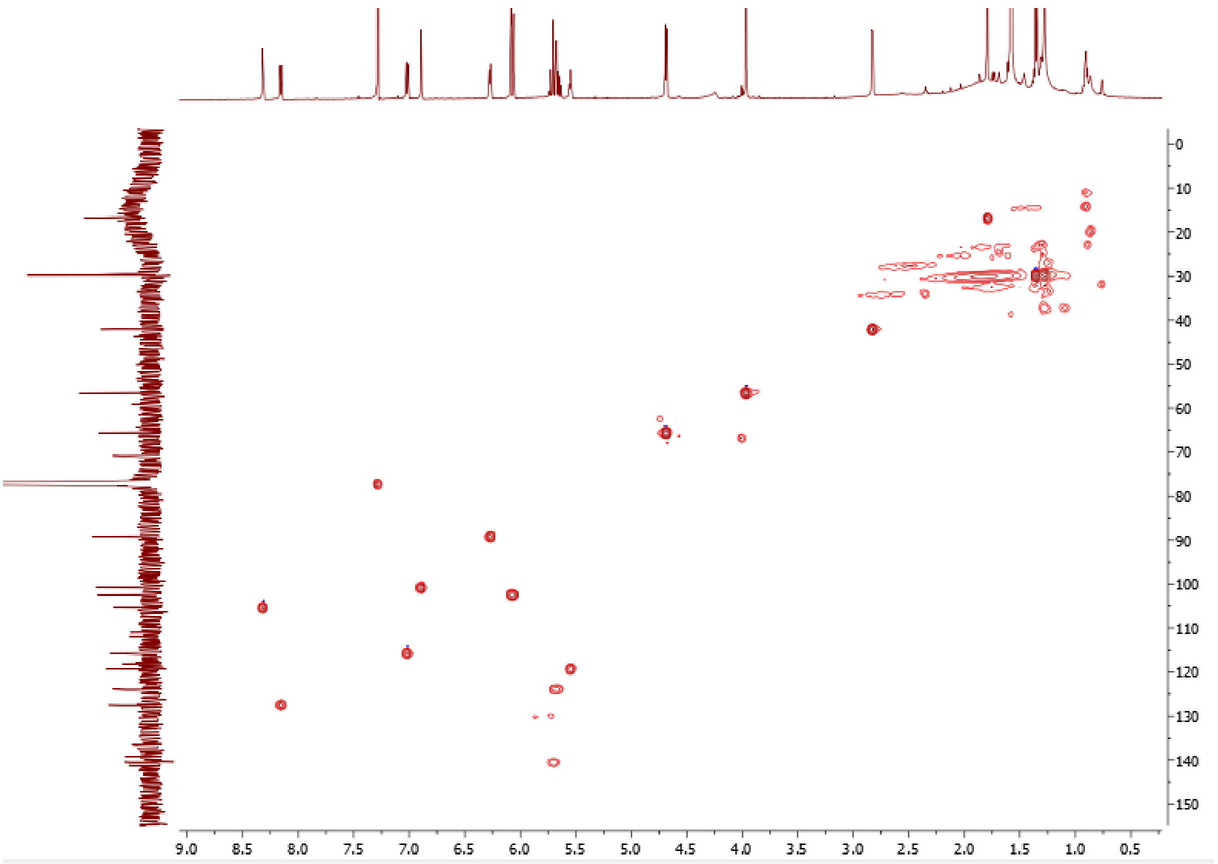

Figure S35. HSQC spectrum of **5** in $CDCl_3$.

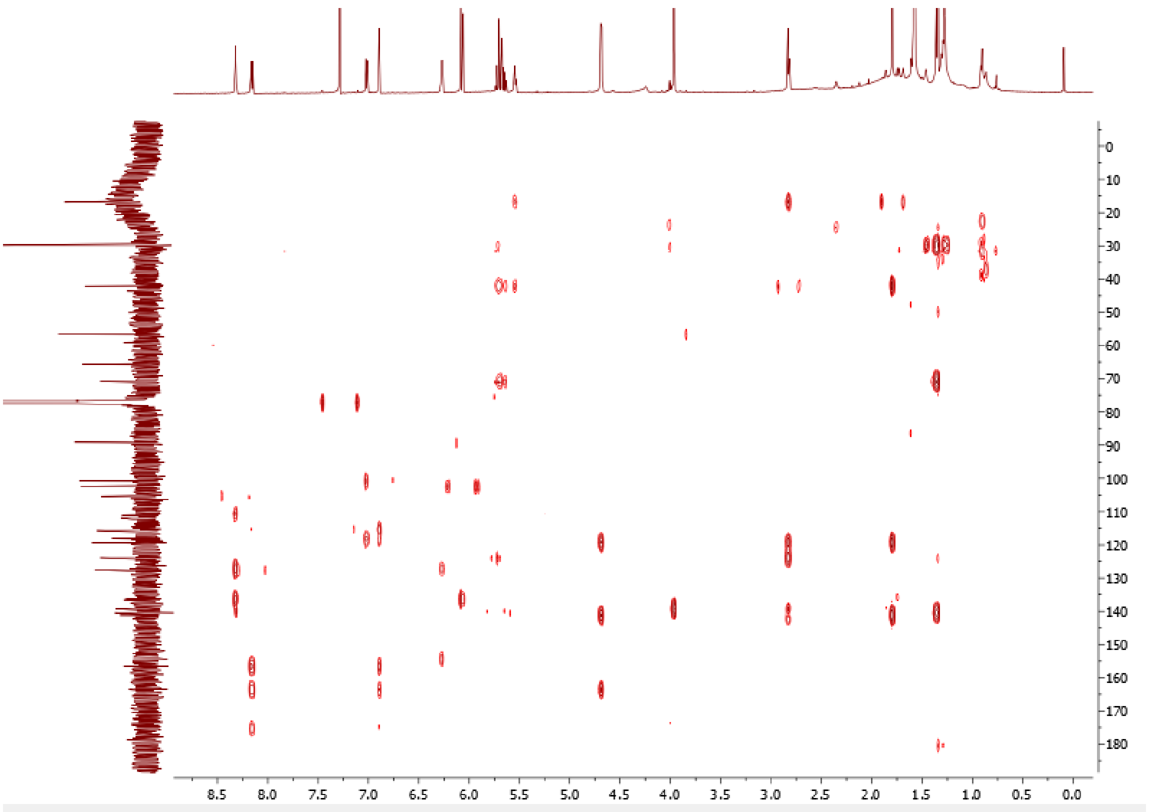

Figure S36. HMBC spectrum of **5** in $CDCl_3$.

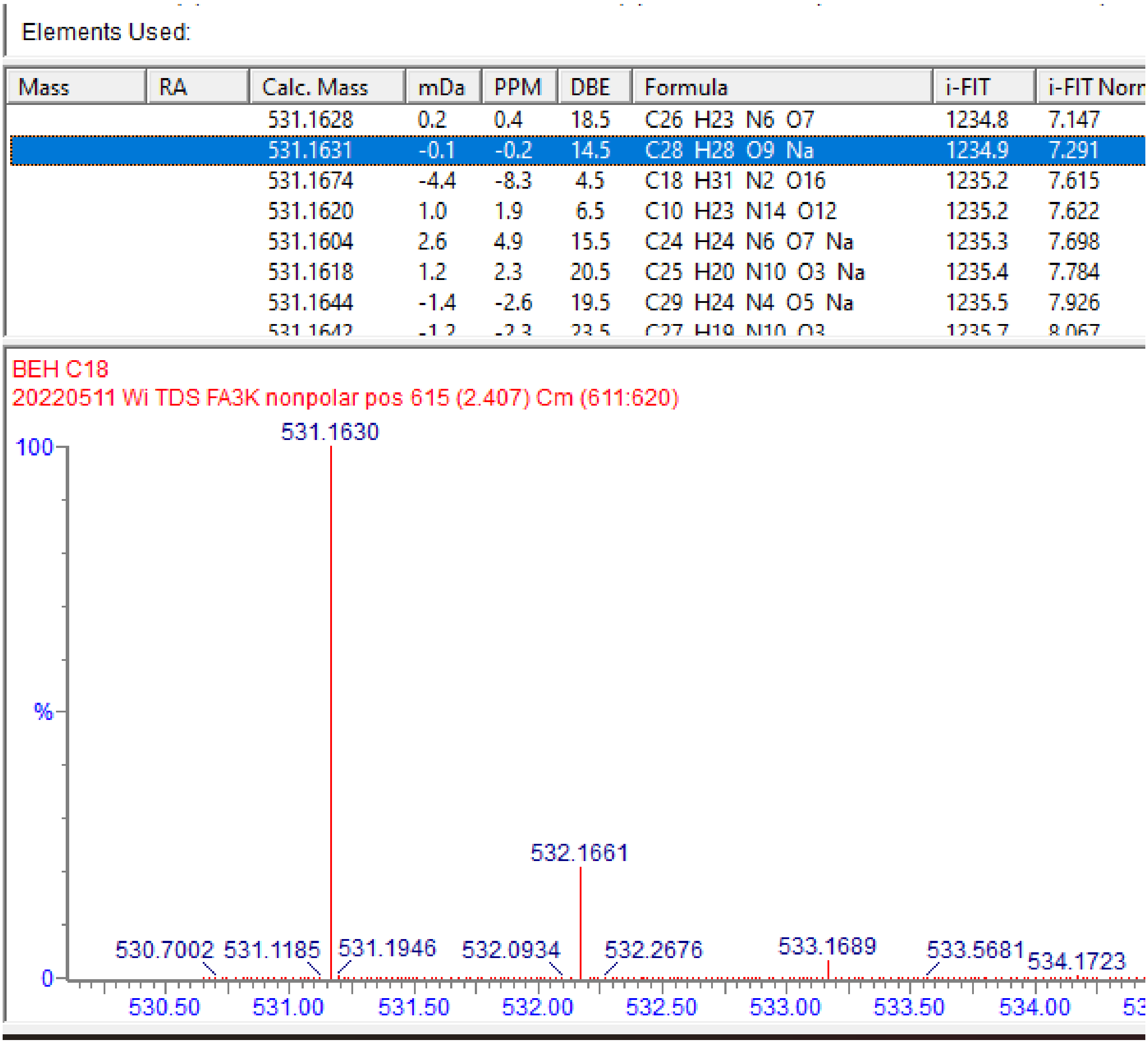

Elements Used:

Mass	RA	Calc. Mass	mDa	PPM	DBE	Formula	i-FIT	i-FIT Norr
		531.1628	0.2	0.4	18.5	C26 H23 N6 O7	1234.8	7.147
		531.1631	-0.1	-0.2	14.5	C28 H28 O9 Na	1234.9	7.291
		531.1674	-4.4	-8.3	4.5	C18 H31 N2 O16	1235.2	7.615
		531.1620	1.0	1.9	6.5	C10 H23 N14 O12	1235.2	7.622
		531.1604	2.6	4.9	15.5	C24 H24 N6 O7 Na	1235.3	7.698
		531.1618	1.2	2.3	20.5	C25 H20 N10 O3 Na	1235.4	7.784
		531.1644	-1.4	-2.6	19.5	C29 H24 N4 O5 Na	1235.5	7.926
		531.1642	-1.2	-2.3	23.5	C27 H19 N10 O3	1235.7	8.067

Figure S37. HRESIMS spectrum of **5** in CDCL3.

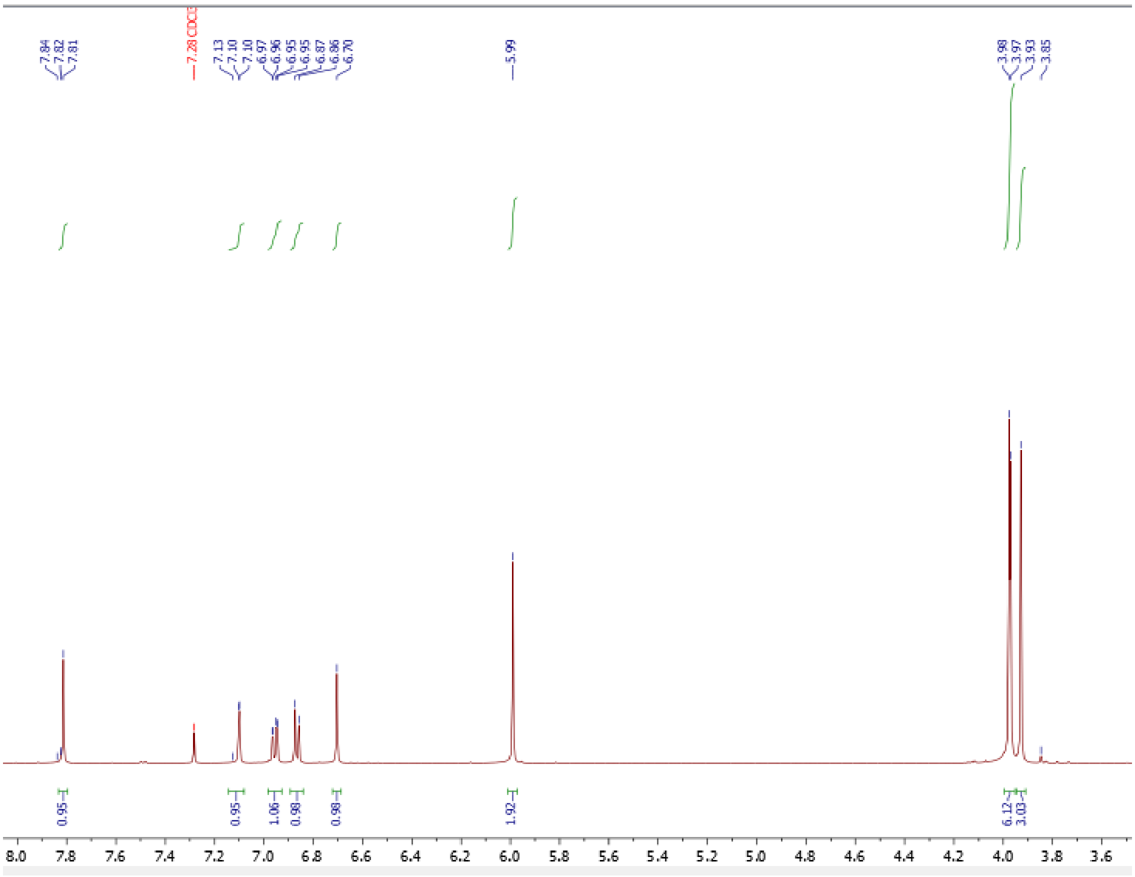

Figure S38. ^{1}H NMR spectrum (500MHz, $CDCl_3$) of compound **6.**

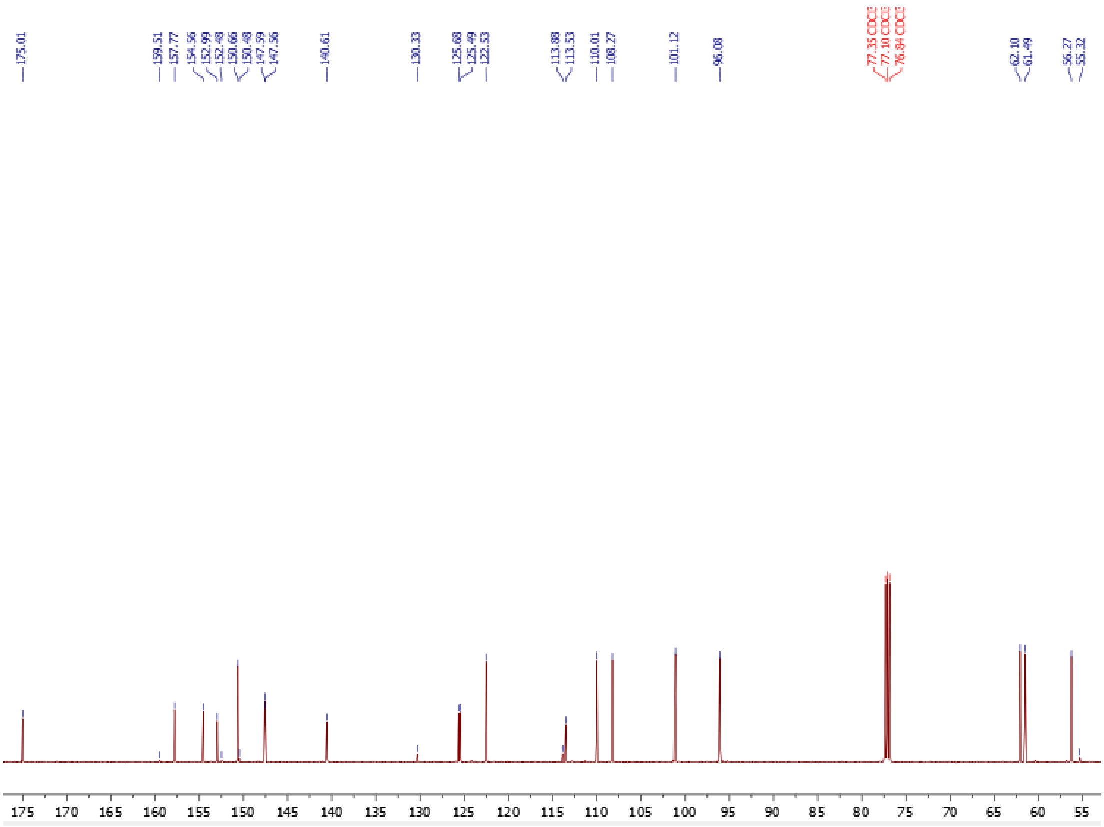

Figure S39. ^{13}C NMR spectrum (125 MHz, $CDCL_3$) of the compound **6.**

Elements Used:

Mass	RA	Calc. Mass	mDa	PPM	DBE	Formula	i-FIT	i-FIT Norm	Fit Con
357.0976	100.00	357.1020	-4.4	-12.3	8.5	C9 H13 N10 O6	748.8	0.354	70.20
		357.0974	0.2	0.6	11.5	C19 H17 O7	751.0	2.572	7.64
		357.0988	-1.2	-3.4	16.5	C20 H13 N4 O3	751.4	2.965	5.15
		357.1006	-3.0	-8.4	3.5	C8 H17 N6 O10	751.7	3.267	3.81
		357.0934	4.2	11.8	7.5	C14 H17 N2 O9	751.8	3.353	3.50
		357.0947	2.9	8.1	12.5	C15 H13 N6 O5	751.8	3.411	3.30
		357.0993	-1.7	-4.8	9.5	C5 H9 N16 O4	751.8	3.425	3.26
		357.1006	-3.0	-8.4	14.5	C6 H5 N20	752.8	4.419	1.20

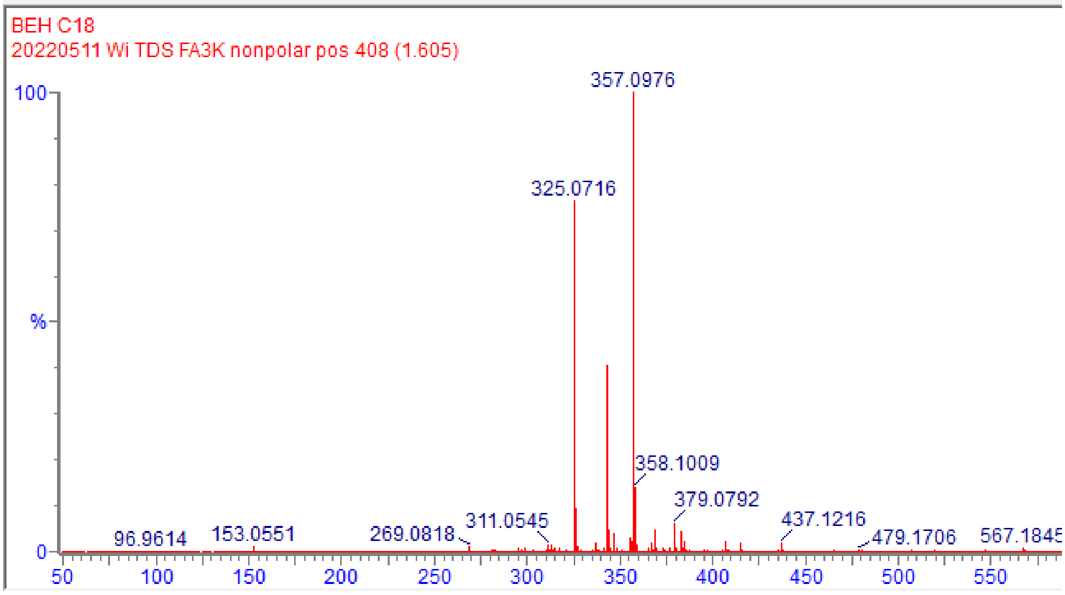

Figure S40. HRESIMS spectrum of **6.**

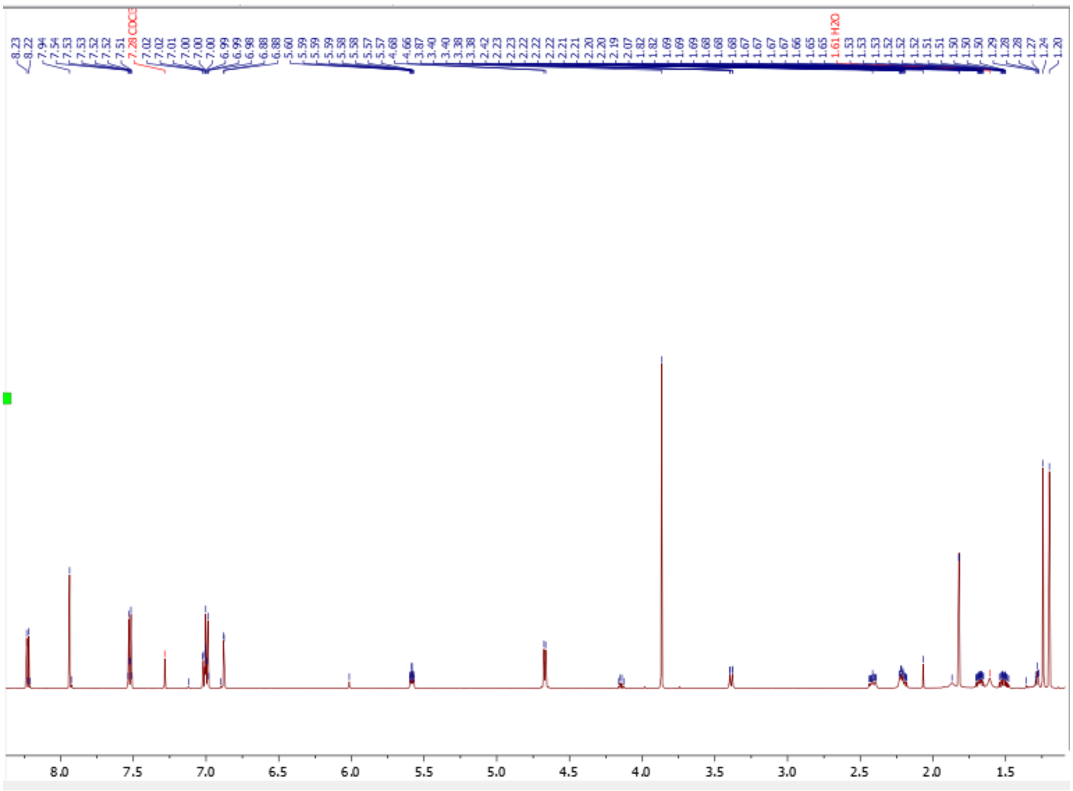

Figure S41. ^{1}H NMR spectrum (500MHz, CDCl3) of compound **7.**

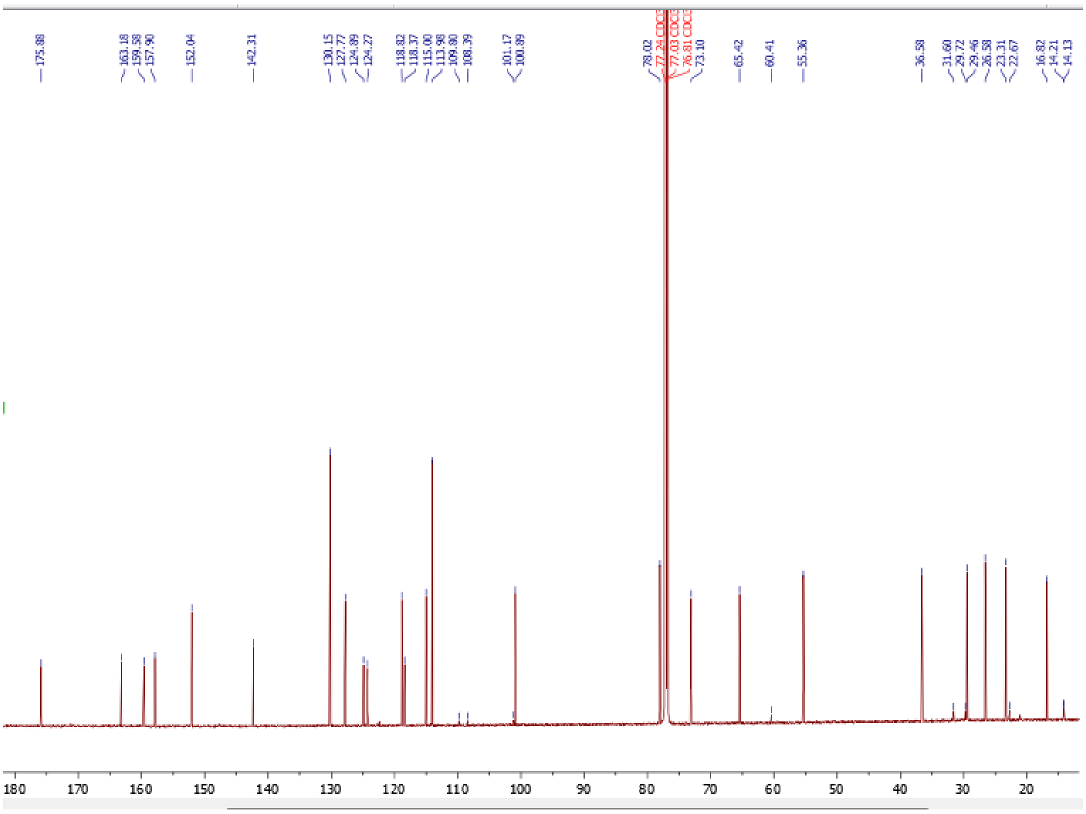

Figure S42. ^{13}C NMR spectrum (125MHz, $CDCl_3$) of compound **7.**

[illegible]

Elements Used:

Mass	RA	Calc. Mass	mDa	PPM	DBE	Formula	i-FIT	i-FIT Norm	Fit Conf %
461.1987	100.00	461.1999	-1.2	-2.6	2.5	C19 H34 O11 Na	865.2	0.068	93.43
		461.1964	2.3	5.0	14.5	C28 H29 O6	870.0	4.936	0.72
		461.2023	-3.6	-7.8	5.5	C21 H33 O11	868.0	2.914	5.42
		461.1940	4.7	10.2	11.5	C26 H30 O6 Na	870.6	5.456	0.43

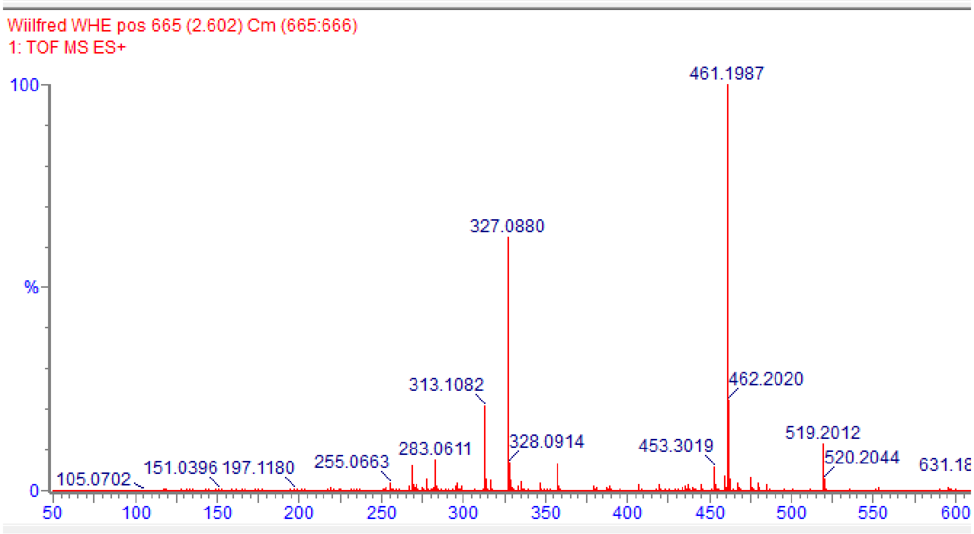

Figure S43. HRESIMS spectrum of **7.**

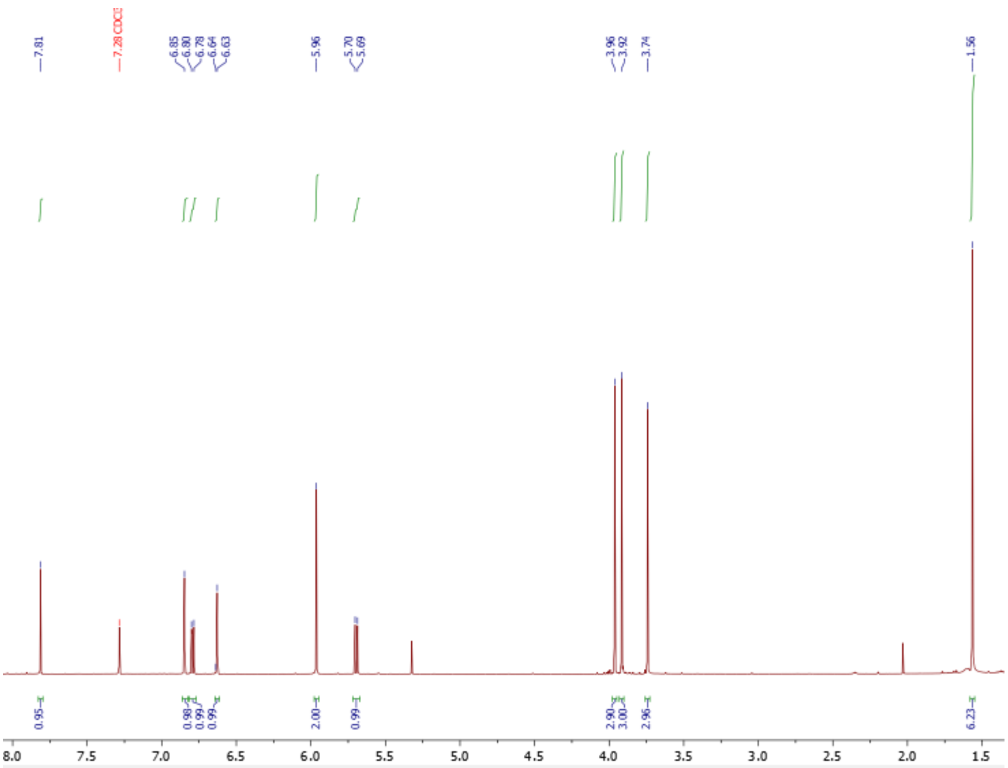

Figure S44. ^{1}H NMR (500 MHz, $CDCl_3$) spectrum of **8.**

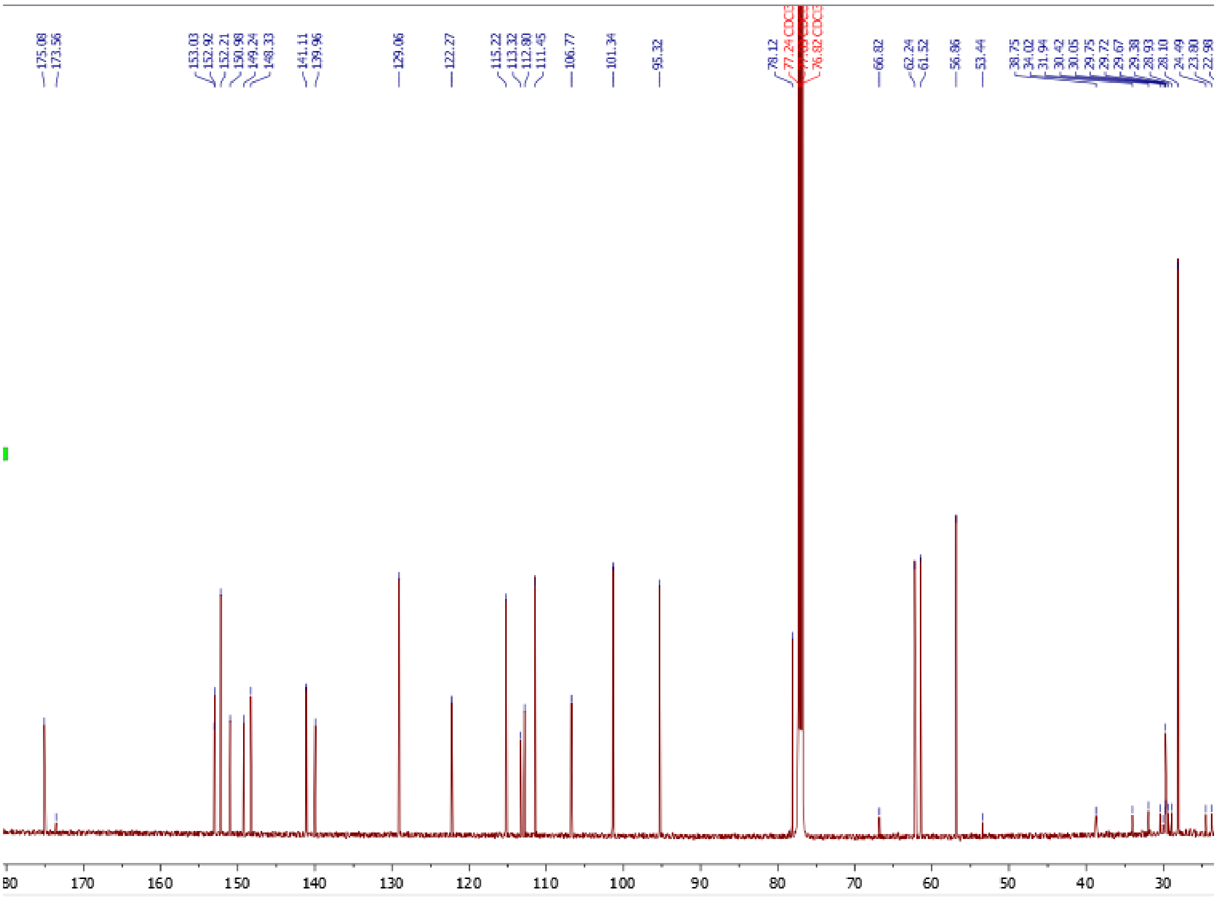

Figure S45. ^{13}C NMR (125MHz, $CDCl_3$) spectrum of **8.**

Elements Used:

Mass	RA	Calc. Mass	mDa	PPM	DBE	Formula	i-FIT	i-FIT Norm	Fit Conf %	C
439.1395	100.00	439.1393	0.2	0.5	13.5	C24 H23 O8	963.2	2.532	7.95	24
		439.1369	2.6	5.9	10.5	C22 H24 O8 Na	962.8	2.131	11.87	22
		439.1428	-3.3	-7.5	1.5	C15 H28 O13 Na	960.9	0.221	80.18	15

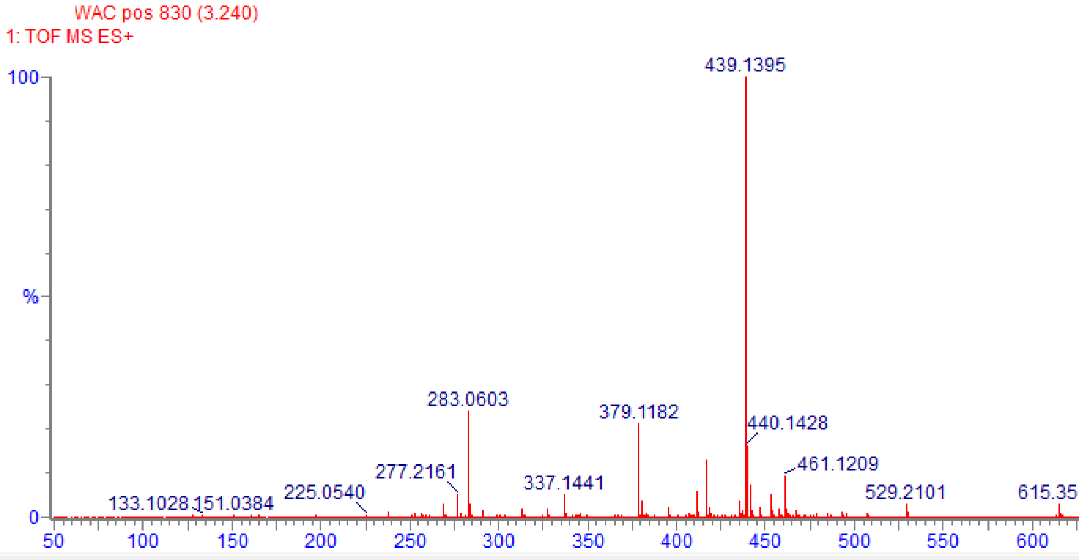

Figure S46. HRESIMS spectrum of **8.**

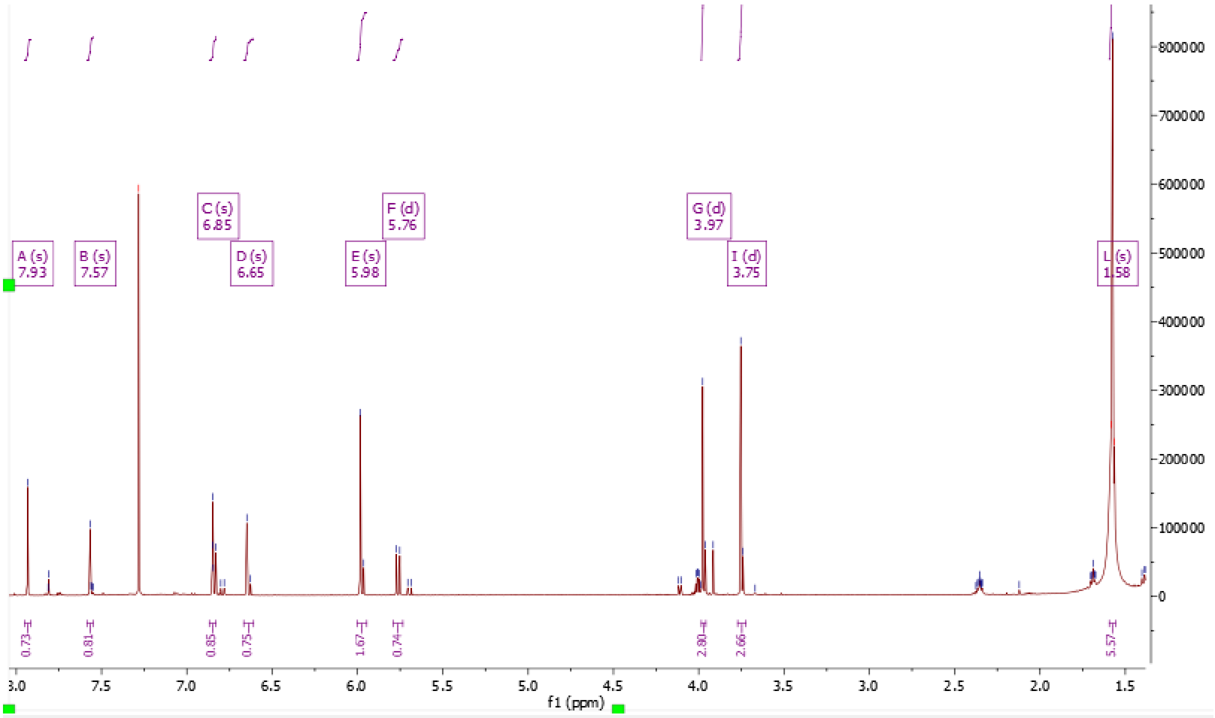

Figure S47. ^{1}H NMR (500MHz, $CDCl_3$) spectrum of **9**.

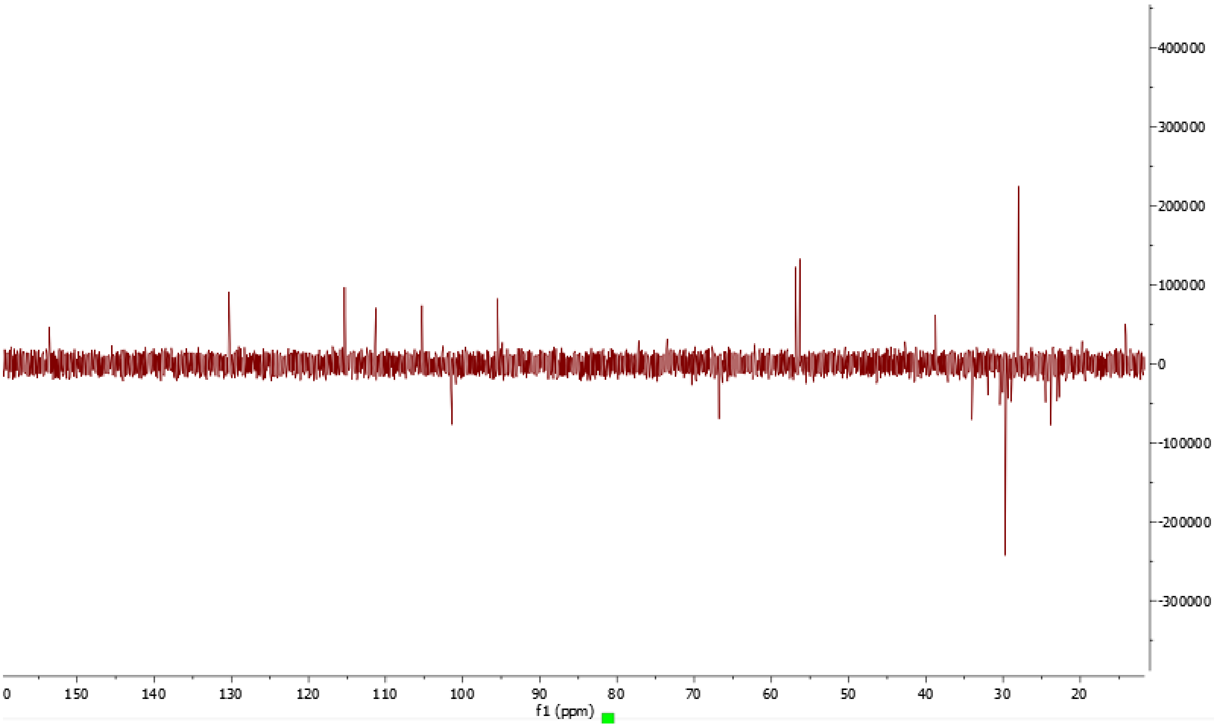

Figure S48. ^{13}C NMR (125MHz, $CDCl_3$) spectrum of **9**.

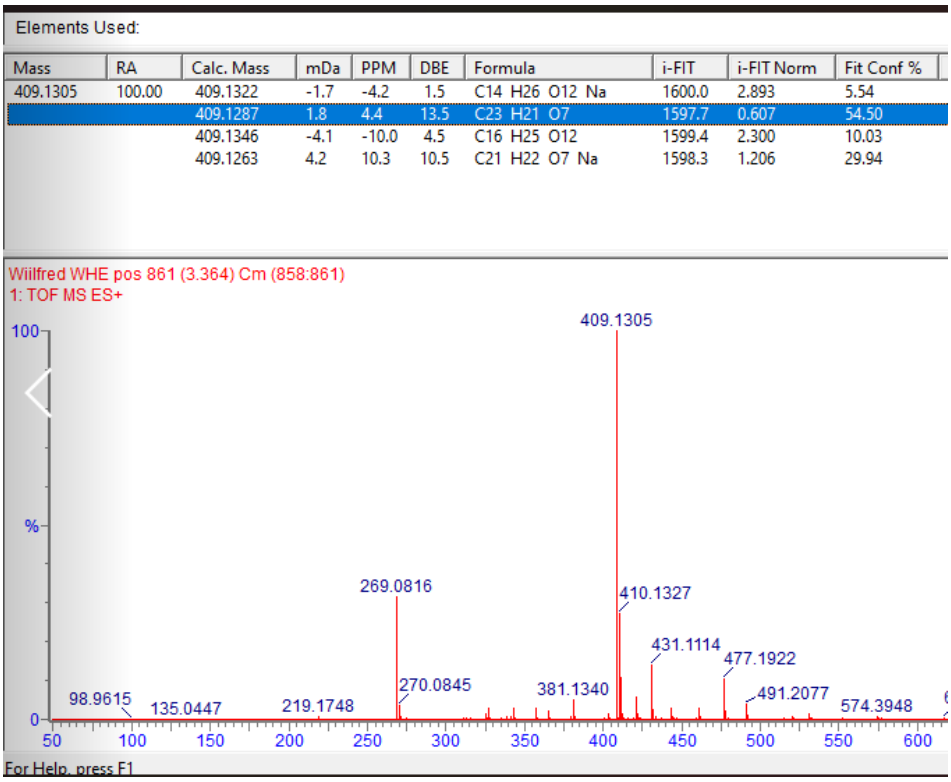

Elements Used:

Mass	RA	Calc. Mass	mDa	PPM	DBE	Formula	i-FIT	i-FIT Norm	Fit Conf %
409.1305	100.00	409.1322	-1.7	-4.2	1.5	C14 H26 O12 Na	1600.0	2.893	5.54
		409.1287	1.8	4.4	13.5	C23 H21 O7	1597.7	0.607	54.50
		409.1346	-4.1	-10.0	4.5	C16 H25 O12	1599.4	2.300	10.03
		409.1263	4.2	10.3	10.5	C21 H22 O7 Na	1598.3	1.206	29.94

Figure S49. HRESIMS spectrum of **9**.

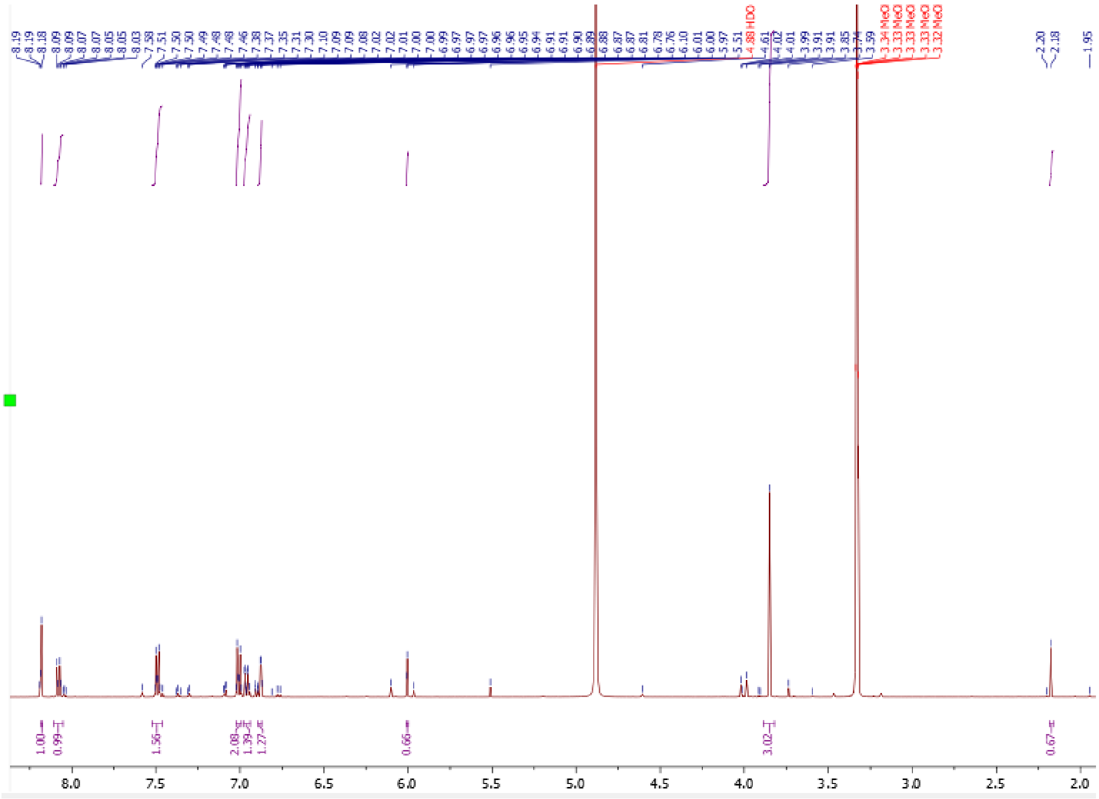

Figure S50. ^{1}H NMR (500MHz, MeOD) spectrum of **10.**

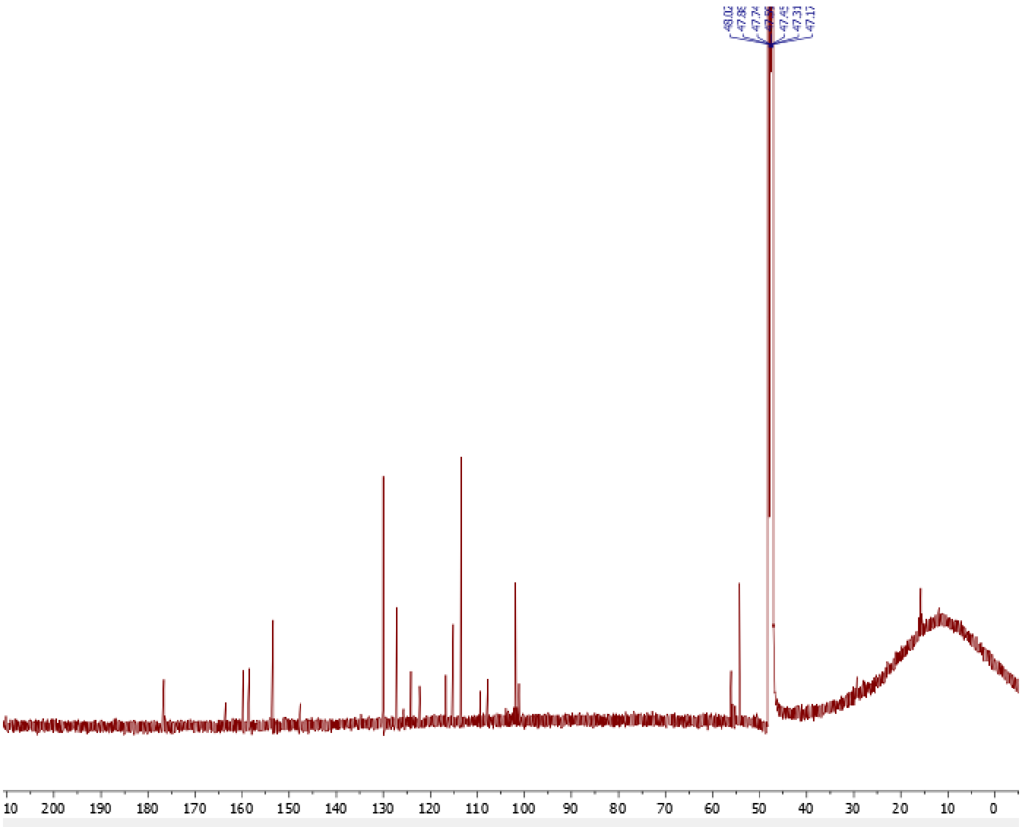

Figure S51. ^{13}C NMR (125MHz, MeOD) spectrum of **10.**

Elements Used:

Mass	RA	Calc. Mass	mDa	PPM	DBE	Formula	i-FIT	i-FIT Norm	Fit Conf %
269.0838	100.00	269.0814	2.4	8.9	10.5	C16 H13 O4	1341.4	0.389	67.76
		269.0790	4.8	17.8	7.5	C14 H14 O4 Na	1342.3	1.241	28.91
		269.0873	-3.5	-13.0	1.5	C9 H17 O9	1344.9	3.894	2.04
		269.0849	-1.1	-4.1	-1.5	C7 H18 O9 Na	1345.4	4.349	1.29

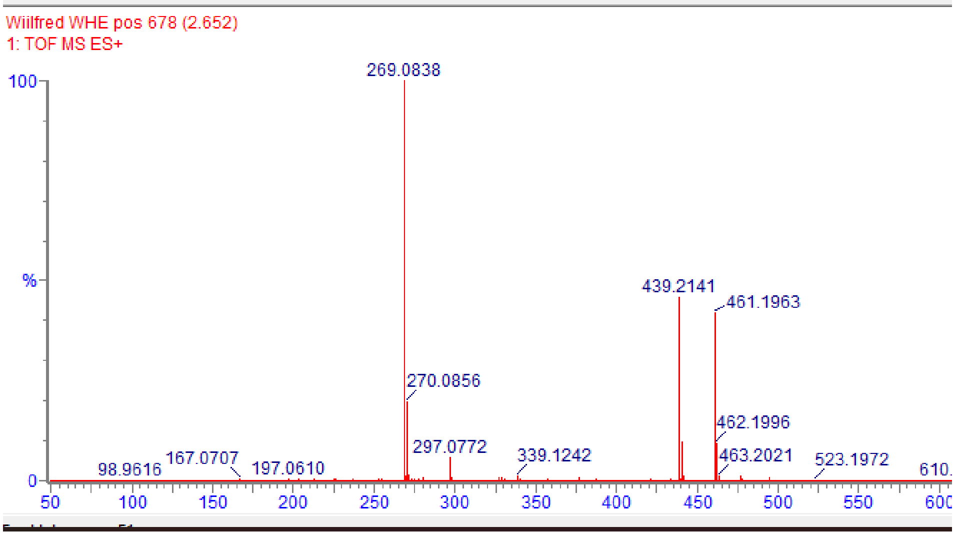

Figure S52. HRESIMS spectrum of 10.

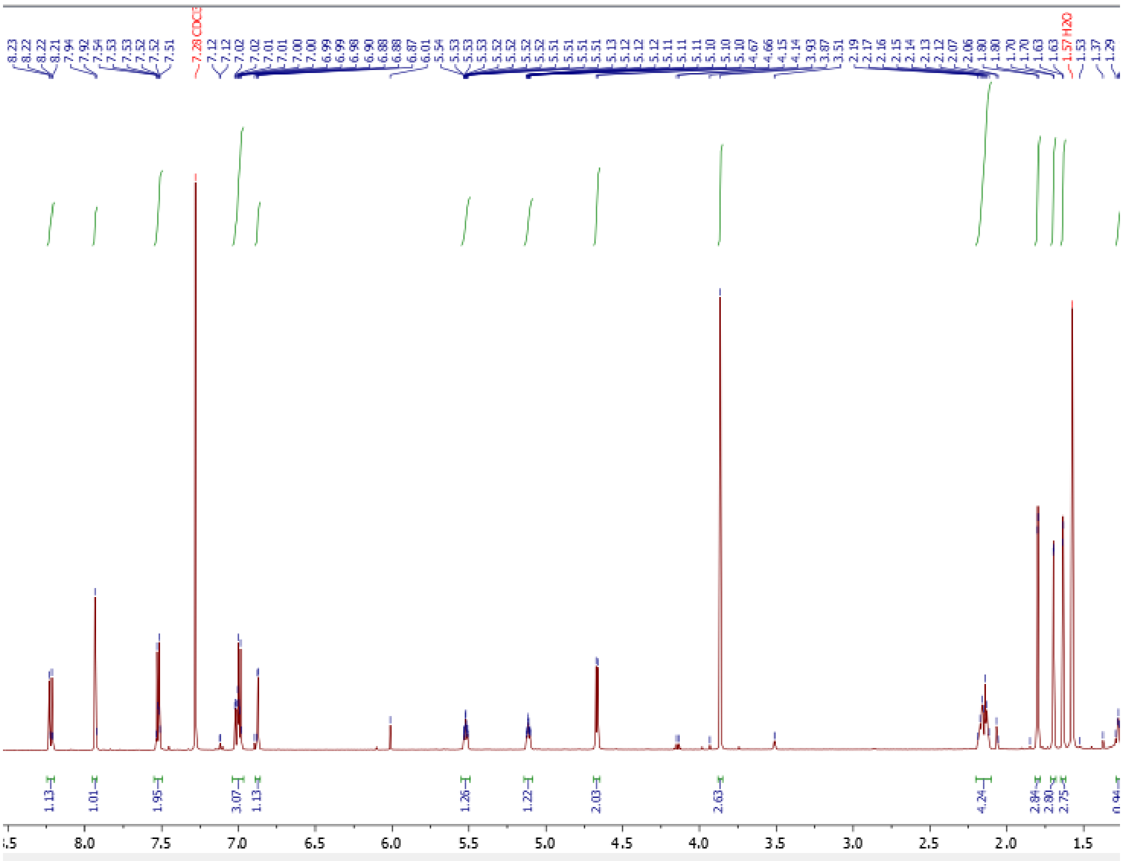

Figure S53. ^{1}H NMR (500 MHz, $CDCl_3$) spectrum of **11.**

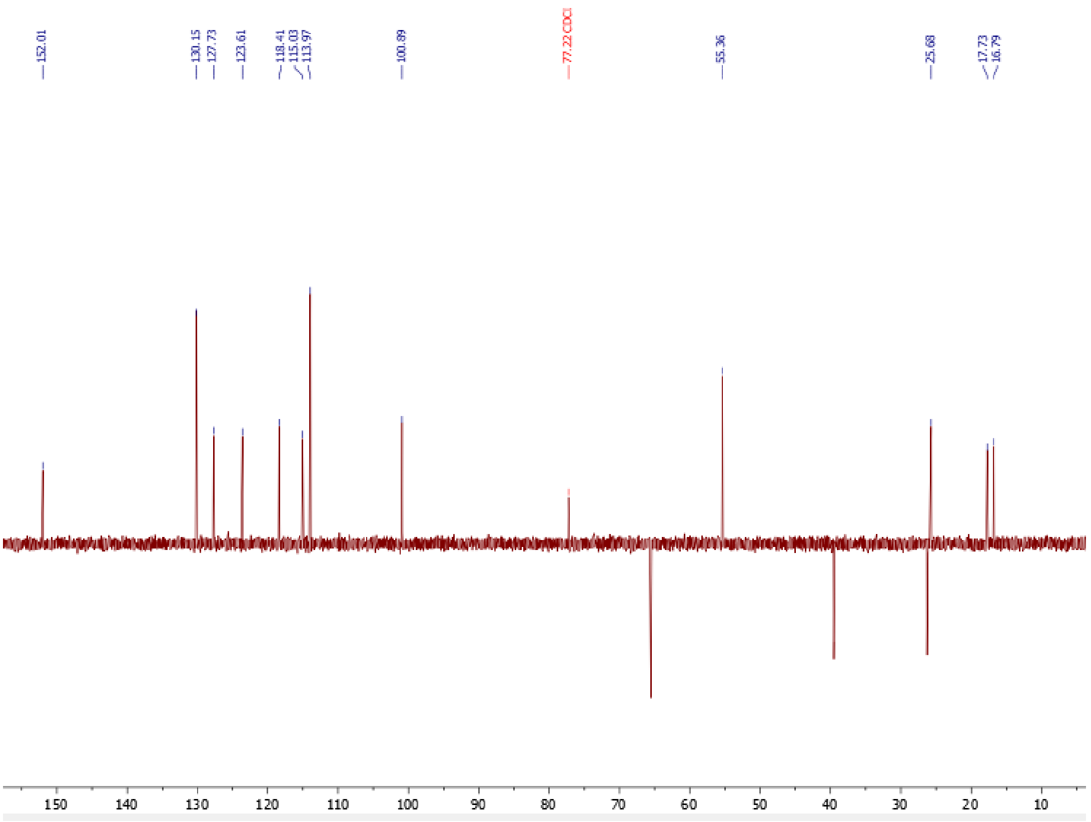

Figure S54. 135 DEPT (125MHz, $CDCl_3$) spectrum of **11.**

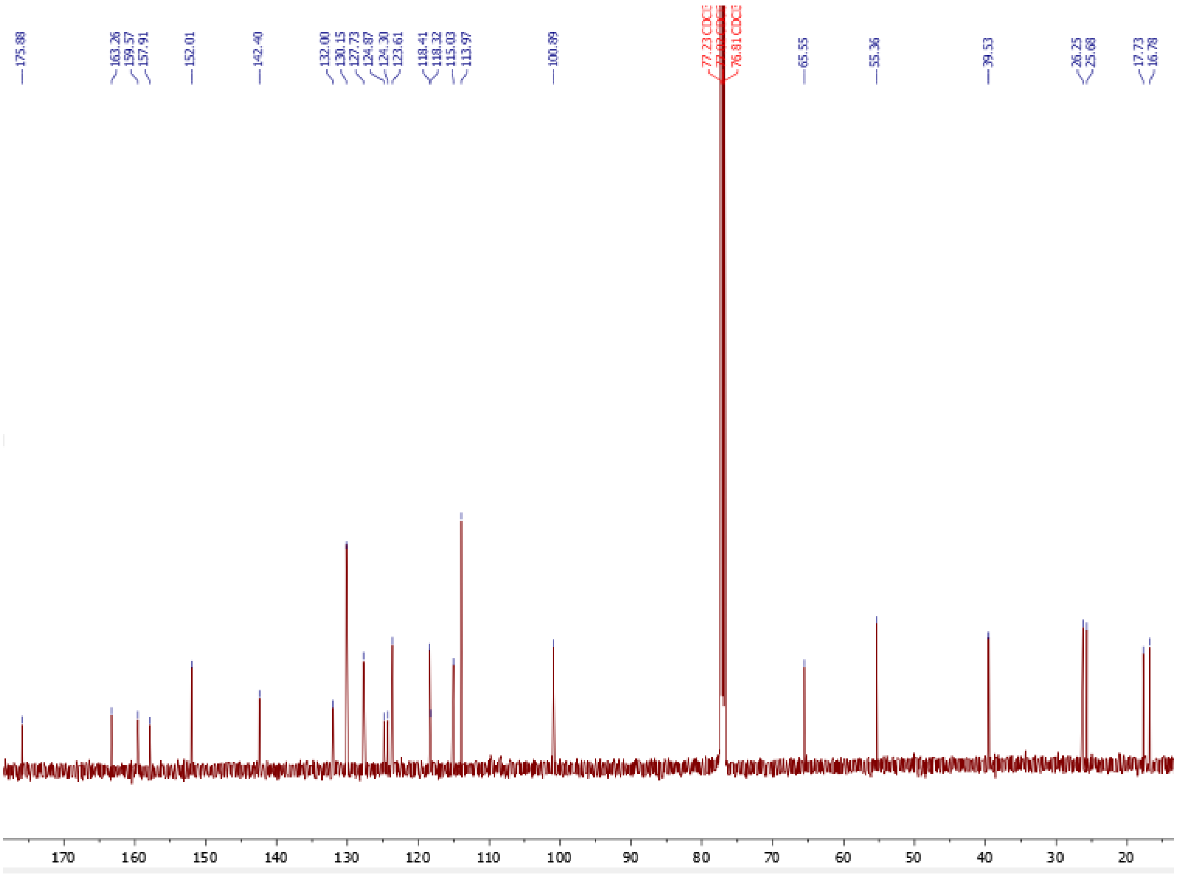

Figure S55. ^{13}C NMR (125MHz, $CDCl_3$) spectrum of **11**.

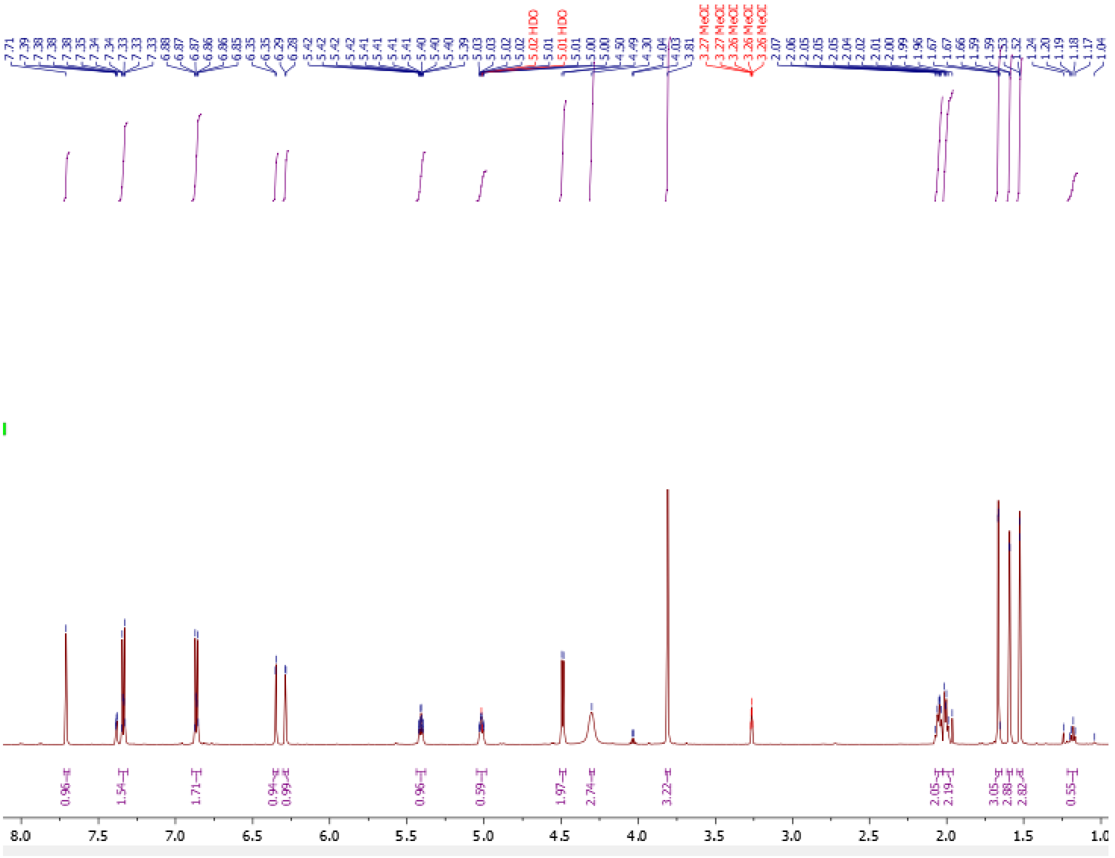

Figure S56. ^{1}H NMR (500 MHz, MeOD) spectrum of **12.**

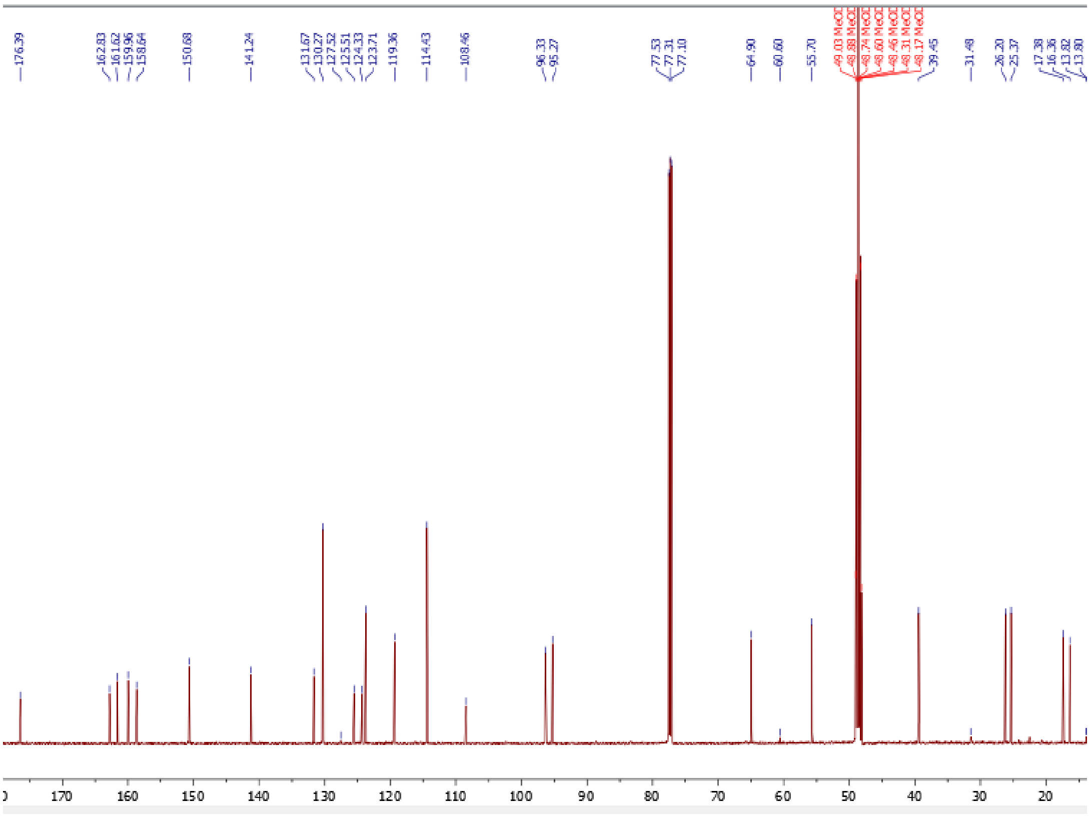

Figure S57. ^{13}C NMR (125MHz, MeOD) spectrum of **12.**

Elements Used:

Mass	RA	Calc. Mass	mDa	PPM	DBE	Formula	i-FIT	i-FIT Norm	Fit Conf %
421.2014	100.00	421.2015	-0.1	-0.2	12.5	C26 H29 O5	867.5	1.271	28.06
		421.1991	2.3	5.5	9.5	C24 H30 O5 Na	867.6	1.438	23.74
		421.2050	-3.6	-8.5	0.5	C17 H34 O10 Na	866.9	0.730	48.21

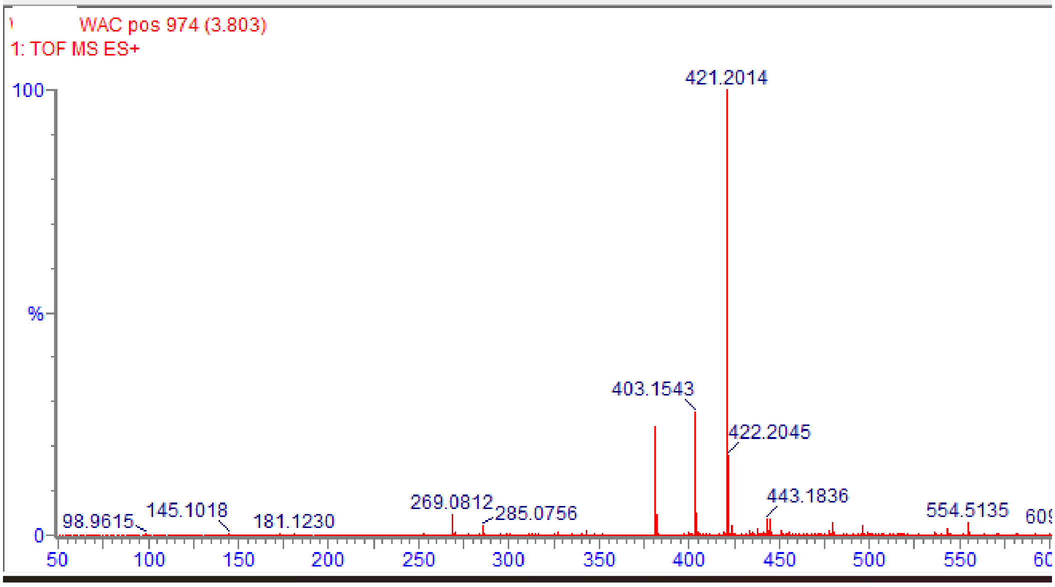

Figure S58. HRESIMS spectrum of **12**.

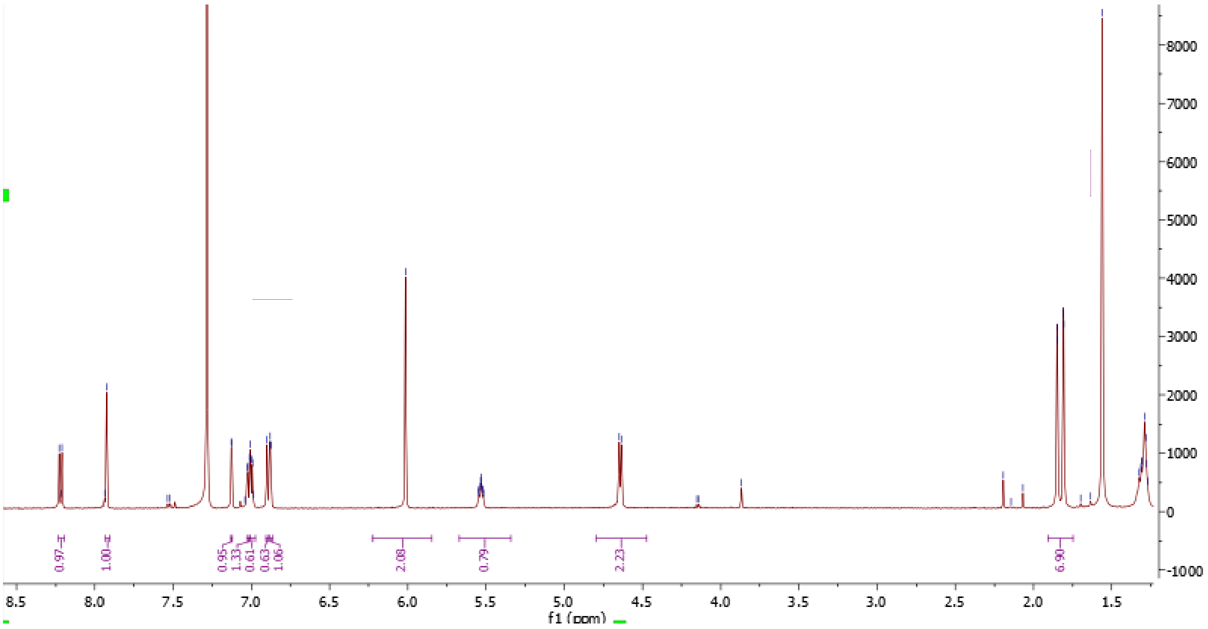

Figure S59. ^{1}H NMR (500 MHz, $CDCl_3$) spectrum of **13.**

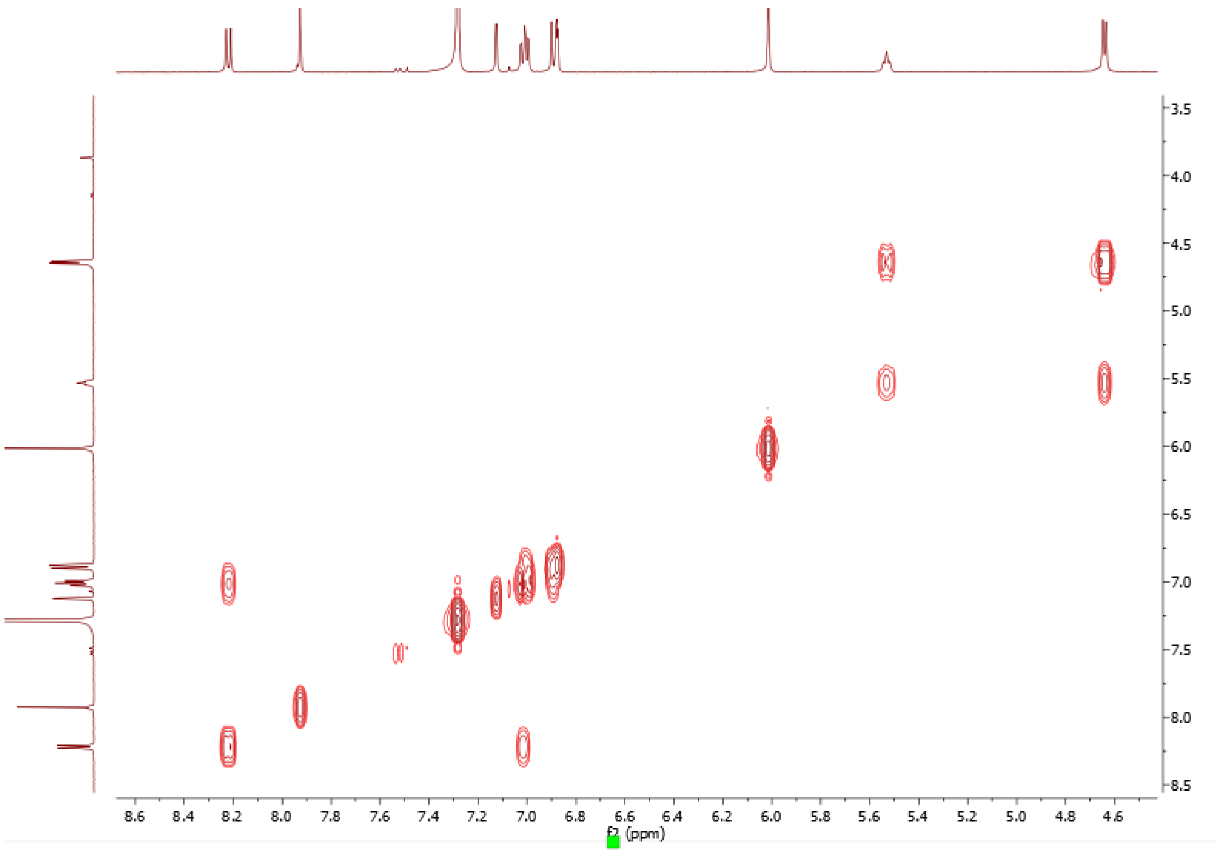

Figure S60. ^{1}H-^{1}H COSY (125MHz, $CDCl_3$) spectrum of **13.**

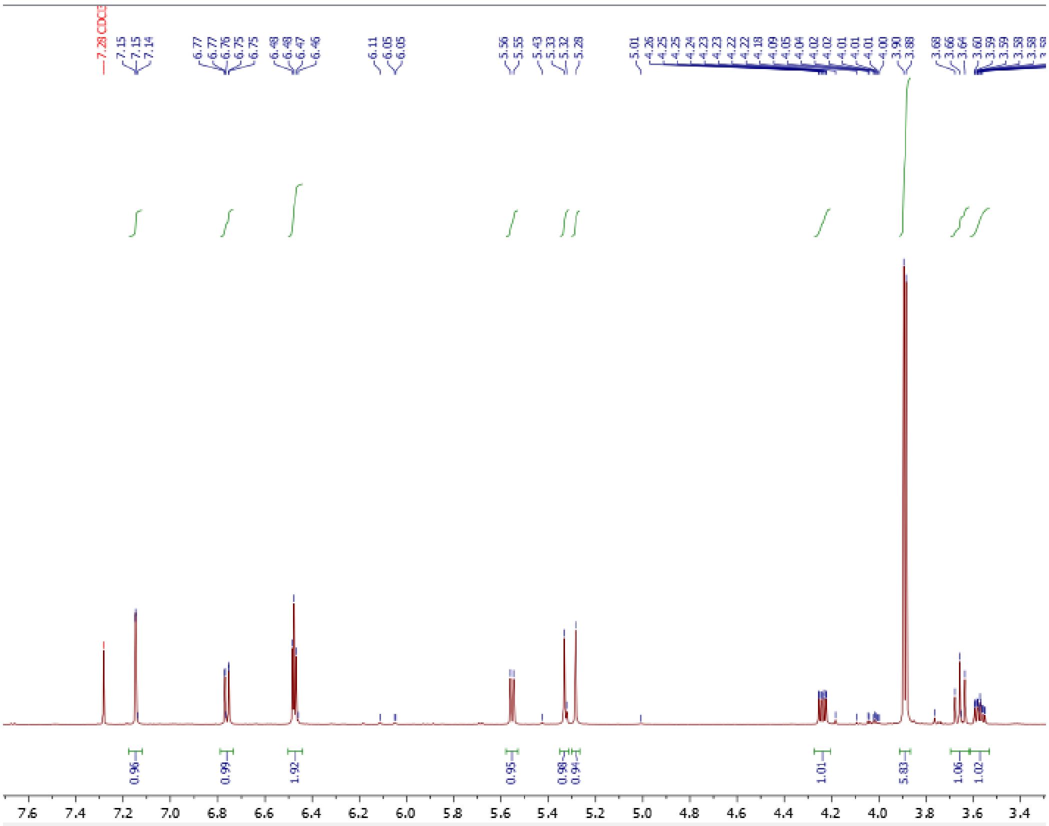

Figure S61. ^{1}H NMR (500 MHz, $CDCl_3$) spectrum of **14.**

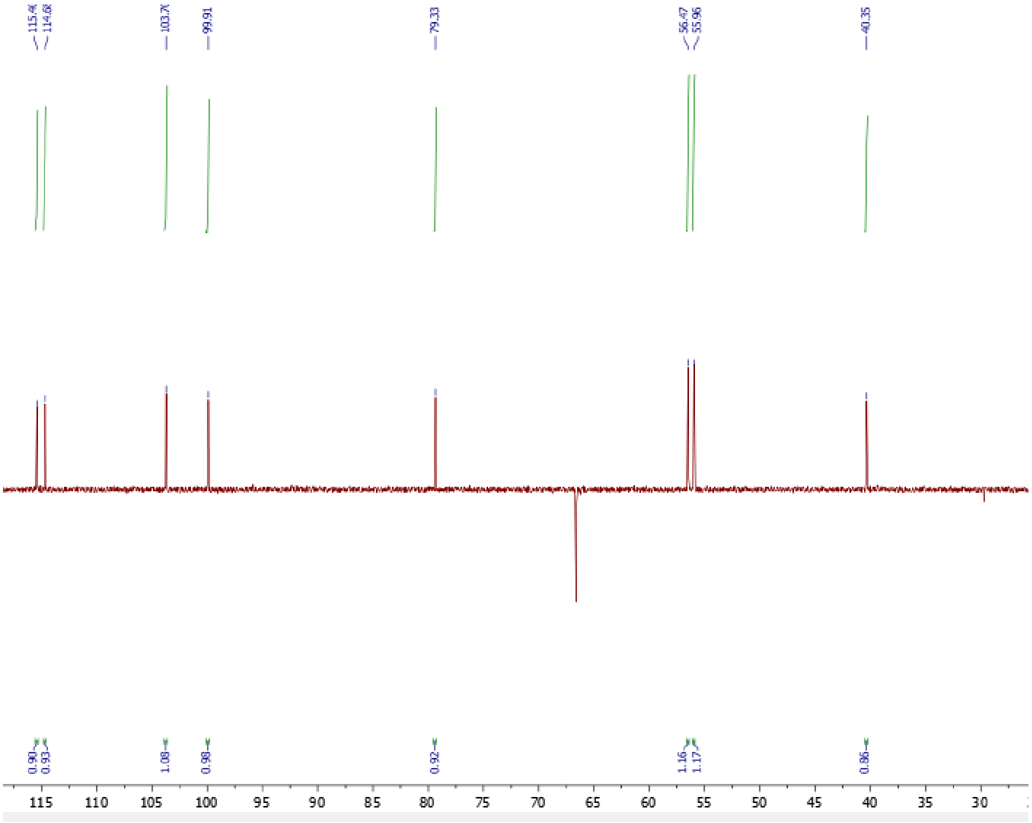

Figure S62. 135 DEPT (125MHz, $CDCl_3$) spectrum of **14**.

Elements Used:

Mass	RA	Calc. Mass	mDa	PPM	DBE	Formula	i-FIT	i-FIT Norm	Fit Conf %
317.1055	100.00	317.1084	-2.9	-9.1	0.5	C10 H21 O11	1356.5	3.537	2.91
		317.1025	3.0	9.5	9.5	C17 H17 O6	1353.0	0.030	97.09

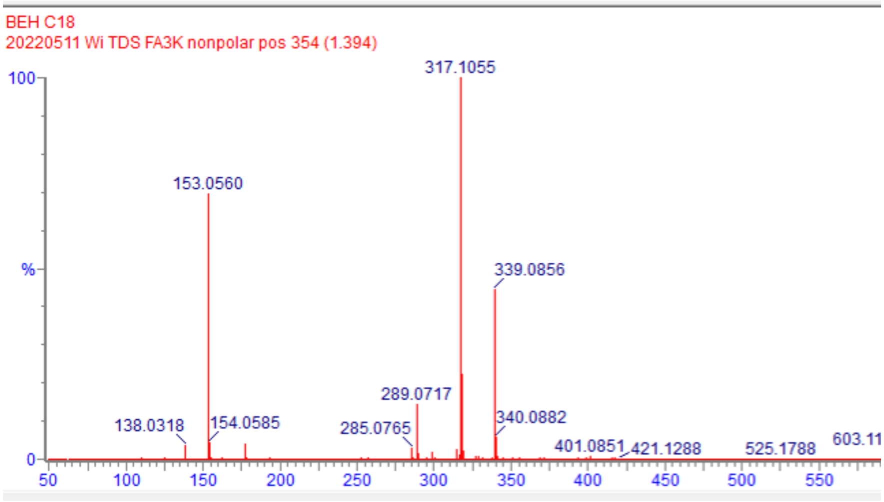

Figure S63. HRESIMS spectrum of **14**.

Ultra Performance Liquid Chromatography – Electrospray Ionization-Time-of-Flight Mass Spectrometry (UPLC-ESI-TOF MS).

Aliquots (1 µL) of the isolated compounds or fractions (1 mg/10 mL, 50% MeCN, each) were analyzed by means of UPLC-ESI-TOF MS on a Waters Synapt G2-S HDMS mass spectrometer (Waters, Manchester, UK) coupled to an Acquity UPLC core system (Waters, Milford, MA, USA) equipped with a 2 x 150 mm, 1.7 µm, BEH C18 column (Waters, Manchester, UK) consisting of a binary solvent manager, sample manager, and column oven. Operated with a flow rate of 0.4 mL/min at 50 °C, the following gradient was used for chromatography: starting with a mixture (1/99, v/v) of aqueous HCO_2H (0.1% in H_2O) and MeCN (0.1% HCO_2H), the MeCN content was increased to 99% within 4 min, kept constant for 1 min, decreased to 1% within 0.2 min, and finally kept constant for 0.8 min at 1%. Scan time for the MS^e method (centroid) was set to 0.1 sec. Analyses were performed with negative ESI in high resolution mode using the following ion source parameters: capillary voltage -2.0 kV, sampling cone 50 V, source offset 30 V, source temperature 120 °C, desolvation temperature 450 °C, cone gas flow 2 L/h, nebulizer 6.5 bar and desolvation gas 800 L/h. Data processing was performed by using Mass Lynx 4.1 SCN 9.16 (Waters, Manchester, UK), and the elemental composition tool for determining the accurate mass. All data were lock mass corrected on the pentapeptide leucine enkephaline (Tyr-Gly-Gly-Phe-Leu, *m/z* 554.2615, $[M-H]^-$) in a solution (1 ng/µL) of MeCN/0.1% HCO_2H (1/1, v/v). Scan time for the lock mass was set to 0.3 s, an interval of 15 s and 3 scans to average with a mass window of ±0.3 Da. Calibration of the Synapt G2-S in the range from *m/z* 50 to 1200 was performed using a solution of HCO_2Na (5 mmol/L) in 2-propanol/H_2O (9/1, v/v). The UPLC and Synapt G2-S systems were operated with MassLynx™ software (Waters, Manchester, UK). Collision energy ramp for MS^e was set from 20 to 40 eV.

HPLC separation

Preparative purification was performed on an HPLC system (Jasco, Groß-Umstadt, Germany) consisting of an HPLC-pump (PU 2080 Plus), a degasser (DG-2080-53 3-Line-Degasser), a DAD/UV detector (MD-2010 Plus), coupled with an autosampler (AS-2055 Plus) and equipped with a 250 x 21.2 mm, Varian Microsorb C-18 column (Darmstadt, Germany). Chromatography was performed with a flow rate of 21 mL/min with formic acid (0.1% in water, solvent A) and ACN (solvent B), starting at 50 % B for 3 min, increasing to 100 % B within 20 min, holding isocratically for 5 min, decreasing to 50 % B within 3 min, and finally equilibrating at 50 % B for 3 min. The effluent was monitored at 220 nm, and eluting substances were collected manually.
